# Resurrecting Darwin’s Niata - anatomical, biomechanical, genetic, and morphometric studies of morphological novelty in cattle

**DOI:** 10.1038/s41598-018-27384-3

**Published:** 2018-06-14

**Authors:** Kristof Veitschegger, Laura A. B. Wilson, Beatrice Nussberger, Glauco Camenisch, Lukas F. Keller, Stephen Wroe, Marcelo R. Sánchez-Villagra

**Affiliations:** 1 Palaeontological Institute and Museum, University of Zurich, Karl Schmid-Strasse 4, 8006 Zurich, Switzerland; 2 School of Biological, Earth and Environmental Sciences, University of New South Wales, Sydney, New South Wales 2052 Australia; 3 Department of Evolutionary Biology and Environmental Studies, University of Zurich, Winterthurerstrasse 190, 8057 Zurich, Switzerland; 4 Zoological Museum, University of Zurich, Karl Schmid-Strasse 4, 8006 Zurich, Switzerland; 5 Department of Zoology, School of Environmental and Rural Sciences, University of New England, Armidale, NSW 2351 Australia

## Abstract

The Niata was a cattle variety from South America that figured prominently in writings on evolution by Charles Darwin. Its shortened head and other aspects of its unusual morphology have been subject of unsettled discussions since Darwin’s time. Here, we examine the anatomy, cranial shape, skull biomechanics, and population genetics of the Niata. Our results show that the Niata was a viable variety of cattle and exhibited anatomical differences to known chondrodysplastic forms. In cranial shape and genetic analysis, the Niata occupies an isolated position clearly separated from other cattle. Computational biomechanical model comparison reveals that the shorter face of the Niata resulted in a restricted distribution and lower magnitude of stress during biting. Morphological and genetic data illustrate the acquisition of novelty in the domestication process and confirm the distinct nature of the Niata cattle, validating Darwin’s view that it was a true breed.

## Introduction

**Figure 1:**
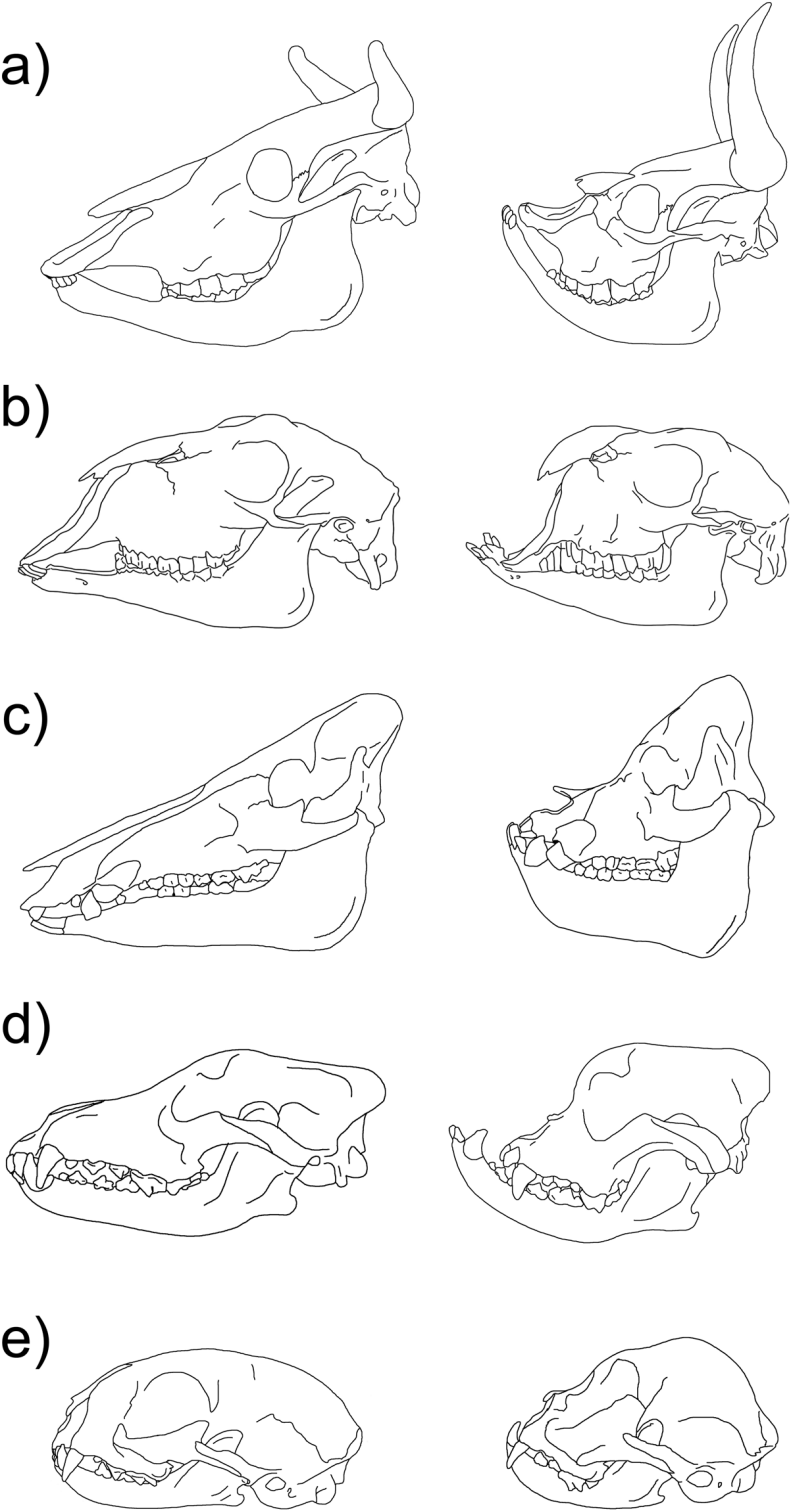
Lateral view of the skull in ancestral (left) and brachycephalic (right) conditions in several domesticated mammals, including the Niata cow. (**a**) Cattle; Niata specimen based on NHMD-ZMK-MK-1109 from Copenhagen. (**b**) Goat, Egyptian goat (based on NMW 2073 from Vienna). (**c**) Wild boar, brachycephalic pig breed. (**d**) Wolf, Bulldog (**e**) Cat, Siamese cat (based on Schlueter *et al*.^72^). Based on specimens at the Palaeontological Museum Zurich; drawings by K. Veitschegger and Tímea Bodogán.

The change in skull shape towards a short and stout appearance (brachycephaly) is an independently reappearing trait during domestication and is considered a breed-defining characteristic in some dogs, cats, and pigs^1^ (Fig. 1). In cattle, extreme brachycephaly was described in the Niata cow from South America^2^. In the Niata, brachycephaly was considered breed defining^2^ even though heavily shortened skulls have been observed in several cattle breeds as a form of malformation^2–6^. The first documented encounters of European explorers with the Niata are from the early 19^th^ century^7,8^. However, the Niata only received widespread recognition after the publication of Charles Darwin’s second edition of the Journal of Researches in 1845^2^. Darwin described the appearance of the Niata and speculated on its biology. Subsequently, the Niata became a controversial subject among scientists, especially in France^3,9–16^. The skull configuration of the Niata resembled that of individuals with malformations, thus much of the aforementioned discussions on the nature of the Niata revolved around this false dichotomy between malformations and breeds, still found in literature today^1,17^. Lenient definitions of breeds simply require the human intent to select for and preserve a certain morphology within a population that distinguishes it from other members of the species^1^.

The anatomy of the Niata has not been properly described, in particular as it compares to malformation syndromes, and the issues raised since Darwin’s writings have not been resolved. Current methods allow us to do so, while benefiting also by the study of DNA data. We investigate the anatomy and body size of the Niata and evaluate the first description of its skull by Owen^18^. Additionally, we use geometric morphometrics of the skull and genetic data to place the Niata within the context of other cattle breeds.

Our anatomical study will serve to evaluate the claims that the morphology of the Niata is tied to disease and malformations^14^. Dareste made the first connection between chondrodysplastic bulldog-calves, also known as snorter dwarfs, and the Niata cattle^3^. Chondrodysplasia is a congenital disease, which leads to shortened limbs, brachycephalic skulls, and an early fusion of the spheno-occipital synchondrosis in cattle, and it can be lethal^6,19^. However, mutations in genes associated with chondrodysplasia can also be important drivers in the appearance of breeds^20^ and in dogs different genes are related to chondrodysplasia and brachycephaly, respectively^20–22^. In cattle, several genes such as ACAN, PRKG2, and EVC2 are considered to be related to so-called snorter dwarf-like chondrodysplasia^6,23,24^, which is even breed defining in Dexter cattle^6^. Here, we examine the cranial and postcranial morphology of the Niata to compare it with anatomical markers for chondrodysplasia in cattle.

We extracted DNA from Niata specimens in museum collections and genotyped single nucleotide polymorphisms (SNPs) to study the relationship between the Niata and other cattle breeds such as indicine Zebu breeds, taurine European breeds, hybrids, as well as *Bos javanicus*^25^.

In addition to examine the issues relating to the anatomy and the state of the Niata as described above, we examined an issue pertaining the extinction of the Niata. For Darwin, the Niata was an illustrative example for selection^2,26^. He speculated that the Niata must have experienced disadvantages during droughts because its extreme brachycephaly with the projecting lower jaw would have negatively affected feeding. We explored Niata biomechanics using Finite Element Analysis (FEA) to examine the stress distribution over the skull during biting.

## Methods

### Cranial and postcranial anatomy

Skull measurements were taken according to von den Driesch^27^ and terminology follows Budras *et al*.^28^. We used a μCT scan to assess the configuration of the nasal conches (supplementary information); manual segmentation of the nasal conchae was performed in Avizo 8.1.

The pattern of external suture obliteration in 26 sutures was documented in 47 cattle skulls of known age (Supplementary Fig. 1, Supplementary Table 1) and in four skulls of Niata cattle (Supplementary Table 1). A picture of the Niata skull NHMD-ZMK-MK-1109, provided by K. M. Gregersen (NHMD), was used for assessment of the spheno-occipital synchondrosis. The age of specimens was estimated based on tooth eruption, incisor wear pattern as well as information from literature^29,30^. External suture obliteration was documented in three stages. A suture was open when no signs of obliteration were visible; this was scored as “0”. Any degree of closure was scored as “1” and a completely obliterated suture was scored as “2”. The overall average of the scores was compared with individual age. Basicranial angle was measured in 77 skulls from 36 breeds (Supplementary Table 2). Four landmarks were used to measure the basicranial angle: suture between premaxillary and maxillary bone in the midline (1), tip of the palatine bone in midline (2), suture between vomer and presphenoid in midline (3), and ventral border of the foramen magnum in midline (4) (Supplementary Fig. 2). These four landmarks were visualized in lateral view using Morphologika 2.5^31^; a wireframe was drawn between landmark 1 and 2 as well as landmark 3 and 4. The angle was measured between these two lines. Resulting angles were averaged per breed. The body mass of the Niata was calculated by using and averaging different cranial and postcranial measurements (Supplementary Table 3)^32–34^. The height of the Niata was taken from Baldassarre^29^. The resulting average weight and the height were compared with those of 264 different taurine breeds (208 male and 262 female) as well as the aurochs, *Bos primigenius* (Supplementary Table 4). The comparative data for height and weight were taken from Felius^17^. Measurements used for body proportion comparison are shown in Supplementary Fig. 3.

### Geometric morphometric analysis

The data were obtained using a Microscribe digitzer (Microscribe MX, Immersion Corporation, San Jose CA) by the first author. 53 landmarks were used to capture skull shape. Custom frame was used in MUS 6.0.1 to digitize the dorsal and ventral part of the skull (Supplementary Fig. 4, Supplementary Table 5). Twelve landmarks were used to analyze the general shape of the left half of the lower jaw. The shape of the skulls and lower jaws was analyzed using geometric morphometrics^35–37^, using geomorph^38^ as implemented in R, version 3.2.3^39^. Initially, a Generalized Procrustes Analysis was performed to align the specimens and remove size^40^. We used centroid size to remove a possible effect of allometry in our dataset^38^. After creating the allometry-free shapes, we performed a Principal Component Analysis. At the beginning of data collection, one skull and one lower jaw were digitized five times to estimate error of precision^31^. Observer error was small compared to the variation within the dataset. To assess the degree of morphological difference between the Niata skull shape and other breeds, we calculated the Procrustes distance between mean skull shapes of breeds. Procrustes distance is the least squared mean distance between shapes after Procrustes alignment^40^. Altogether, the final dataset consisted of 316 adult skulls representing 86 breeds (four landraces, seven crossbreeds, four Zebu varieties, two Sanga breeds, and *Bos primigenius*) as well as 238 adult lower jaws from 65 breeds (including one landrace, one crossbreed, two Zebu varieties, one Sanga breed, and *Bos primigenius;* see supplementary material for results on lower jaw shape analyses).

### Finite Element Analysis

Finite Element Models (FEMs) were constructed from computed tomography (CT) scan data collected for three specimens (Supplementary Table 6). Body mass (BM) for each specimen was estimated using measurements of the lower molar tooth row length (LMRL), second lower molar length (SLML), anterior jaw length (JLB) and posterior jaw length (JMA), following the equations developed on ungulates by Mendoza *et al*.^41^. Postcranial material was available for MLP 1126, allowing body mass to be estimated from the circumference of the femur and humerus, following the equation for quadrupedal mammals presented by Anderson *et al*. and Scott^32,33^. The average of skull and postcranial body mass estimates was used for MLP 1126.

The major jaw closing muscles (masseter, temporalis, pterygoideus lateralis and pterygoideus medialis mm) were modelled for each specimen, their origin and insertion points were defined using a bovine anatomical atlas^28^. Muscle forces were predicted on the basis of maximum cross-sectional area (CSA) using the ‘dry skull’ method and CSA values were multiplied by the tension value for vertebrate striated muscle of 0.3 N/mm^2^ ^42,43^. The CSA values were summed for the four muscle groups, and the relative proportion of each muscle was calculated; these data were compared to muscle mass values for ungulates^44^. Muscle forces were distributed in each model based on the percentage contribution of each muscle group to the total (Supplementary Table 7).

Extensive details on the methods for muscle forces scaling, for Finite Element Model (FEM) assembly and for comparison of biomechanical performance are provided in the supplementary information (see Supplementary Fig. 5)^45–52^.

### Analysis of SNP data

We collected five samples (tooth, bone or tissue) of the Niata in the Museo de La Plata (MLP) and the Museo Argentino de Ciencias Naturales (MACN) of Buenos Aires. We extracted the DNA following the protocol of Kruettli *et al*.^53^, which includes cleaning the sample with 2% NaOCl to remove surface contamination, a 48 hours digest of pulverized material in 0.45 EDTA and 10% proteinase K, DNA isolation using QiaQuick PCR purification columns and DNA elution in EB buffer. Samples were genotyped with the GeneSeek® Genomic Profiler Bovine 150 K SNP chip and gene called with the Illumina® GenomeStudio V2011.1 software. As expected from the degraded DNA typical of museum specimens, the average genotype quality scores (GenCall) were low (~0.3) in all our samples, except in one (MACN-Ma 25.162). To obtain a dataset with as little genotyping error as possible yet powerful enough to infer the relationship of Niata cattle with other cattle breeds, we applied a GenCall cut off score of 0.5. This yielded 2’205 SNPs (i.e. a genotyping rate of 6.2%) which were also genotyped in 134 cattle breeds by Decker *et al*.^25^. This number is similar to the number of SNPs used by McTavish *et al*.^54–56^. We inferred the relationship between the Niata samples and 134 cattle breeds using a principal component analysis (PCA) and phylogenetic trees. The PCA was implemented in PLINK 1.9 and plotted using R^39^. We inferred phylogenetic trees using TREEMIX^57^. To evaluate the robustness of our inferences we also ran the TREEMIX analysis with a subsample of 88 breeds that excluded most indicine breeds. All TREEMIX analyses were run with and without migration branches. Since the analyses did not reveal solid evidence for migration branches involving Niata cattle, we only present the results without migration branches. Maximally five individuals per breed, the first ones in the list of samples given by Decker *et al*.^25^, were included in all these analyses.

### Data, code and materials

The datasets supporting this article have been uploaded as part of the supplementary material.

## Results

### Cranial and postcranial anatomy

**Figure 2:**
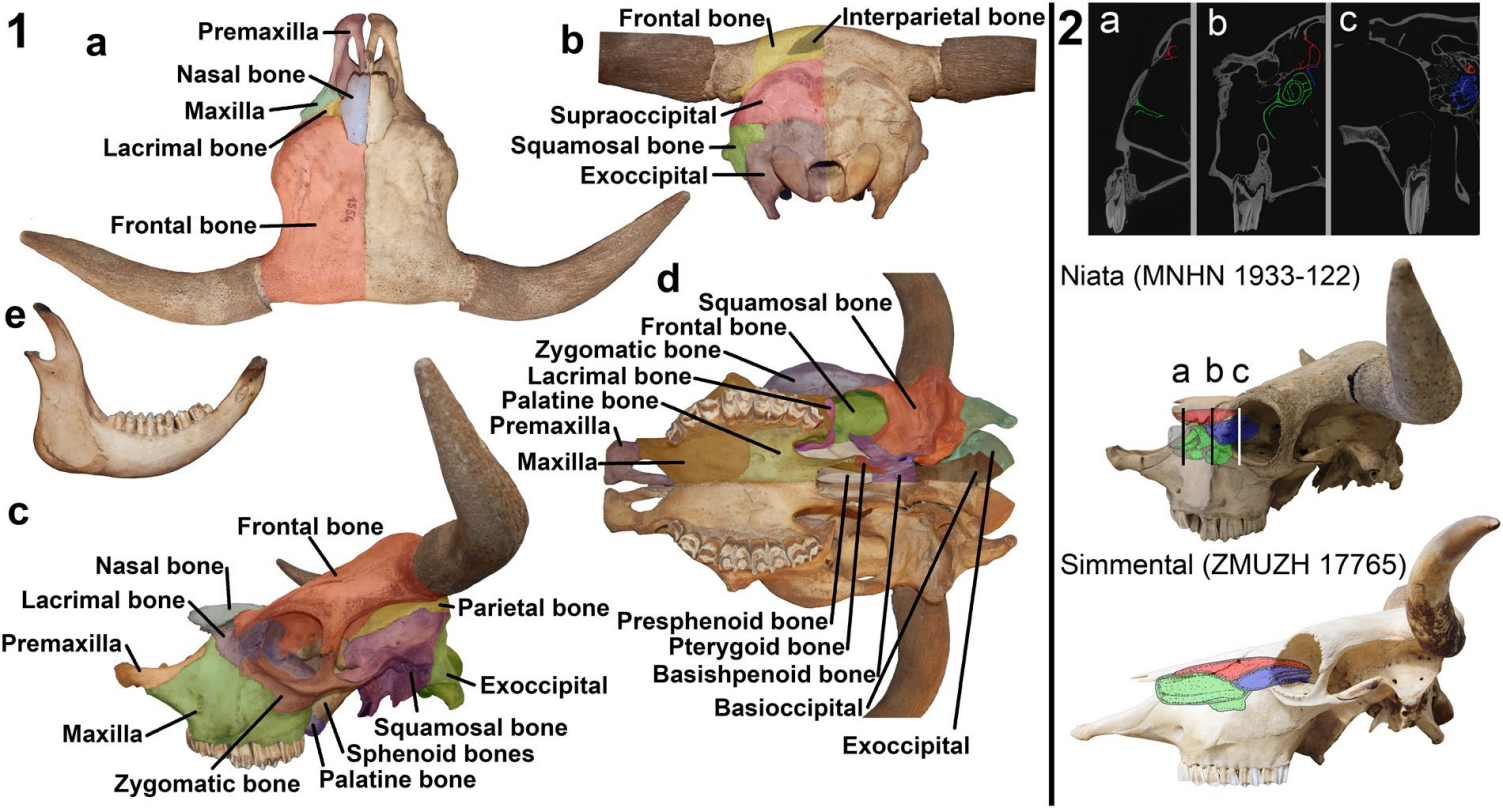
Skull and lower jaw of the Niata (MLP 1556). (**1a**) skull dorsal view, (**1b**) skull posterior view, (**1c**) skull lateral view, (**1d**) skull ventral view, (**1e**) lower jaw lateral view. 2: schematic reconstruction of the dorsal (red), middle (blue), ventral (green) conches in the Niata (broken parts are reconstructed in grey) based on CT scan data (mirrored).

The skull of the Niata is short and broad, as is typical for a brachycephalic condition (Fig. 2, Supplementary Table 8). The maxillary bone of the Niata has a triangular outline in lateral view due to the close approximation, or direct contact, of lacrimal and premaxillary bone. It is shortened and the facial crest cannot be easily distinguished; the facial tuber is very prominent. The alveolar process towards the teeth is convex and the diastema is short. The shortening of the maxillary bone has also changed the angle between premolar and molar teeth. The premolar teeth are placed in an obtuse angle to the molars, as is also the case in the lower jaw. In the premaxillary bone, the alveolar process and the body of the incisive bone are short and stout. The premaxillary bones are medially curved upwards so that the process is V-shaped from anterior view. The nasal process is variable in length, but in three of the five examined skulls, it comes into contact with the lacrimal bone. The shortening of the maxillary bone, therefore, slightly changed the configuration of the sutures in the skull. The nasal bones of the Niata are short, stout, and are convex in lateral view. The suture between the two nasal bones fuses in the Niata and in one of the examined specimens it was not visible anymore. Due to the shortening of the snout region, the dorsal, middle, and ventral nasal conchae have become proportionally short and stout (Fig. 2). No conch has been affected more strongly than any other. The frontal bone of the Niata is curved upwards to the nasal bones. The intercornual protuberance can be from W-shaped to flat in dorsal view. The length of the frontal bone from the nasal-frontal suture in midline (nasion) to the posterior edge of the skull in midline (acrocranion) is rather short compared with the smallest length of the frontal bone. This gives the frontal bone a squarish appearance. At the base, the bony part of the horns of the Niata grows in lateral direction and then turns antero-dorsally. In individuals with longer horns, there is a second postero-dorsal turn. The orbits of the Niata are square-shaped and, due to the shortening of the snout, placed more anteriorly. The jugal bone and the lacrimal bone are shortened towards the maxillary bone. The morphology of the occipital bone and temporal fossae do not differ between the Niata and other cattle. The craniobasal angle of the Niata is over 180°, the airorhynch condition (Supplementary Table 2). The lower jaw is curved upwards and the incisors project over the body of the incisive bone of the premaxillary bone, leading to malocclusion (supplementary Figs 5 and 15)^2^.

**Figure 3:**
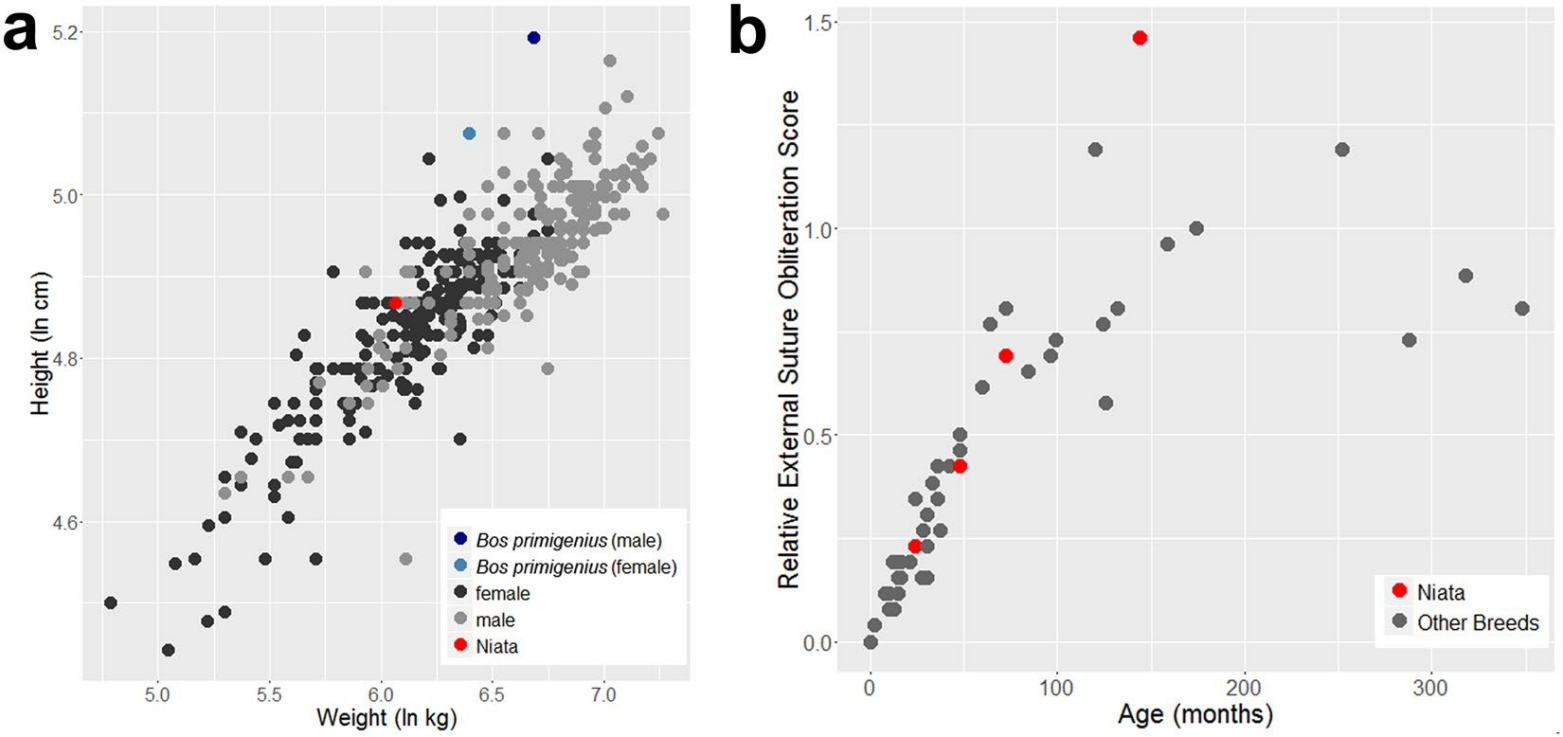
(**a**) Body size of the Niata cattle compared to other taurine cattle breeds. (**b**) Relative external suture obliteration scores at different ages in the Niata and other cattle breeds.

The external suture obliteration pattern of the cranial sutures of the Niata is not different from that observed in other cattle (Fig. 3). The Niata skull with the oldest estimated individual age (MLP 1126, about 12 years), however, shows a high degree of external suture obliteration. The spheno-occipital synchondrosis remained open in a specimen with an estimated age of about 24–30 months based on the shedding of deciduous teeth^30^ but was closed in the individual NHMD-ZMK-MK-1109, which exhibits a slightly more advanced tooth eruption (Supplementary Fig. 6). All other Niata specimens exhibit a fused spheno-occipital synchondrosis and permanent dentition.

The Niata was of average size when compared to other taurine breeds (Fig. 3, Supplementary Table 3). The body mass of the Niata was reconstructed based on the complete skeleton MLP 1126, a male^29^. The comparison of the ratio of limb length and cervical-sacrum length among breeds shows that the Niata did not have short limbs compared to its body length, nor are the hind limbs disproportionally longer than the forelimbs (Supplementary Figs 7 and 8; Supplementary Tables 9, 10).

### Morphometric analysis

**Figure 4:**
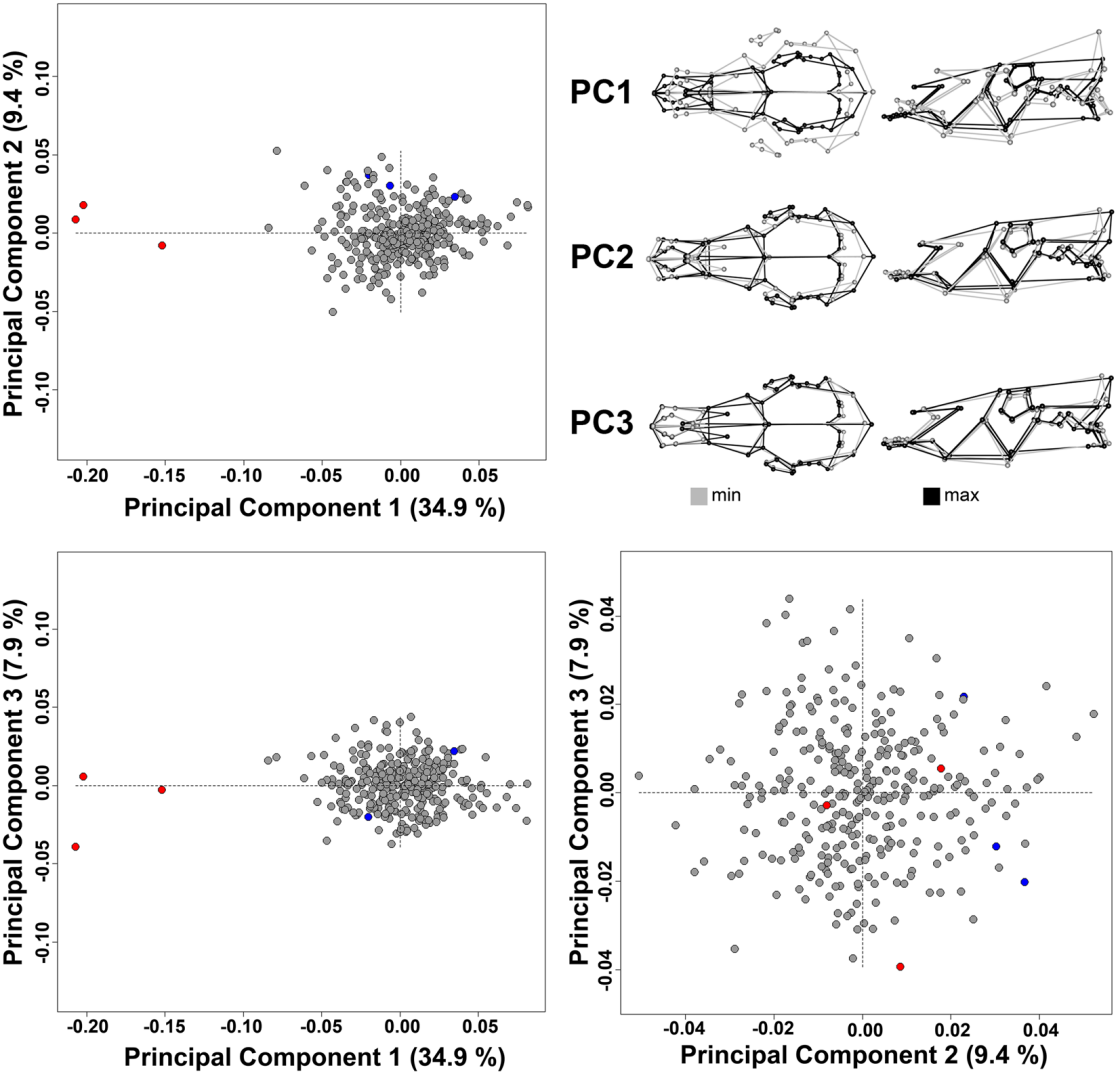
Principal Component Analysis of the skull shapes of different cattle breeds. Top left, bottom left and bottom right are the plots between PC1, PC2, and PC3. Top right are the associated shape changes. Numbers in parentheses indicate shape variation explained by respective PC axes. Red dots indicate the Niata and blue dots the aurochs (*Bos primigenius*).

The landmark-based morphometric analysis of the skulls clearly shows the singularity of the Niata (Fig. 4). Principal component (PC) 1 accounted for 34.9 percent of the overall shape variation and is defined by the difference between short skulls (brachycephalic) and the elongated ones (dolichocephalic), as found in Sanga and Zebu cattle. The second PC axis was defined by differences relating to the angle between the nasal and frontal bones as well as the broadness of the skull. This axis accounted for 9.4 percent of the shape variation. The third PC axis accounted only for 7.9 percent of the shape variation and is mainly defined by extension of the nasal process of the premaxillary bone. No further PC axis accounted for more than five percent of the shape variation in the sample (Supplementary Fig. 9a). Overall, the Niata was located away from other breeds in morphospace, evidenced by large Procrustes distances (PD) for pairwise comparisons. The mean shape of a Niata skull was most different from Bucharan Grey (PD: 0.29), Sanga (PD: 0.26), and Zebu (PD: 0.25) cattle and closest to Tuxer (PD: 0.15), Zillertaler (PD: 0.17), as well as Jersey (PD: 0.17) cattle. In comparison, Maas-Rhein-Ijsselschlag cattle, a breed with a skull shape closer to the overall mean shape, showed smaller Procrustes distances. It was most different in shape to the Niata (PD: 0.21), the Bucharan Grey (PD: 0.11), and the Tuxer (PD: 0.08) cattle and more similar to the Devon (PD: 0.02), North Wales (PD: 0.02), and Bohemian Red (PD: 0.02) cattle (Supplementary Table 11). A similar pattern was recorded for the lower jaw (for details see Supplementary Information; Supplementary Figs 10 and 11; Supplementary Tables 12, 13).

### Finite Element Analysis

Overall, the VM stress contour maps indicated some similarities across all breeds, particularly the high VM stress values along the zygomatic arch for all loading cases, however some differences were also present, most markedly between the Niata and Zebu in the orbital and supraorbital region (Table 1).

**Table 1:** Overview of differences in Von Mises (VM) stress magnitudes between the Niata, Simmentaler and Zebu breeds. See supplementary text for further comparison between loading cases.

Loading case	Location on the cranium	VM stress value (comparative)		
		**Niata**	**Simmentaler**	**Zebu**
Bilateral bite	Dorsal surface of the frontal, and supraorbital sulcus	lower	higher	higher
	Lateral margin of the frontal and squamosal	lower	higher	higher
	Zygomatic arch	high	high	high
	Temporomandibular joint (TMJ)	high	higher	highest
	Dorsal midline	lower	higher	highest
	Tooth row	lower	higher	higher
Unilateral bite	Dorsal surface of the frontal, and supraorbital sulcus	lower	higher	higher
	Lateral margin of the frontal and squamosal	lower	higher	higher
	Zygomatic arch	high	high	high
	Maxilla-zygomatic suture region	high	higher	higher
	Rostrum	high	high	high
	Temporomandibular joint (TMJ)	high	higher	highest
	Dorsal midline	lower	higher	highest
	Tooth row	lower	higher	higher

**Figure 5:**
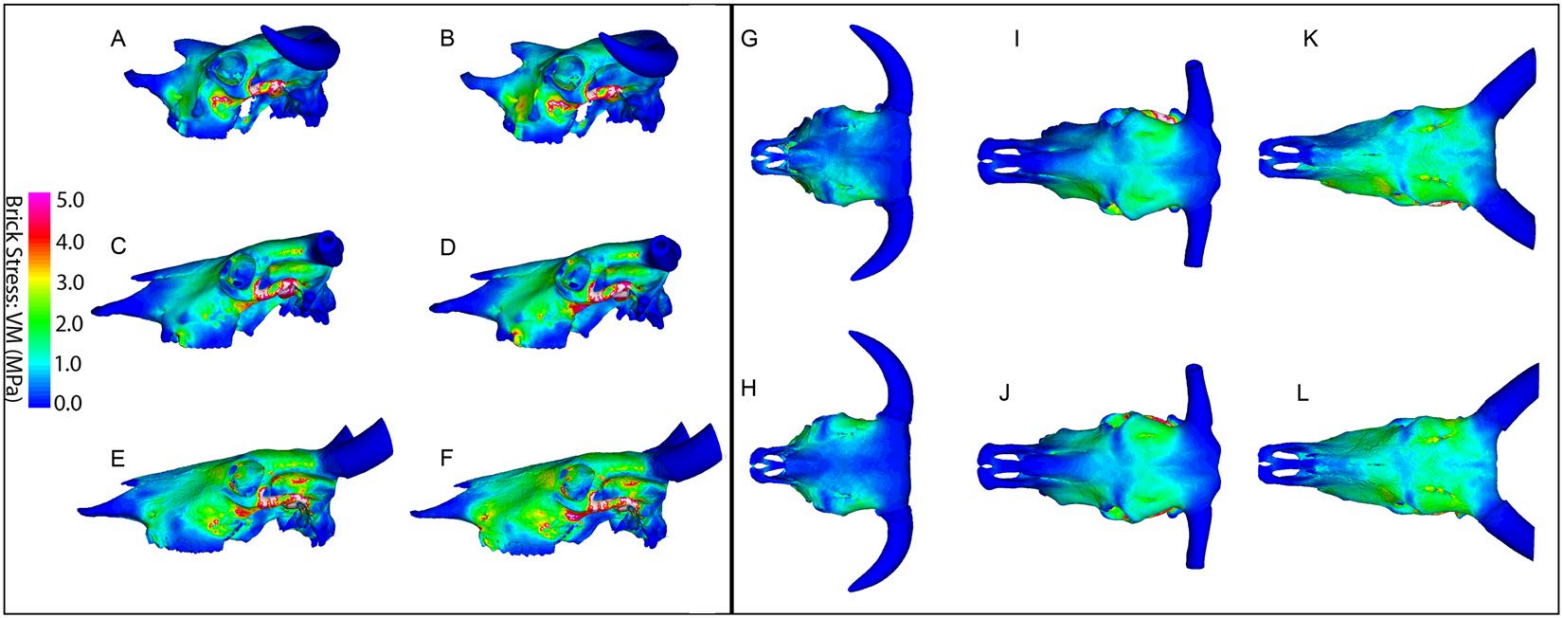
Lateral and dorsal finite element models: Contour mapping of Von Mises (VM) stress distributions in lateral view. Models were subjected to loading cases simulating a bilateral anterior bite (**A**,**C**,**E**,**G**,**I**,**K**), and a unilateral anterior bite (**B**,**D**,**F**,**H**,**J**,**L**). Models are for Niata (**A**,**B**,**G**,**H**), Simmentaler (**C**,**D**,**I**,**J**) and Zebu (**E**,**F**,**K**,**L**). Muscle forces were scaled to body mass for each model. White regions exceed the scale.

Across all loading cases, the Niata cranium exhibited comparatively low stress levels, particularly across the dorsal surface of the frontal and the supraorbital sulcus (Fig. 5G,H). Compared to the Simmentaler and Zebu, the Niata cranium showed lower stress along the lateral margin of the frontal and squamosal (Fig. 5A,C,E).

For the unilateral bite, all breeds showed greater VM stress values in the rostrum on the working-side compared to the balancing-side (unloaded), especially for the maxilla and lacrimal region, as well as slightly increased stress for the working-side compared to the balancing-side dorsal surface of the frontal (Fig. 5H,J,L). Comparing the VM stress contour maps for the working-side unilateral bite and the bilateral bite in lateral view, for all breeds the results show that the unilateral bite leads to increased VM stress in the anterior portion of the maxilla and near to the suture between the maxilla and zygomatic (Fig. 5). Compared to Zebu and Simmentaler, the Niata displays only a slight increase in magnitude and extension in distribution of VM stress near to the maxillo-zygomatic suture (Fig. 5A,B).

Consistent with the contour map results, strain results collected at the temporomandibular joint (TMJ) for the unilateral loading case indicated that overall the Niata cranium showed the lowest VM strain values (218.05–218.76 μɛ; Supplementary Table 14). The difference between VM strain values for the working-side TMJ compared to the balancing-side TMJ was lowest for the Niata (0.32%), and highest for the Zebu (9.5%) (Supplementary Table 7). The predominant mode of strain at the TMJ was compressive.

Among the breeds, the Niata exhibited overall lower VM stress along the mid-sagittal plane of the cranium (average = 0.31 MPa; Simmentaler, average = 0.37 MPa; Zebu, average = 0.50 MPa) (Supplementary Fig. 12A). The difference in stress between the Niata and the other breeds was most conspicuous at points 3–6 for the bilateral bite, corresponding to the area extending from the posterior part of the nasal, beyond the naso-frontal suture, towards the orbits (Supplementary Fig. 12A). Along the dorsal midline, the Niata displayed more variation in stress values between the unilateral and bilateral bite than Zebu and Simmentaler, particularly showing greater stress in the unilateral bite for points 4–8 (Supplementary Fig. 12A).

Overall, the pattern of VM stress values was more similar among the breeds along the tooth row than along the dorsal midline (Supplementary Fig. 12). VM stress values were, on average, higher for the tooth row equidistant points (0.51–0.68 MPa) compared to those collected along the mid-sagittal plane (0.27–0.50 MPa). For all breeds, differences between the bilateral and unilateral bite were generally most marked towards the anterior portion of the tooth row, corresponding to points 1–5 (Supplementary Fig. 12B). The Simmentaler and Zebu displayed greater differences between stress values for the bilateral bite and unilateral bite along the tooth row than the Niata.

The contour maps of VM stress in dorsal view indicated that, for both loading case simulations (unilateral molar bite at M2, anterior bilateral bite at M1), the Zebu cranium displayed high stress levels when compared to the other breeds, especially the Niata (Fig. 5A compared to 5E and 5G compared to 5K). The frontal and nasal bones experienced comparatively more stress in the Zebu, especially along the midline compared to the other breeds (Fig. 5G,I,K). For all loading cases in lateral view, the Zebu showed greater VM stress extending across the frontal and maxilla than did the Simmentaler and Niata, and especially towards the suture between the maxilla and zygomatic in both loading cases (Fig. 5A–F).

The Zebu showed markedly higher VM stress (average = 0.59 MPa) at the most an-terior point of the nasal and at the most posterior point sampled (average = 0.55 MPa), compared to the values for the Simmentaler (point 1, average = 0.04 MPa; point 10, average = 0.12 MPa) and Niata (point 1, average = 0.24 MPa; point 10, average = 0.11 MPa).

### Analysis of SNP data

**Figure 6:**
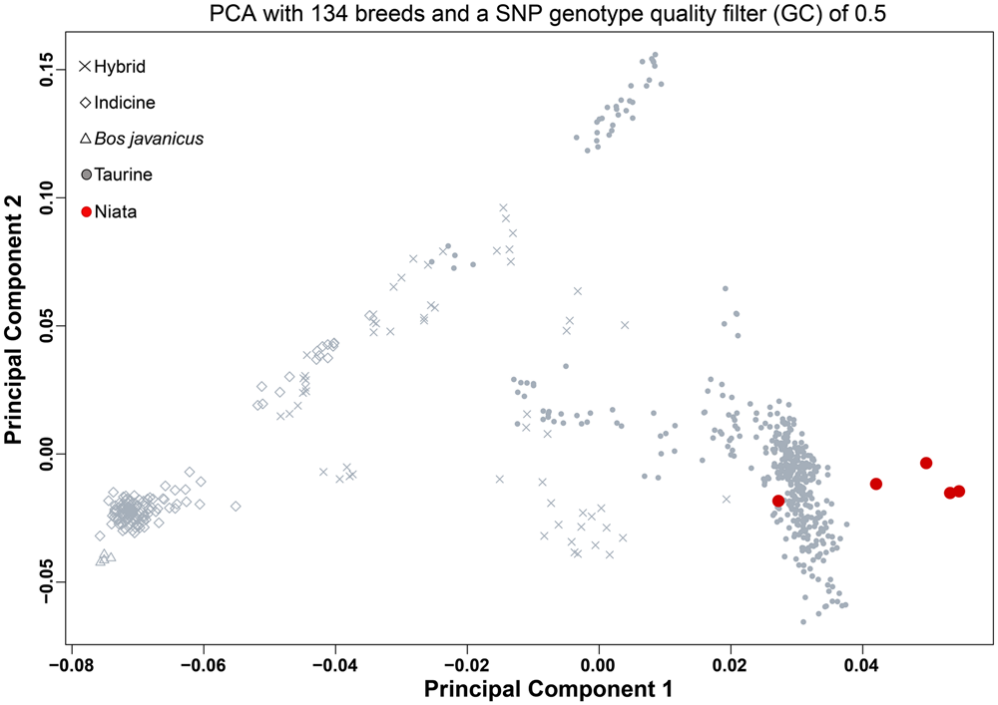
Principal Component Analysis based on 2’205 SNPs of the relationship between the Niata samples and 134 cattle breeds presented in Decker *et al*.^24^. PC1 and PC2 explained 51.6% and 9.5% of the variance, respectively.

Using data from 2’205 SNP loci genotyped in this study in five Niata samples and in 134 other cattle breeds by Decker *et al*.^25^, it is evident that Niata cattle form a genetic cluster within the taurine breeds. Both principal component analysis (Fig. 6) and phylogenetic analysis (Supplementary Figs 13 and 14) confirm this view. Of all the breeds included in our analyses, Niatas cluster furthest away from the indicine breeds. Furthermore, Niata samples do not cluster with Iberian breeds (e.g., Morucha) or South American breeds (e.g., Corriente), nor with breeds known for the occurrence of some forms of chondrodysplasia (e.g., Dexter, Angus, Hereford, Kerry)^6,17,19,23^. Instead, they form their own, independent cluster, suggesting that Niata can be considered a separate breed. However, one of the Niata samples (MACN-Ma 25.162) clustered between European and American breeds, and may not represent the same gene pool as the other Niata samples. We could not determine the closest relative of the Niata breed because the exact branch location of Niata was not reproducible in phylogenetic analyses with different parameters (Supplementary Figs 13 and 14).

## Discussion

### Anatomical evidence and signs of chondrodysplasia

The Niata exhibits a brachycephalic skull and reveals morphological singularities, as well as showing some differences in the distribution and magnitude of stress compared to the other sampled breeds (Supplementary Fig. 15). The basicranial angle is an important feature related to brachycephaly in dogs. The wild ancestor of dogs, the grey wolf (*Canis lupus*), has a basicranial angle of about 170°, in the middle of the range between the Barsoi (158°) and the brachycephalic French bulldog (183°) breeds^58,59^. For cattle, we found a transition in the basicranial angle from an ancestral condition of around 158°, as found in the aurochs (*Bos primigenius*)^17^, to an angle of about 193° in the Niata. All examined breeds fall within these two extremes. Future work could assess the variation of aurochs in this regard^60^ and the significance of this change with its domestication.

Suture configurations in some individuals diverge from the norm for cattle, e.g. the lacrimal bone coming into contact with the premaxillary bone, although this is not universal in Niata skulls (*contra Owen*^18^). The timing of the fusion of the spheno-occipital synchondrosis in the Niata has not been affected by the change in the skull configuration^61^. This is of particular importance, as an early fused synchondrosis has been shown to be characteristic for chondrodysplastic malformations in cattle^19^. In individuals affected with the snorter dwarf condition the synchondrosis fuses at around 5.5 months^19^. In dogs, brachycephalic breeds are more likely to have a relatively early closed spheno-occipital synchondrosis^62^. Generally, the external suture obliteration pattern of the Niata is within the range of other cattle of similar age until the age of 72 months; the oldest skull (MLP 1126) has the highest external suture obliteration score. This is in agreement with a pattern found in brachycephalic dogs^59^. However, external suture obliteration patterns are more variable in older cattle^61^. Sutures have an important role in mitigating stress on the skull, therefore an early fusion can lead to irregular bone growth^63^. In contrast to skull singularities, the body size and limb proportions of the Niata show no delineations from other cattle. In Dexter cattle short limbs are one of the breed defining characteristics^6^. The average proportions of Niata do not support Darwin’s proposition of disproportionally short front limbs (Supplementary Fig. 7, Supplementary Tables 9, 10)^2^.

### Geometric morphometrics and genetic evidence

The Niata occupies a unique position in the morphospace of cattle skulls, as evidenced by large Procrustes distances revealed in the morphometric analysis. The breeds most similar to the Niata are the Tuxer and Zillertaler, which were alpine cattle varieties originating from Austria^17^. Early works reported similarities between the Tuxer and the Niata^64,65^. Another similar breed is the Jersey^17^, which, like the Niata, has an upward curved forehead. The furthest removed from the Niata skull shape are the Bucharan Grey, followed by Zebu and Sanga cattle. Zebu and Sanga are dolichocephalic breeds, which originated from another domestication area than most European breeds^66^. The first cattle that were brought to the Americas were from Iberian taurine origin and intermixture with indicine breeds did not happen before the mid-19^th^ century^67^. Based on this knowledge of the origin of South American cattle breeds^54–56^, it is of particular interest that the Niata does not cluster with any Iberian breed within the dataset such as the Tudanca or Rubia Gallega. The distinct position of the Niata is confirmed in the PCA based on SNP data. Here, the Niata individuals form a cluster that slightly overlaps with the taurine breeds (Fig. 6).

### Finite element analysis

Darwin suggested that the Niata had a disadvantage compared to other cattle breeds when foraging. He stated that the protruding lower jaw of the Niata inhibits it from properly browsing twigs or reeds in case of droughts, thus leading to its demise^2^. There is no preserved soft tissue of the Niata, nor is there any depiction of a feeding Niata that would allow us to determine precisely what effect the protruding lower jaw had on the process of feeding^68,69^. A longue tongue may have been needed in the Niata to cope with the underbite of the lower jaw while eating grasses from the ground as opposed to browsing. What we can evaluate is the mechanical performance. Our FEA results show that the skull of the Niata experienced lower magnitudes of stress during both anterior bilateral biting and unilateral molar biting compared to the other breeds considered, and most markedly in comparison to the Zebu. These results are consistent with predictions, from traditional beam theory, that under adduction of the jaws during biting, the rostrum will behave as a cantilevered beam loaded in the dorso-ventral direction^70^. As such, longer rostra are predicted to show greater magnitudes of stress, reflected in the greater values of stress for the Zebu compared to the Niata. The distribution of stress in the Niata cranium was also more restricted, with an absence of high stress across the frontal and maxilla (contra the Zebu and Simmentaler). In the latter case, it is noteworthy that the Zebu also showed high stress in the orbital and superorbital region, areas that are not optimized for maximizing strength with minimal material (Ross and Iriarte-Diaz 2014) and that serve to protect the brain and eyes foremostly rather than impacting performance of feeding behavior. The shortening of the skull in the niata, as seen among other domesticated animals (e.g. rabbit^68^), would be expected to result in more vertically oriented muscle fibers, leading to a greater vertical bite force generated at the cheek teeth^69^. The latter would improve the ability of the Niata to efficiently process foods requiring crushing between the teeth, an activity that generates axial strain on the molars.

### The Niata in context of breeds

The origin and fixation of the Niata traits in South American cattle was most likely not intentional but rather the result of a very small founding population^55,56^. Darwin^2^ already wrote about the heritability of the Niata traits and he asserted that they breed true. Empirical evidence for the heritability of the Niata traits comes from breeding experiments in Jersey cattle^4^. These experiments also showed that heavily brachycephalic cattle suffer from heritable impaired vision^4^. Inherited illnesses are a common phenomenon in modern brachycephalic dog and cat breeds^71,72^.

The Niata is an illustrative case of how domestication can expand the morphospace of a species. In cattle ‘extreme’ or very derived anatomical traits appear to be discriminated against, which may be due to the utility value of cattle, e.g., being efficient dairy and meat producers as well as work animals^1,73^. Therefore, a breed with possible inherent health problems and probably difficult temper, as stated by Darwin, would be less appreciated^2,4^. This aspect of selective breeding can be easily exemplified by the different perception and recognition of heavily brachycephalic dogs such as French bulldog or the Boxer as true breeds^1^ whereas the status of the Niata as breed has been controversial. Early works dismissed the possibility of such a breed completely^14^ and in recent years it still was described as a defective form^17^. However, following a standard breed definition^1^ (p. 40): *“A breed is a group of animals that has been selected by humans to possess a uniform appearance that is inheritable and distinguishes it from other groups of animals within the same species,”* the Niata can be considered a breed. Our genetic and morphometric evidence clearly shows the distinctiveness of the Niata as true breed as suggested originally by Darwin^2^. The case of the Niata, which vanished sometime in the late 19^th^ or early 20^th^ century^29,74^, is an example for the loss of genetic diversity caused by the extinction of breeds^75,76^.

## Electronic supplementary material

Supplementary Information
