# Supplementary material for: Resurrecting Darwin’s Niata - anatomical, biomechanical, genetic, and morphometric studies of morphological novelty in cattle
Source: Sci Rep. 2018 Jun 14;8:9129. doi: 10.1038/s41598-018-27384-3 (PMC6002398; doi:10.1038/s41598-018-27384-3)
Supplement: Supplementary file 1 — Supplementary Information [file 41598_2018_27384_MOESM1_ESM.pdf]

Supplementary Information

Resurrecting Darwin's Niata - anatomical, biomechanical, genetic, and morphometric studies of morphological novelty in cattle

Kristof Veitschegger[1], Laura A. B. Wilson[2], Beatrice Nussberger[3], Glauco Camenisch[3], Lukas F. Keller[3,4], Stephen Wroe[2,5], and Marcelo R. Sánchez-Villagra*[1]

Table of Contents

Supplementary Text 1. Origin of the examined material

Supplementary Text 2. CT-scan of the Niata skull MNHN 1933-122

Supplementary Text 3. The basicranial angle

Supplementary Text 4. External suture obliteration pattern

Supplementary Text 5. The size and proportions of the Niata

Supplementary Text 6. Three dimensional morphometrics

Supplementary Text 7. Finite Element Analysis

References

Supplementary Figures 1-15

Suplementary Tables 1-14

Supplementary Text 1. Origin of the examined material

The cranial and postcranial material examined in this study is housed in following institutions: Institut für Haustierkunde (former; now Zoologisches Institut, Populationsgenetik), Christian-Albrechts-Universität zu Kiel, Germany (IfH), Naturhistorisches Museum Basel (NMB), Archäologisch Zoologische Sammlung des Naturhistorischen Museums Wien (NHM), Natural History Museum of Denmark (NHMD), Muséum National d'Histoire Naturelle Paris (MNHN), Museum für Naturkunde Berlin

(MfN), Museo de la Plata (MLP), Paleontological Institute and Museum University of Zurich (PIMUZ), Zentralmagazin Naturwissenschaftlicher Sammlungen – Museum für Haustierkunde „Julius Kühn“ – der Martin-Luther-Universität Halle-Wittenberg (ZNS Haustierkunde), and Zoological Museum University of Zurich (ZMUZH).

Supplementary Text 2. CT-scan of the Niata skull MNHN 1933-122

CT-scan of the Niata skull MNHN 1933-122 was carried out with a Phoenix v|tome|x L 240-180 at the AST-RX platform, MNHN, Paris, France (http://www.ums2700.mnhn.fr/ast-rx/acces). Following parameters were used: voltage 220 kV, current 300 mA. The scan resulted in 3053 images with a voxel size of 0.12357352 mm (exposure time 500 ms). Phoenix x-ray datos|x software was used to export a 16 bit TIFF image stack.

Supplementary Text 3. The basicranial angle

The basicranial angle or prebasial angle describes the relationship between the base of the viscerocranium and neurocranium. This angle is divided into three different types: lower as 180° are called klinorhynch, higher ones are called airorhynch, and an angle of 180° is called orthocranial. It was assumed that originally all skulls were orthocranial [1, 2]. During domestication, the basicranial angle can change substantially, as is the case for domesticated dogs were a range from 158° to 183° was described [3, 4]. This range is higher in cattle ranging from 158° to 193°. Incidentally, the data shows a gradual change from the aurochs to Niata. In pigs, it was suggested that aiorhynchie has an evolutionary advantage for feeding. The diet of wild boar and domesticated pigs differs and airorhynchie might facilitate a more optimal placement

of the lower jaw for the different diet humans provided [2]. We used 4 landmarks to obtain basicranial angles as shown in Supplementary Figure 2.

Supplementary Text 4. External suture obliteration pattern

We investigated the obliteration of 26 sutures externally to assess first if there are overall changes in the obliteration pattern between Niata cattle and other breeds and second if changes relating to chondrodysplasia can be detected (Supplementary Figure 1, Supplementary Table 1). Suture obliteration is correlated with changes in skull shape. Dogs with airorhynch skulls exhibit significant higher closure scores than their klinorhynch counterparts and both differ significantly from the wolf [3]. In cattle only the Niata was airorhynch all other cattle breeds were klinorhynch. Suture obliteration pattern was not different in Niata cattle. The rarity of juvenile Niata skulls, however, does limit hypotheses on similarity or differences in the overall obliteration scores. One suture was of greater informative value as early fusion of the basioccipital-basisphenoid suture is indicative for chondrodysplasia in cattle [5]. The skull ZMB_Mam_105902 can be dated to an individual age of 24-30 months based on tooth eruption [6] and exhibits an open suture at this bone contact area (Supplementary Figure 6).

Supplementary Text 5. The size and proportions of the Niata

Mutations causing chondrodysplasia are proven to be breed defining in dogs and cattle leading to shortened limbs [7, 8]. A famous example for this in cattle is the Dexter breed from Ireland [7, 9]. The height at the withers of Niata cattle was documented by Baldassare [10]. We used cranial and postcranial measurements to calculate the weight of the Niata to examine (Supplementary Table 3) possible delineations of

height to weight in the Niata, which could indicate shortening of limb bones [11-13]. We also compared the averaged limb length to the length of the vertebra column as additional measure for possible effects of chondrodysplasia on Niata. In his description, Darwin [14] also stated that frontlimb and hindlimb of the Niata might be disproportional. The relative proportions of the Niata were measured using several postcranial measurements (Supplementary Figure 3). We compared the proportional length of hindlimb and frontlimb as well as the ratio between limb and body length (Supplementary Figure 7, 8; Supplementary Table 9, 10). For the former, frontlimb length was defined as the sum of lateral lengths of humerus, radius, and metacarpal bone. Hindlimb length was defined as the sum of lateral lengths of femur, tibia, and metatarsal bone. All in all, we measured the limbs of 43 adult animals comprising 27 breeds for the comparison of limb length (Supplementary Table 9, 10). Body length was measured in seven mounted skeletons by measuring the distance between the anterior tip of the first cervical vertebra to the posterior tip of the sacrum in midline. This measurement was compared with the averaged limb length of the same skeletons (Supplementary Table 10). Here, frontlimb length was defined as distance between the ventral tip of the third lateral phalange to the dorsalmost point of the superior angulus of the scapula. The hindlimb length is defined as the distance between the ventral tip of the third phalange to the dorsalmost tip of the ilium (Supplementary Figure 3). The results show that the Niata was of comparable weight and height to other cattle breeds (Figure 3) as well as there was no disproportional change in frontlimb length to hindlimb length (Supplementary Figure 7). We did also not detect any obvious changes in the length of the appendicular skeleton compared to the axial skeleton (Supplementary Figure 8). Our results show that the Niata exhibits no chondrodysplastic changes to the postcranial skeleton typical for a chondrodysplastic condition [7, 8].

Supplementary Text 6. Three dimensional morphometrics

On the skull we digitized 53 landmarks (Supplementary Figure 4, Supplementary Table 5). Custom frame was used in MUS 6.0.1 to acquire landmarks on the dorsal and ventral part of the skull. Three reference landmarks (3, 4, and 30) were taken to set the frame and, after turning the skull, the same three reference landmarks were taken (Supplementary Figure 4). With this, the complete skull was landmarked. On the lower jaw we digitized 12 landmarks using the default setting for the frame (Supplementary Figure 10, Supplementary Table 12). The shape of the lower jaw was analyzed using the same procedure as described for the skulls in the main text.

Lower jaw analyses: the first three PC-axes of the PCA on lower jaws reflected 52 percent of the overall shape variation (Supplementary Figure 9b). PC-axis 1 reflected 30 percent, PC-axis 2 reflected 13 percent, and PC-axis 3 reflected 9 percent of the overall shape variation. The shape changes of the lower jaw in PC1-axis reflected the Niata condition. On the positive side of the axis the lower jaw was curved upwards. The second PC-axis was mostly defined by landmark 7 reflecting the difference in curvature of the angulus in cattle. The axis of PC3 showed the different extensions of the dental row. We used Procrustes distances to assess the difference among shapes by breed (Supplementary Table 11, Supplementary Table 13). The distances in skulls are extensively discussed in the main text. In the lower jaw the Niata were most different from *Bos primigenius* (PD: 0.18), Heck (PD: 0.15), and Walchshofer (PD: 0.15). These distances were shorter than observed in the skulls. However, the closest breeds to the Niata in lower jaw shape were further removed than in skulls. These closest breeds were Vogtlaender (PD: 0.09), Normand (PD: 0.09), and Tuxer (PD: 0.10).

Supplementary Text 7. Finite Element Analysis

Methods for muscle forces scaling, for Finite Element Model (FEM) assembly and for comparison of biomechanical performance (see references in main text)

Muscle forces were scaled according to body mass for each specimen [43]. When a body is scaled geometrically by a factor of k in all dimensions, the volume of the body scales by k3 whereas the muscle cross-sectional area of the body scales by k2; this can be expressed in the following, two-thirds power scaling equation:

MFtarget / MFref = (BMtarget/BMref)^2/3

Where MFtarget is the muscle force of the target specimen (to be calculated), MFref is the muscle force of the reference specimen, BMtarget is the body mass of the target specimen, and BMref is the body mass of the reference specimen [44, 45]. We used the Niata MLP 1126 model as MFref to estimate MFtarget for the Simmentaler and Zebu models, using the above equation.

Finite Element Model (FEM) assembly: Three-dimensional (3D) surface meshes were created from DICOM image stacks using Mimics (Materialize, Version 18.0), and volume meshing was performed in 3-Matic (Materialize, Version 9.0). Volume meshes were imported as Nastran (NAS) files into Strand 7 v. 2.4.5 (Strand 7, Pty Ltd, Sydney, NSW) for Finite Element Model (FEM) assembly (Supplementary Figure 5). Each model comprised 1.5-1.8 million 4-noded tetrahedral (tet-4) 'brick' elements: Niata MLP 1126 - 1,781,890 bricks; Simmentaler 17765 – 1,552,228 bricks; Zebu 17767 – 1,594,008 bricks. Following previous protocols for comparative Finite Element Analysis (FEA), all FEMs were homogeneous and tet-4 elements were as-

signed a single material property for cortical bone with a Young's modulus (E) of 13.7GPa and a Poisson's ratio (v) of 0.4 [44, 46]. Muscle architecture was modelled in 3D using multiple pre-tensioned trusses (axial-loaded beam elements), to simulate the basic geometry of muscle fibers (total 70 trusses per model). Each muscle group was represented by the same number of trusses in each model, and muscle forces were distributed between the trusses relative to muscle proportion values, as calculated above. For each muscle beam, a network of beams was tessellated on the surface of the model around the site of attachment to reduce the potential for stress artefacts associated with single node loadings.

Boundary conditions and loading cases for FEMs: Two intrinsic (bite transmitted) loading cases were simulated to mimic feeding, reflecting bites undertaken with maximal bite force using the skull musculature. These were: 1) a bilateral anterior bite at M1, and 2) a unilateral molar bite at M2. For both loading cases, models were restrained at the occipital condyle and an axis of rotation was created around the temporomandibular joint (TMJ). A rigid link spanning the foramen magnum was created and restrained in the global coordinate system (x, y, z restraint for translation and rotation) to prevent free motion of the model in virtual space. Restraints were also added at the mid-point, defined as the mid-length, of the upper M1, for each tooth row for the bilateral bite, and for the right hand side tooth row only for the unilateral bite. Restraints simulate occlusal and mandibular contacts and produce reaction forces at the point of restraint under muscle pull in an inferior line of action (see [47, 48] for similar protocol). For the bilateral bite, the TMJ was restrained in all directions, and the bite points on the tooth row were constrained in the vertical direction. For the unilateral bite, the working-side TMJ was constrained in all directions, and the balancing-side TMJ was constrained in the vertical and antero-posterior directions. To spread the forces generated at the restrained single nodes, and to prevent artefacts

in the distribution and magnitude of stress [49], networks of beams were tessellated around each fixed node.

Comparison of biomechanical performance: FEMs were solved in Strand 7 (version 2.4.5) using the direct sparse linear static solve scheme, and the AMD node ordering algorithm option. Prior to solving each model, the mandible was removed due to the inclusion of metal fixations in the Niata MLP 1126 specimen. Von Mises (VM) stress data were used to compare the structural integrity of the FEMs under the loading cases. Bone fails under a ductile model of fracture [50], and VM stress is the metric used to assess the yielding of ductile materials [51].

Relative mechanical performance was assessed using contour plots of VM stress distributions, generated in Strand 7 (version 2.4.5). To assess differences in stress magnitude and distribution between models, VM brick stress values were extracted at equidistant points along the mid-saggital plane of the cranium and along the margin of the tooth row for each model. At each equidistant point, VM stress values for five bricks surrounding the selected node were averaged. On each model, the selected node identity (ID) was recorded and the same node was chosen for each loading case. For mid-saggital sampling, equidistant points were selected along a line from the most anterior part of the nasal bone (point 1) to the most posterior part of the parietal (point 10). For the margin of the tooth row, equidistant points were selected along a line from the most anterior (point 1) to most posterior part (point 10) of the tooth row.

Reaction forces, recorded at the bite points and TMJ for each loading case, were extracted from each of the solved FEMs. Strain metrics were extracted at the TMJ on both the working and balancing sides of the model. These comprised von Mises strain (distortional strain or non-isometric strain), maximum principal strain (tension) and minimum principal strain (compression). Strain mode was calculated as the ab-

solute value of maximum principal strain divided by minimum principal strain, to provide an indication of whether compression or tension was dominant at the TMJs (see [48]).

References

1. Hofer H. 1952 Der Gestaltwandel des Schädels der Säugetiere und Vögel, mit besonderer Berücksichtigung der Knickungstypen und der Schädelbasis. *Verh. Anat. Ges.* **50**, 102-113.

2. Thenius E. 1970 Zum Problem der Airorhynchie des Säugetierschädels – Ein Deutungsversuch. **185**, 159-172.

3. Geiger M, Haussman S. 2016 Cranial suture closure in domestic dog breeds and its relationships to skull morphology. *Anat. Rec. (Hoboken)* **299**, 412-420. (doi:10.1002/ar.23313)

4. Nussbaumer M. 1982 On the variability of dorso-basal curvatures in skulls of domestic dogs. *Zool. Anz.* **209**, 1-32.

5. McKinley LJ, Steele WT, Hage TJ, Gregory PW. 1957 Premature closure of the spheno-occipital synchondrosis in the horned Hereford dwarf of the "short-headed" variety. *Am. J. Anat.* **100**, 269-287.

6. Habermehl K-H. 1975 *Die Altersbestimmung bei Haus- und Labortieren*. Berlin und Hamburg, Germany: Paul Parey.

7. Cavanagh JAL, Tammen I, Windsor PA, Bateman JF, Savarirayan R, Nicholas FW, Raadsma HW. 2007 Bulldog dwarfism in Dexter cattle is caused by mutations in ACAN. *Mamm. Genome* **18**, 808-814. (doi:10.1007/s00335-007-9066-9)

8. Parker HG, VonHoldt BM, Quignon P, Margulies EH, Shao S, Mosher DS, Spady TC, Elkahloun A, Cargill M, Jones PG, et al. 2009 An expressed Fgf4

retrogene is associated with breed-defining chondrodysplasia in domestic dogs. *Science* **325**, 995-998. (doi:10.1126/science.1173275)

9. Felius M. 1995 *Cattle breeds: an encyclopedia*. Doetinchem, Netherlands: Misset.

10. Baldassarre S. 1906 La Zootecnia nella Repubblica Argentina. *Atti del Reale Istituto d'Incoraggiamento alle Scienze Naturali di Napoli* **6**, 359-380.

11. Scott KM. 1983 Prediction of body weight of fossil Artiodactyla. *Zool. J. Linn. Soc.* **77**, 199-215. (doi:10.1111/j.1096-3642.1983.tb00098.x)

12. Anderson JF, Hall-Martin A, Russell DA. 1985 Long-bone circumference and weight in mammals, birds and dinosaurs. *J. Zool.* **207**, 53-61. (doi:10.1111/j.1469-7998.1985.tb04915.x)

13. Janis CM. 1990 Correlation of cranial and dental variables with body size in ungulates and macropodoids. In: Damuth JD, MacFadden BJ, editors. *Body Size in Mammalian Paleobiology: Estimation and Biological Implications*. Cambridge: Cambridge University Press.

14. Darwin C. 1845 *Journal of researches into the geology and natural history of the various countries visited by H.M.S. Beagle round the world, under the Command of Capt. Fitz Roy, R.N*. London, UK: John Murray.

15. Thomason JJ. 1991 Cranial strength in relation to estimated biting forces in some mammals. *Can. J. Zool.* **69**, 2326-2333. (doi:10.1139/z91-327)

16. Wroe S, Chamoli U, Parr WCH, Clausen P, Ridgely R, Witmer L. 2013 Comparative biomechanical modeling of metatherian and placental saber-tooths: a different kind of bite for an extreme pouched predator. *Plos One* **8**, e66888. (doi:10.1371/journal.pone.0066888)

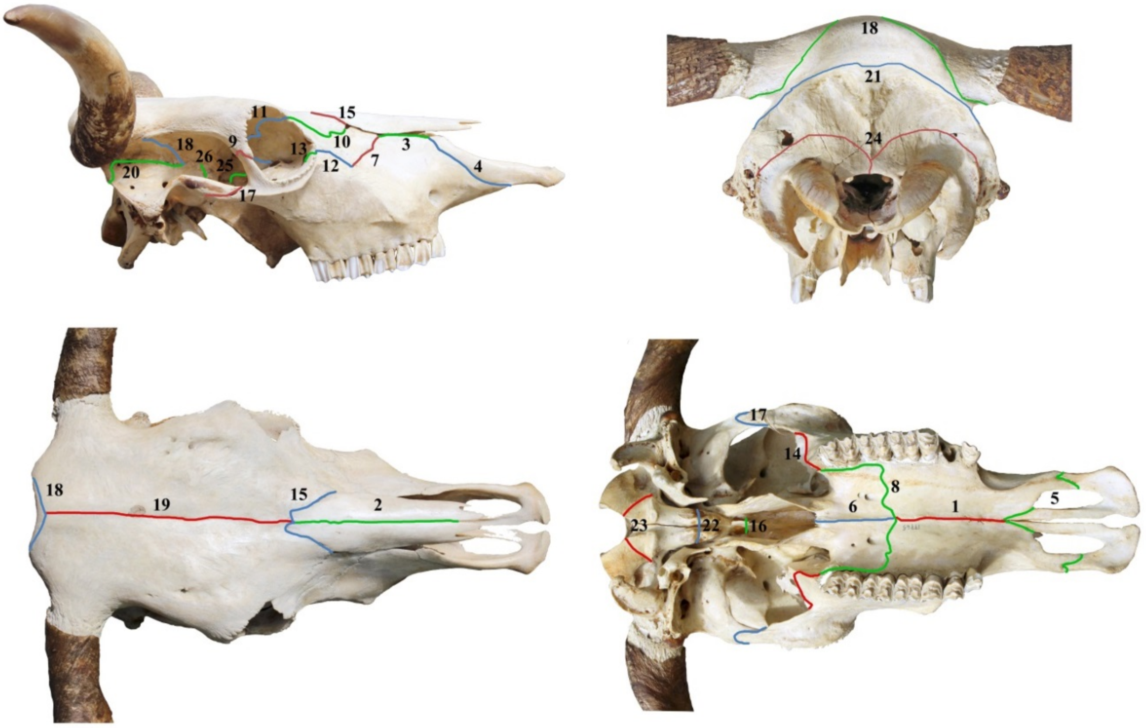

Supplementary Figure 1: Sutures used to assess the external suture obliteration pattern during growth of Niata and other cattle. Corresponding suture description to numbers are found in Supplementary Table 3.

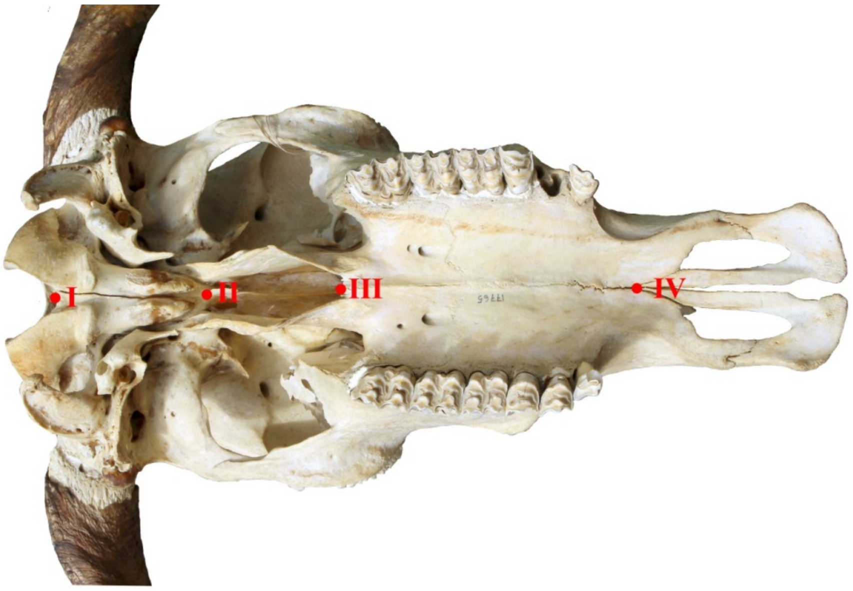

Supplementary Figure 2: Landmarks used to measure the basicranial angle. I: ventral-medial edge of foramen magnum, II: suture between presphenoid and vomer in midline, III: posterior-medial tip of the horizontal plate of the palatine bone, IV: ventral suture between premaxillary and maxillary bone in midline.

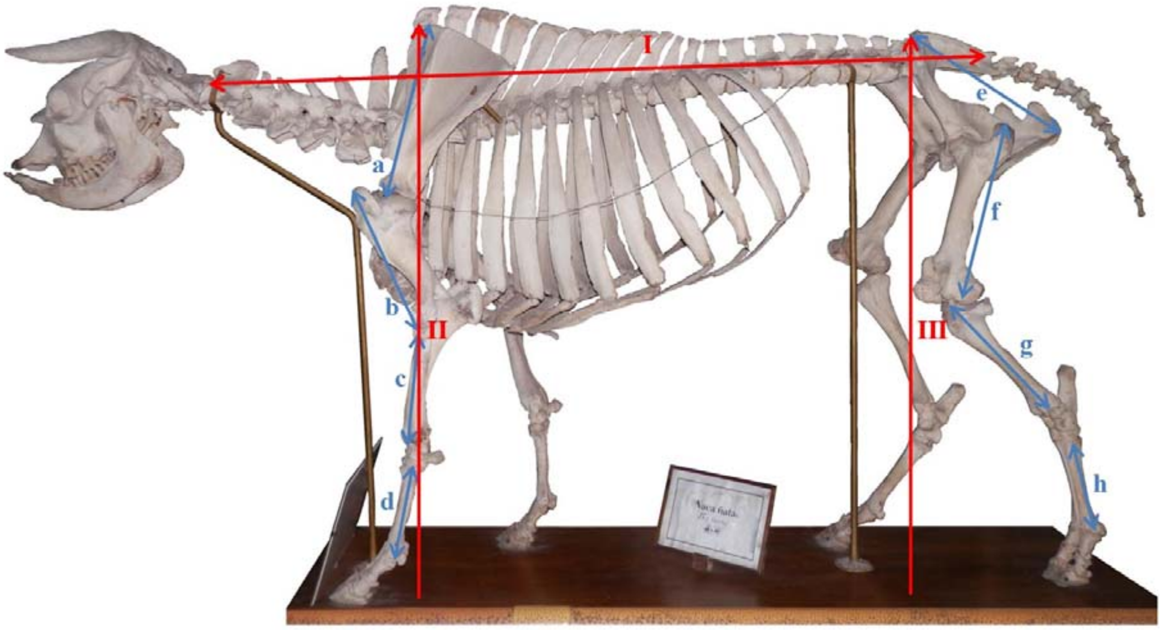

Supplementary Figure 3: Picture of the mounted skeleton MLP 1126. Indicated are the postcranial measurements used to compare height and length of the Niata as well as front limb and hindlimb length. I: cervical-sacrum length; II: length frontlimb; length hindlimb; a: anterior length of scapula; b: lateral length of humerus; c: lateral length of radius; d: lateral length of the metacarpal bone; e: length of pelvis; f: lateral length of femur; g: lateral length of tibia; h: lateral length of the metatarsal bone.

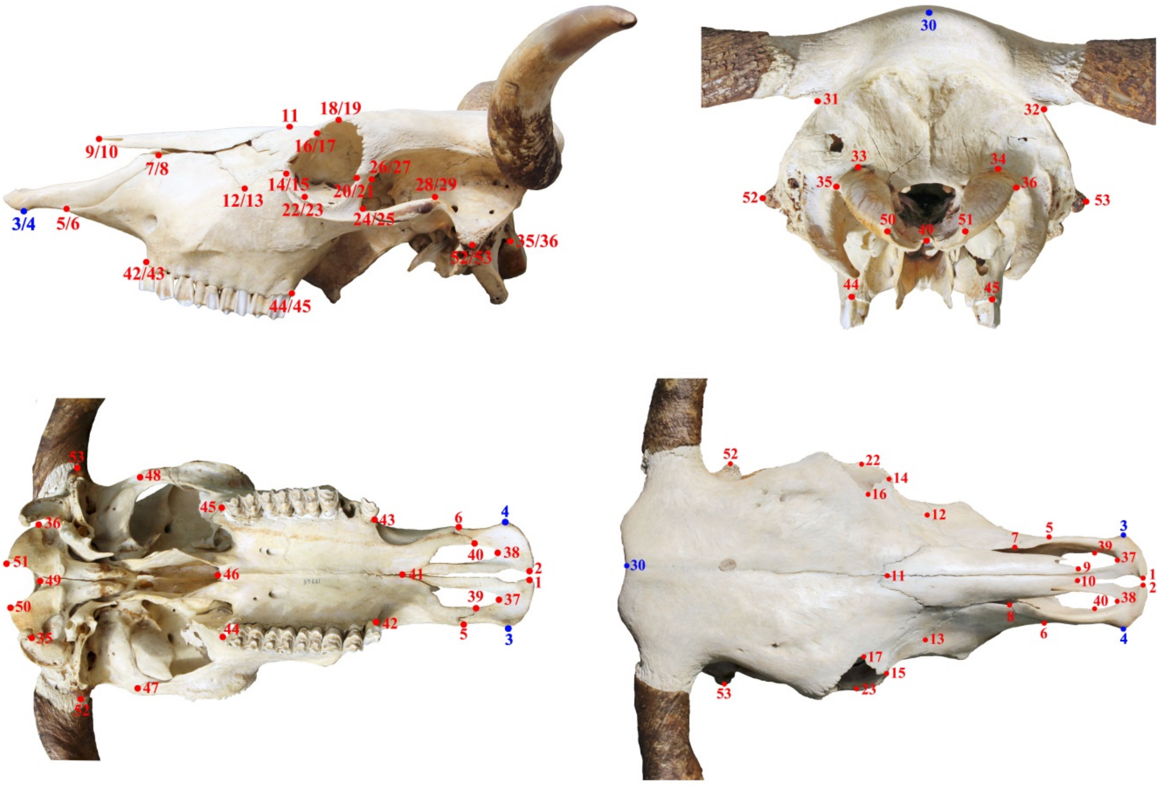

Supplementary Figure 4: Landmarks on the skull used in this study. Indicated in blue are the land-marks used for custom frame to rotate the skull.

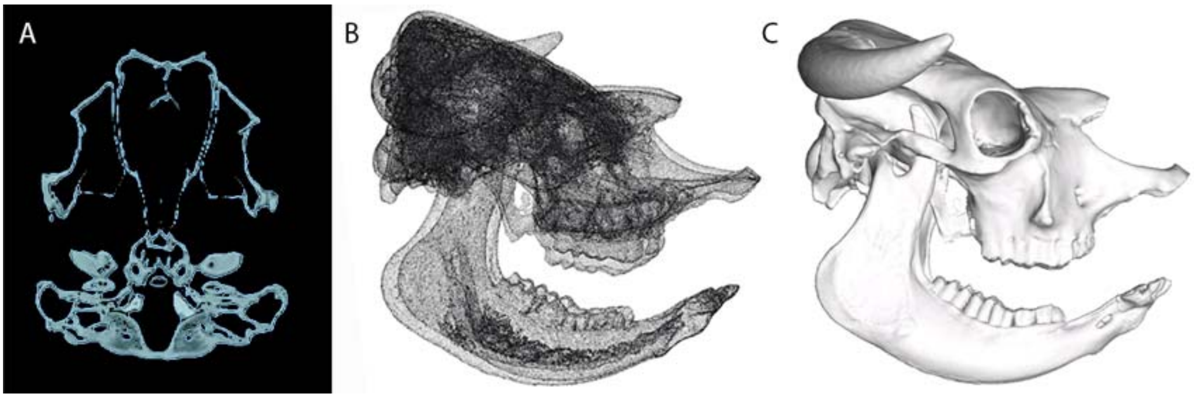

Supplementary Figure 5: Creating virtual models for Finite Element Analysis (FEA): (A) thresholding of computed tomography (CT) image slices, seen in axial view; (B) generation of a volume mesh following remeshing of surface mesh using controlled geometric error and triangle edge length values; (C) assembly of a Finite Element Model (FEM) in Strand 7. Model shown is for the Niata breed, specimen MLP 1126.

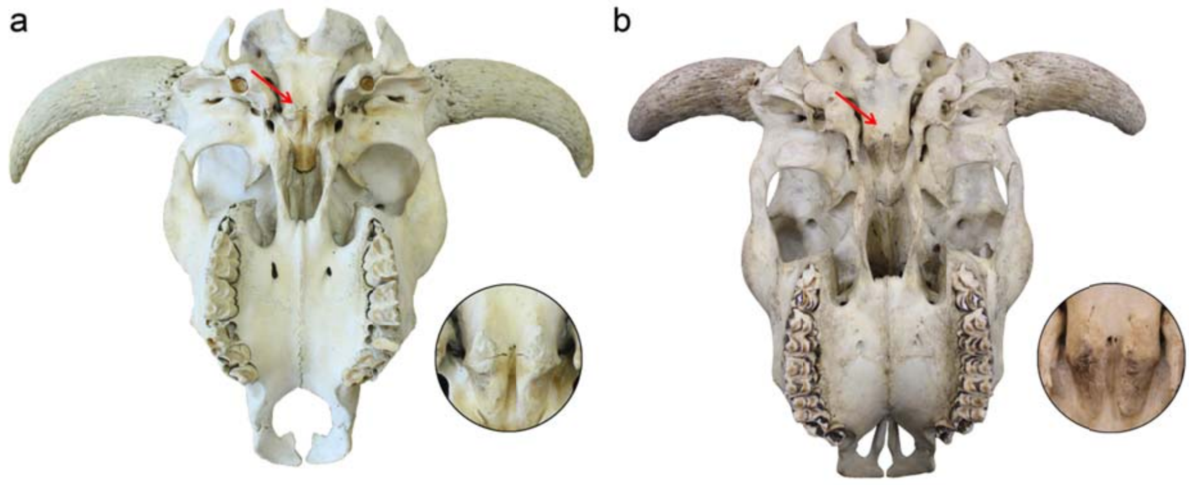

Supplementary Figure 6: Ventral view of Niata skull ZMB_Mam_105902 and NHMD-ZMK-MK-1109 (by K.M. Gregersen, NHMD). Note the open suture between basioccipital and basisphenoid bone in a and the closed one in b. The eruption of the premolars as well as third molar indicating and age of 24 – 30 months[6]

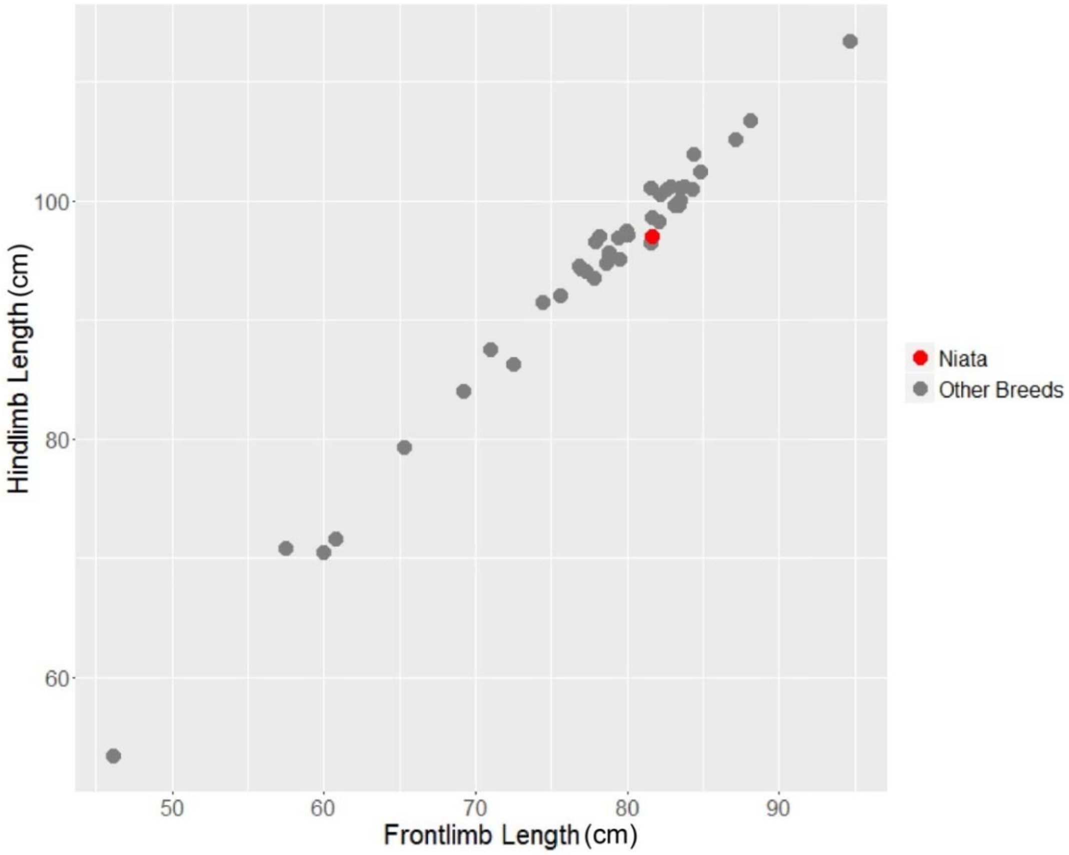

Supplementary Figure 7: Results of the comparison between frontlimb and hindlimb length among breeds.

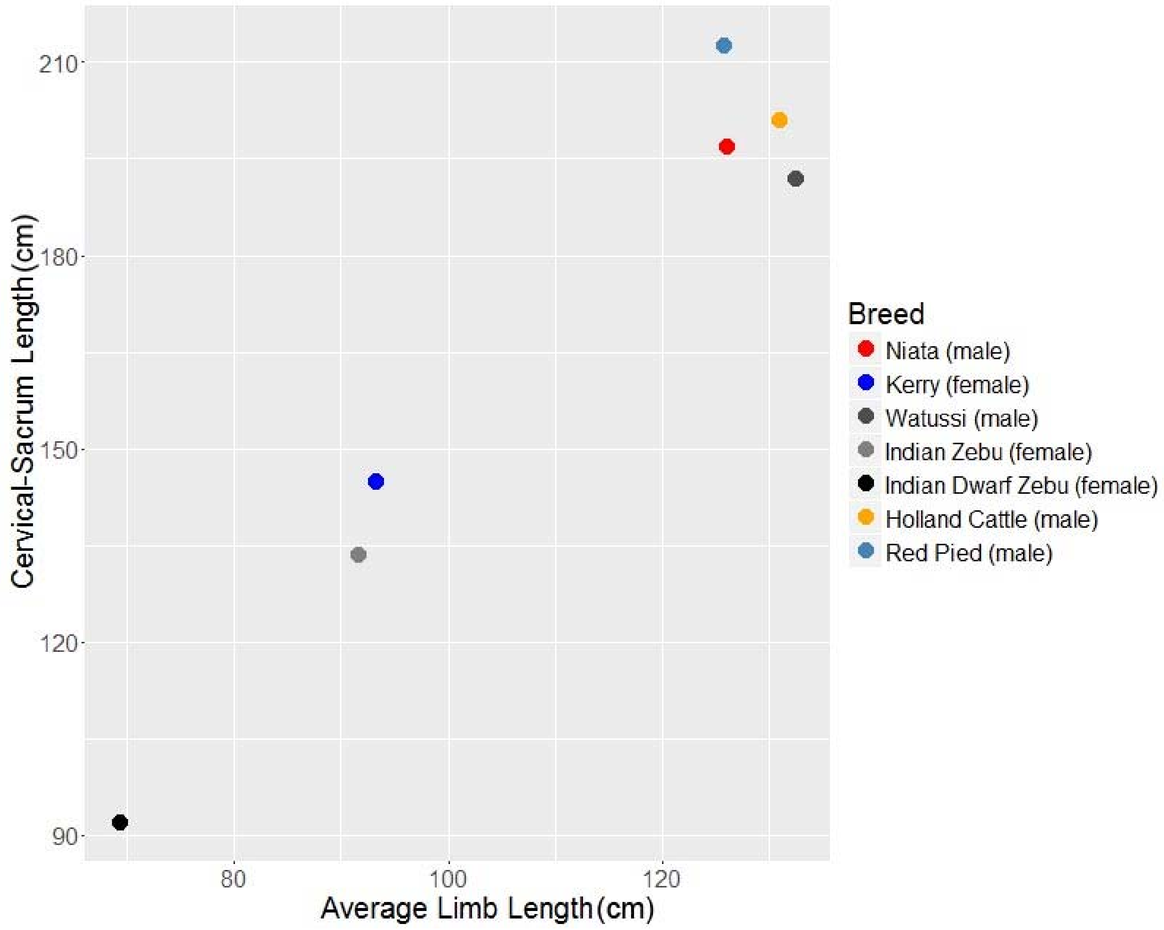

Supplementary Figure 8: Results of the comparison between axial and appendicular skeleton among breeds.

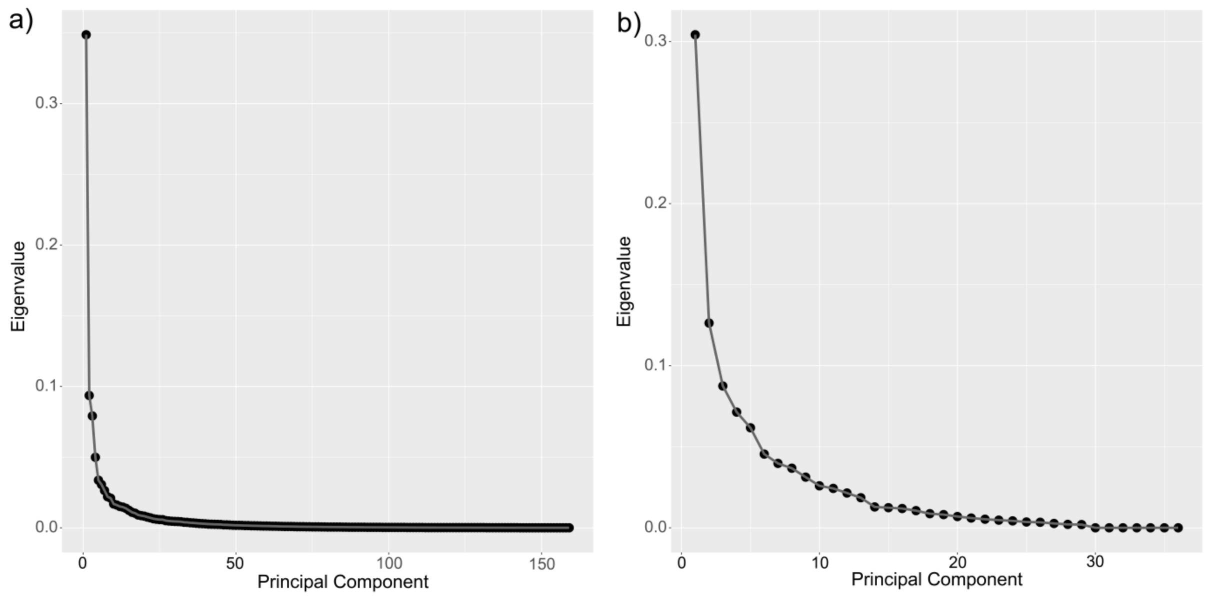

Supplementary Figure 9: Eigenvalues of the PC-axes from PCA on the skulls (a) and lower jaws (b) of different cattle breeds.

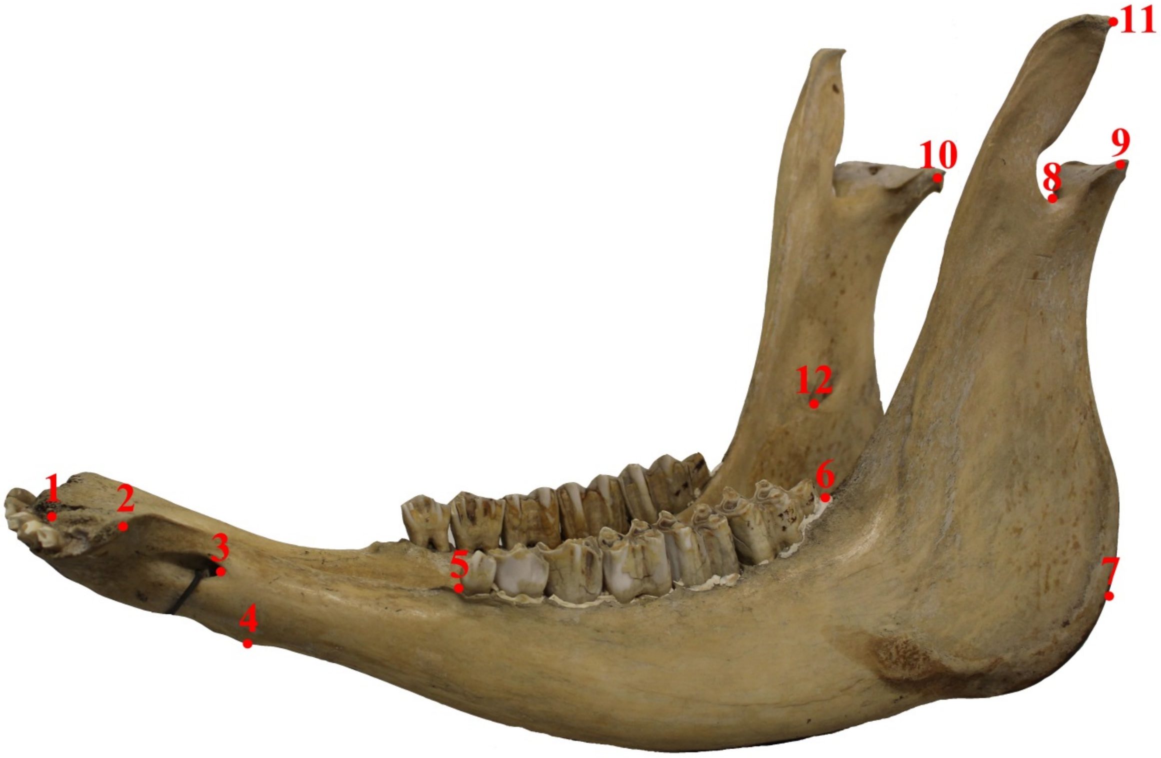

Supplementary Figure 10: Landmarks on the lower jaw used in this study.

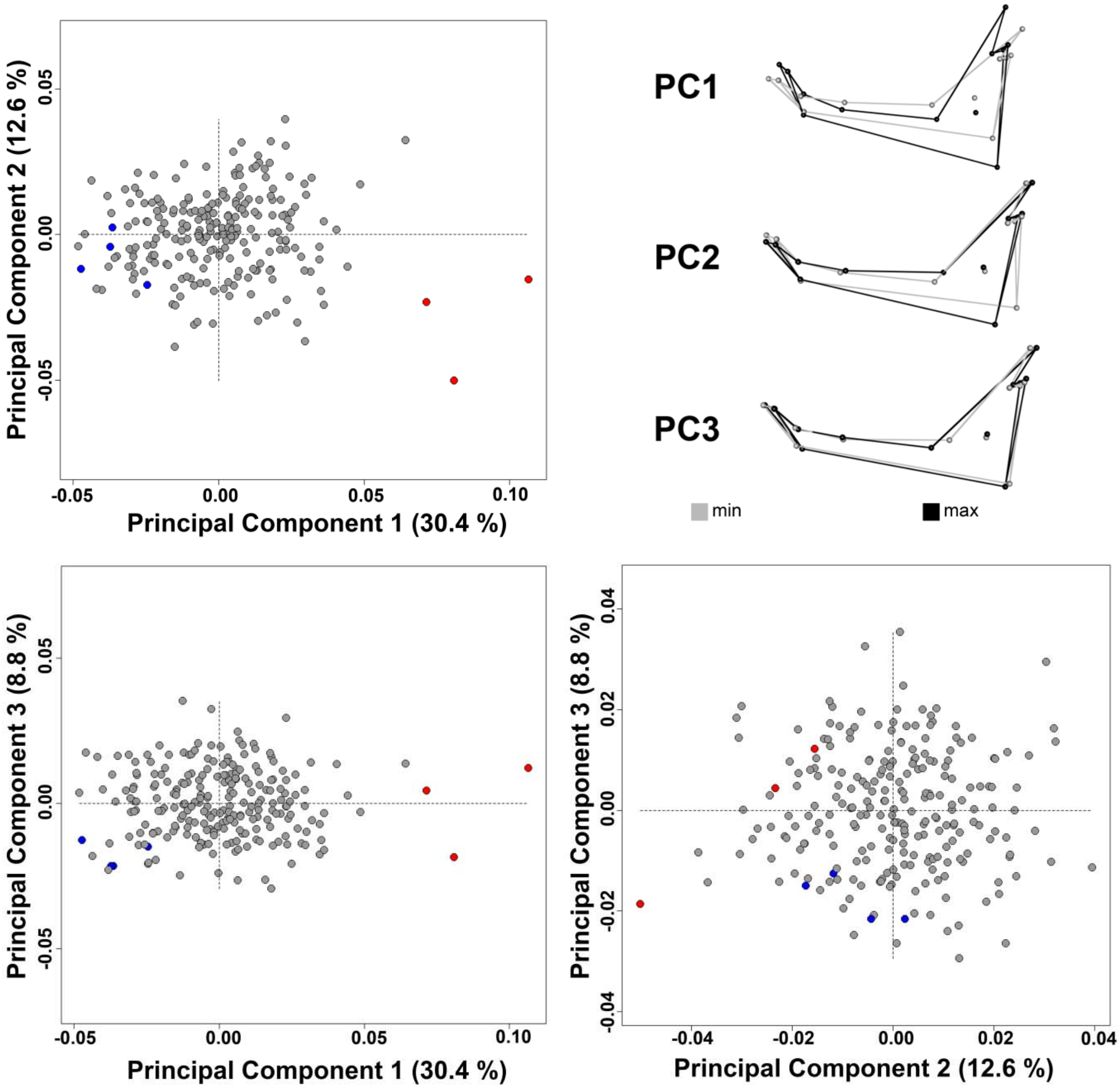

Supplementary Figure 11: Principal Component Analysis of the lower jaw shapes of different cattle breeds. Top left, bottom left and bottom right are the comparisons between PC1, PC2, and PC3. Top right are the associated shape changes.

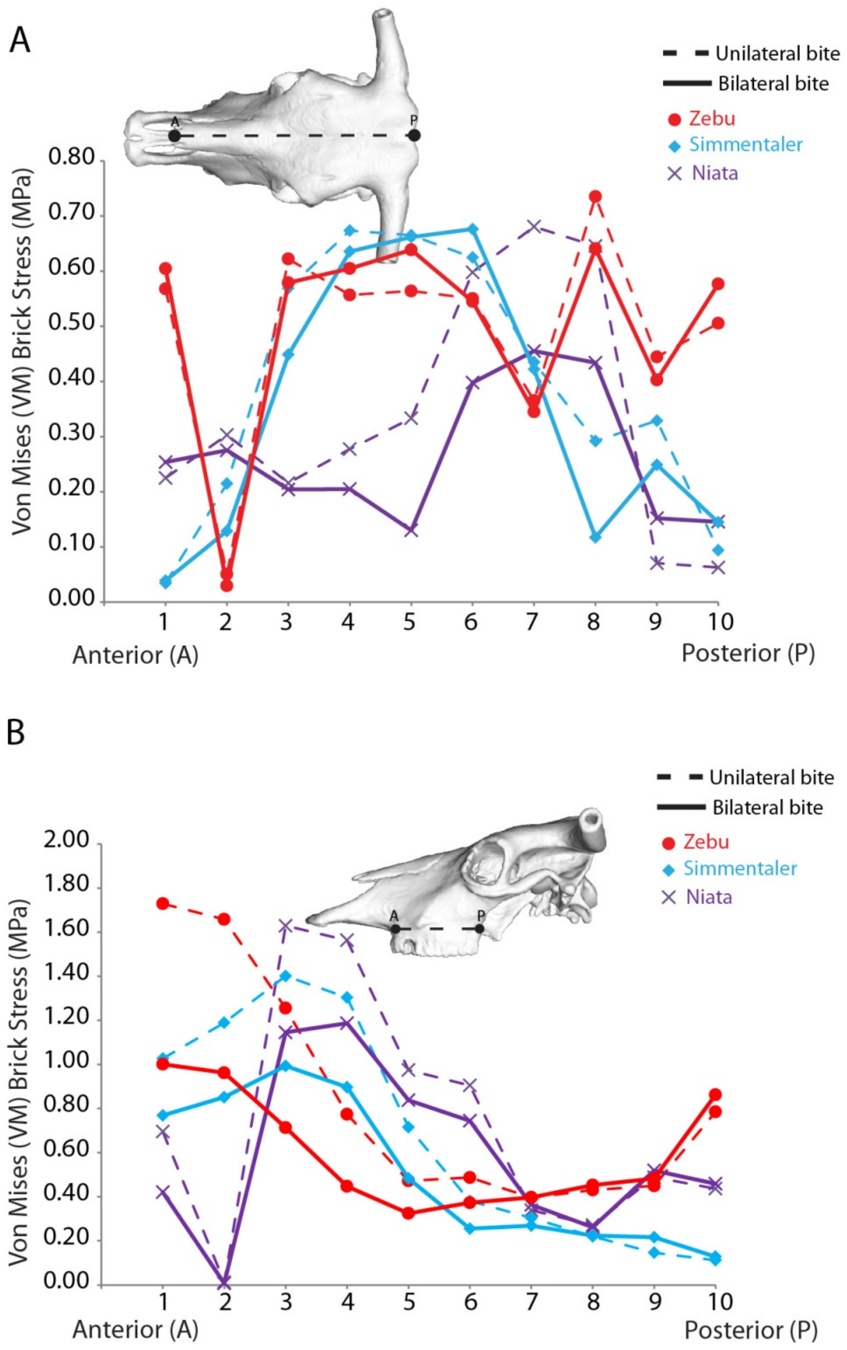

Supplementary Figure 12: Von Mises stress plots: VM stress values were extracted at 10 equidistant points from anterior to posterior along the mid-saggital plane (A) and margin of the tooth row (B) for each model under loading conditions simulating an anterior bilateral bite (solid line) and an anterior unilateral bite (dashed line).

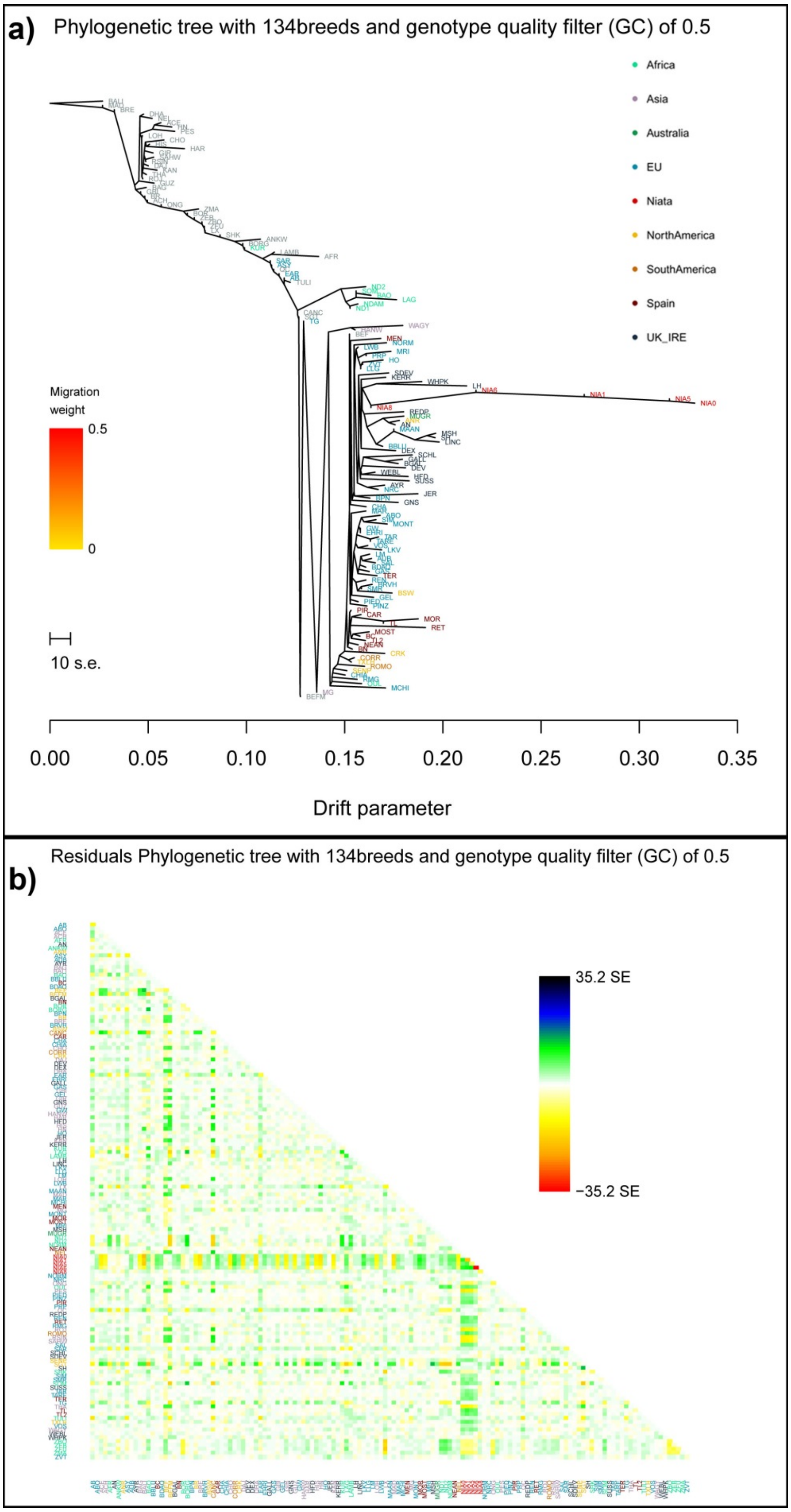

Supplementary Figure 13: a) Phylogenetic tree with 134 breeds and 5 Niata samples of GC ≥ 0,5. b) Residuals.

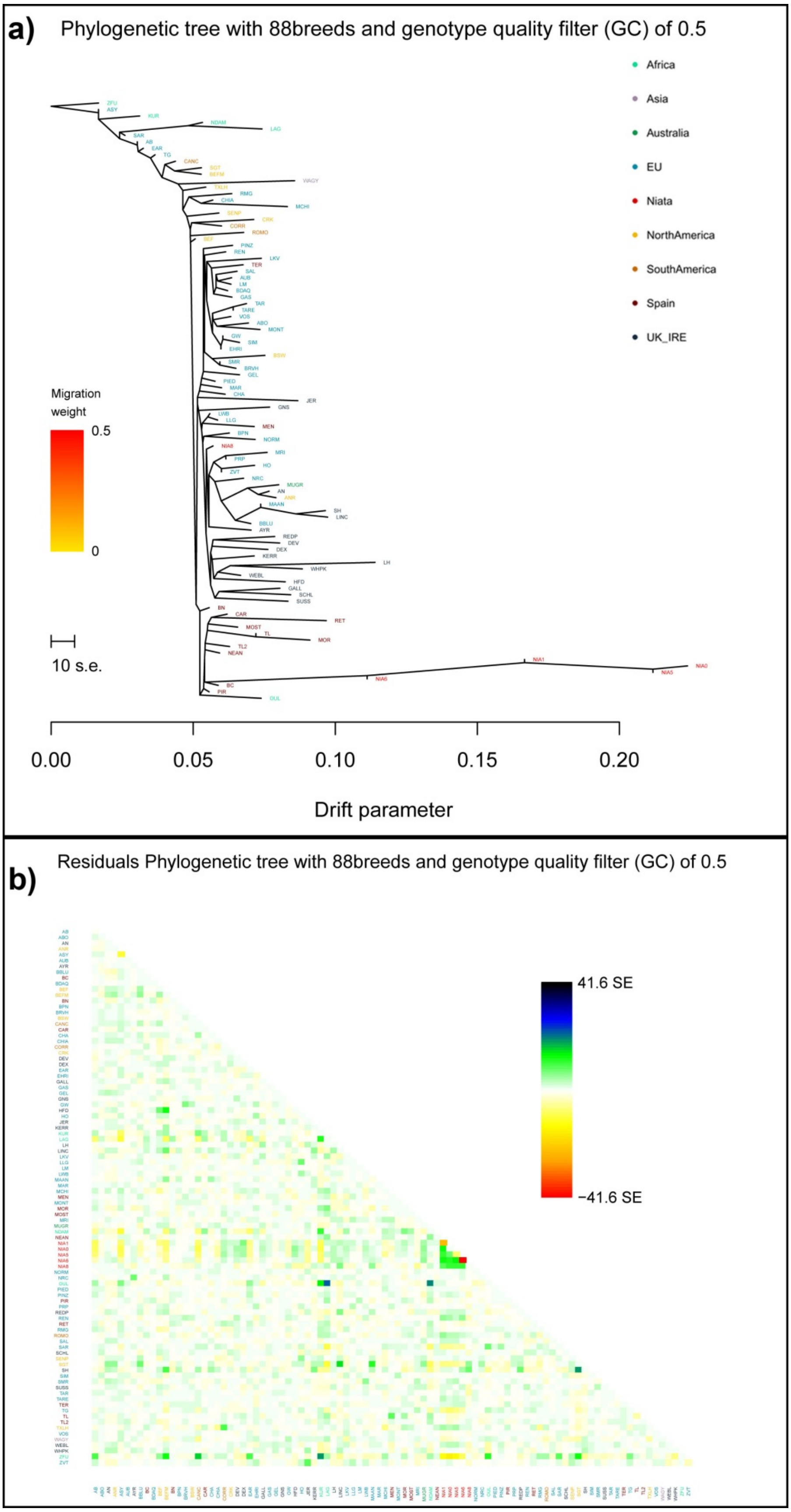

Supplementary Figure 14: a) Phylogenetic tree with 88 breeds and 5 Niata samples of GC ≥ 0,5. b) Residuals.

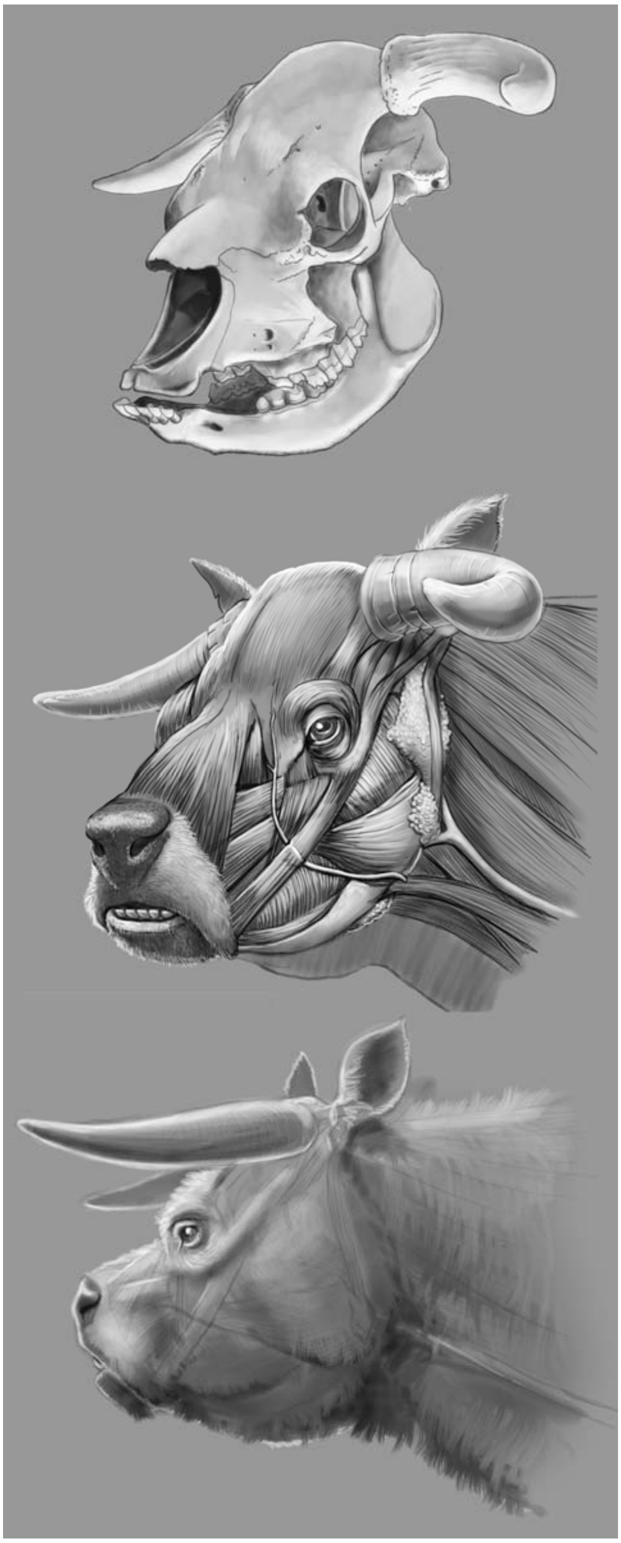

Supplementary Figure 15: Skull and reconstruction of cranial muscles and external head morphology of the Niata based on the skull Museo de La Plata MLP 1126. Artwork by Jorge González.

Supplementary Table 1: External suture obliteration in Niata as well as other cattle breeds and corresponding relative suture closure score.

	Breed	Danish Red	Danish Red	x	x
	Inventory Number	SNM-KU MK 496	SNM-KU MK 497	SNM-KU Nov. 1935	SNM-KU 14/1-1910
	Age in months	0.23	2	8	9
	Sex	Male	Male	Male	Male
1	Intermaxillary	0	0	0	0
2	Internasal	0	0	0	0
3	Nasal-Maxillary	0	0	0	0
4	Premaxillary-Maxillary (f)	0	0	0	0
5	Premaxillary-Maxillary (v)	0	0	0	0
6	Interpalatine	0	0	0	0
7	Maxillary-Lacrimal (f)	0	0	0	0
8	Maxillary-Palatine (v)	0	0	0	0
9	Jugal-Frontal	0	0	0	0
10	Lacrimal-Frontal (f)	0	0	0	0
11	Lacrimal-Frontal (o)	0	0	0	0
12	Lacrimal-Jugal (f)	0	0	0	0
13	Lacrimal-Jugal (o)	0	0	0	0
14	Maxillary-Lacrimal (o)	0	0	0	0
15	Nasal-Frontal	0	0	0	0
16	Basisphenoid - Presphenoid	0	0	0	0
17	Jugal-Squamosal	0	0	0	0
18	Frontal-Parietal	0	0	1	0
19	Interfrontal	0	0	0	0
20	Parietal-Squamosal	0	0	0	0
21	Supraoccipital-Parietal	0	1	2	2
22	Basisphenoid-Basioccipital	0	0	0	0
23	Exoccipital-Basioccipital	0	0	0	1
24	Exoccipital-Supraoccipital	0	0	0	0
25	Orbitosphenoid-Frontal	0	0	0	0
26	Alisphenoid-Squamosal	0	0	0	0
	Suture Closure Score	0.00	0.04	0.12	0.12

	Breed	German Black Pied	German Black Pied	German Black Pied	German Black Pied
	Inventory Number	IfH 11316	IfH 11318	IfH 11319	IfH 11315
	Age in months	10	10	10	10
	Sex	Female	Female	Female	Female
1	Intermaxillary	0	0	0	0
2	Internasal	0	0	0	0
3	Nasal-Maxillary	0	0	0	0
4	Premaxillary-Maxillary (f)	0	0	0	0
5	Premaxillary-Maxillary (v)	0	0	0	0
6	Interpalatine	0	0	0	0
7	Maxillary-Lacrimal (f)	0	0	0	0
8	Maxillary-Palatine (v)	0	0	0	0
9	Jugal-Frontal	0	0	0	0
10	Lacrimal-Frontal (f)	0	0	0	0
11	Lacrimal-Frontal (o)	0	0	0	0
12	Lacrimal-Jugal (f)	0	0	0	0
13	Lacrimal-Jugal (o)	0	0	0	0
14	Maxillary-Lacrimal (o)	0	0	0	0
15	Nasal-Frontal	0	0	0	0
16	Basisphenoid - Presphenoid	0	0	0	0
17	Jugal-Squamosal	0	0	0	0
18	Frontal-Parietal	0	0	0	0
19	Interfrontal	0	0	0	0
20	Parietal-Squamosal	0	0	0	0
21	Supraoccipital-Parietal	2	2	2	2
22	Basisphenoid-Basioccipital	0	0	0	0
23	Exoccipital-Basioccipital	0	0	0	0
24	Exoccipital-Supraoccipital	0	0	0	0
25	Orbitosphenoid-Frontal	0	0	0	0
26	Alisphenoid-Squamosal	0	0	0	0
	Suture Closure Score	0.08	0.08	0.08	0.08
	Breed	German Black Pied	x	German Black Pied	German Black Pied
	Inventory Number	IfH 11317	SNM-KU MK 168	IfH 11361	IfH 11283

	Age in months	10	12	12.5	15
	Sex	Female	Male	Female	Female
1	Intermaxillary	0	0	0	0
2	Internasal	0	0	0	0
3	Nasal-Maxillary	0	0	0	0
4	Premaxillary-Maxillary (f)	0	0	0	0
5	Premaxillary-Maxillary (v)	0	0	0	0
6	Interpalatine	0	0	0	0
7	Maxillary-Lacrimal (f)	0	0	0	0
8	Maxillary-Palatine (v)	0	0	0	0
9	Jugal-Frontal	0	0	0	0
10	Lacrimal-Frontal (f)	0	0	0	0
11	Lacrimal-Frontal (o)	0	0	0	0
12	Lacrimal-Jugal (f)	0	0	0	0
13	Lacrimal-Jugal (o)	0	0	0	0
14	Maxillary-Lacrimal (o)	0	0	0	0
15	Nasal-Frontal	0	0	0	0
16	Basisphenoid - Presphenoid	0	0	0	0
17	Jugal-Squamosal	0	0	0	0
18	Frontal-Parietal	0	1	0	0
19	Interfrontal	0	0	0	0
20	Parietal-Squamosal	0	0	0	0
21	Supraoccipital-Parietal	2	2	2	2
22	Basisphenoid-Basioccipital	0	0	0	0
23	Exoccipital-Basioccipital	1	2	0	2
24	Exoccipital-Supraoccipital	0	0	0	0
25	Orbitosphenoid-Frontal	0	0	0	0
26	Alisphenoid-Squamosal	0	0	0	0
	Suture Closure Score	0.12	0.19	0.08	0.15
	Breed	x	x	x	x
	Inventory Number	SNM-KU MK 171	SNM-KU MK 175	IfH 11320	SNM-KU MK 164
	Age in months	15	15	15	16
	Sex	Male	Male	Female	Male

1	Intermaxillary	0	0	0	0
2	Internasal	0	0	0	0
3	Nasal-Maxillary	0	0	0	0
4	Premaxillary-Maxillary (f)	0	0	0	0
5	Premaxillary-Maxillary (v)	0	0	0	0
6	Interpalatine	0	0	0	0
7	Maxillary-Lacrimal (f)	0	0	0	0
8	Maxillary-Palatine (v)	0	0	0	0
9	Jugal-Frontal	0	0	0	0
10	Lacrimal-Frontal (f)	0	0	0	0
11	Lacrimal-Frontal (o)	0	0	0	0
12	Lacrimal-Jugal (f)	0	0	0	0
13	Lacrimal-Jugal (o)	0	0	0	0
14	Maxillary-Lacrimal (o)	0	0	0	0
15	Nasal-Frontal	0	0	0	0
16	Basisphenoid - Presphenoid	0	0	0	0
17	Jugal-Squamosal	0	0	0	0
18	Frontal-Parietal	0	1	0	1
19	Interfrontal	0	0	0	0
20	Parietal-Squamosal	0	0	0	0
21	Supraoccipital-Parietal	2	2	2	2
22	Basisphenoid-Basioccipital	0	0	0	0
23	Exoccipital-Basioccipital	1	2	2	2
24	Exoccipital-Supraoccipital	0	0	0	0
25	Orbitosphenoid-Frontal	0	0	0	0
26	Alisphenoid-Squamosal	0	0	0	0
	Suture Closure Score	0.12	0.19	0.15	0.19
	Breed	x	Hinterwälder	x	Scottish Highland
	Inventory Number	IfH 11363	IfH 4624	SNM-KU MK 162	SNM-KU MK 571
	Age in months	16.5	16.5	21	24
	Sex	Female	Male	Male	Male
1	Intermaxillary	0	0	0	0
2	Internasal	0	0	0	0

3	Nasal-Maxillary	0	0	0	0
4	Premaxillary-Maxillary (f)	0	0	0	0
5	Premaxillary-Maxillary (v)	0	0	0	0
6	Interpalatine	0	0	0	0
7	Maxillary-Lacrimal (f)	0	0	0	0
8	Maxillary-Palatine (v)	0	0	0	0
9	Jugal-Frontal	0	0	0	0
10	Lacrimal-Frontal (f)	0	0	0	0
11	Lacrimal-Frontal (o)	0	0	0	0
12	Lacrimal-Jugal (f)	0	0	0	0
13	Lacrimal-Jugal (o)	0	0	0	0
14	Maxillary-Lacrimal (o)	0	0	0	0
15	Nasal-Frontal	0	0	0	0
16	Basisphenoid - Presphenoid	0	0	0	0
17	Jugal-Squamosal	0	0	0	0
18	Frontal-Parietal	0	1	1	1
19	Interfrontal	0	0	0	1
20	Parietal-Squamosal	0	0	0	0
21	Supraoccipital-Parietal	2	2	2	2
22	Basisphenoid-Basioccipital	0	0	0	1
23	Exoccipital-Basioccipital	2	2	2	2
24	Exoccipital-Supraoccipital	0	0	0	1
25	Orbitosphenoid-Frontal	0	0	0	1
26	Alisphenoid-Squamosal	0	0	0	0
	Suture Closure Score	0.15	0.19	0.19	0.35
	Breed	Niata	German Black Pied	x	Longhorn
	Inventory Number	ZMB_Mam_105902	IfH 11522	IfH 4185	ZNS Haustierkunde B lgh 4
	Age in months	24	27.5	28	28
	Sex	x	x	Female	Male
1	Intermaxillary	0	0	0	0
2	Internasal	0	0	0	0
3	Nasal-Maxillary	0	0	0	0

4	Premaxillary-Maxillary (f)	0	0	0	0
5	Premaxillary-Maxillary (v)	0	0	0	0
6	Interpalatine	0	0	0	0
7	Maxillary-Lacrimal (f)	0	0	0	0
8	Maxillary-Palatine (v)	0	0	0	0
9	Jugal-Frontal	0	0	0	0
10	Lacrimal-Frontal (f)	0	0	0	0
11	Lacrimal-Frontal (o)	0	0	0	0
12	Lacrimal-Jugal (f)	0	0	0	0
13	Lacrimal-Jugal (o)	0	0	0	0
14	Maxillary-Lacrimal (o)	0	0	0	0
15	Nasal-Frontal	0	0	0	0
16	Basisphenoid - Presphenoid	0	0	0	0
17	Jugal-Squamosal	0	0	0	0
18	Frontal-Parietal	1	0	1	1
19	Interfrontal	1	0	0	1
20	Parietal-Squamosal	0	0	0	0
21	Supraoccipital-Parietal	2	2	2	2
22	Basisphenoid-Basioccipital	0	0	1	0
23	Exoccipital-Basioccipital	2	2	2	0
24	Exoccipital-Supraoccipital	0	0	1	0
25	Orbitosphenoid-Frontal	0	0	0	0
26	Alisphenoid-Squamosal	0	0	0	0
[redacted]	Suture Closure Score	0.23	0.15	0.27	0.15
[redacted]	Breed	Zebu	Zebu	Polled	x
[redacted]	Inventory Number	IfH 1131	IfH 1130	SNM-KU No. Nmb.	SNM-KU MK 174
[redacted]	Age in months	30	30	30	30
[redacted]	Sex	Female	Female	Male	Male
1	Intermaxillary	0	0	0	0
2	Internasal	0	0	0	0
3	Nasal-Maxillary	0	0	0	0
4	Premaxillary-Maxillary (f)	0	0	0	0
5	Premaxillary-Maxillary (v)	0	0	0	0

6	Interpalatine	0	0	0	0
7	Maxillary-Lacrimal (f)	0	0	0	0
8	Maxillary-Palatine (v)	0	0	0	0
9	Jugal-Frontal	0	0	0	0
10	Lacrimal-Frontal (f)	0	0	0	0
11	Lacrimal-Frontal (o)	0	0	0	0
12	Lacrimal-Jugal (f)	0	0	0	0
13	Lacrimal-Jugal (o)	0	0	0	0
14	Maxillary-Lacrimal (o)	0	0	0	0
15	Nasal-Frontal	0	0	0	0
16	Basisphenoid - Presphenoid	0	0	0	0
17	Jugal-Squamosal	0	0	0	0
18	Frontal-Parietal	1	1	0	1
19	Interfrontal	1	1	0	0
20	Parietal-Squamosal	0	0	0	0
21	Supraoccipital-Parietal	2	2	2	2
22	Basisphenoid-Basioccipital	1	1	0	1
23	Exoccipital-Basioccipital	2	2	2	2
24	Exoccipital-Supraoccipital	1	1	0	0
25	Orbitosphenoid-Frontal	0	0	0	0
26	Alisphenoid-Squamosal	0	0	0	0
	Suture Closure Score	0.31	0.31	0.15	0.23
	Breed	Danish Red	Danish Red	Hinterwälder	x
	Inventory Number	SNM-KU 10/4-1957	SNM-KU 7/3 1955	ZNS Haustierkunde B htw 1	IfH 11321
	Age in months	33	36	36	37
	Sex	Female	Female	Female	Female
1	Intermaxillary	0	0	0	0
2	Internasal	0	0	0	0
3	Nasal-Maxillary	0	0	0	0
4	Premaxillary-Maxillary (f)	0	0	0	0
5	Premaxillary-Maxillary (v)	0	0	0	0
6	Interpalatine	0	0	0	0

7	Maxillary-Lacrimal (f)	0	0	0	0
8	Maxillary-Palatine (v)	0	0	0	0
9	Jugal-Frontal	0	0	0	0
10	Lacrimal-Frontal (f)	0	0	0	0
11	Lacrimal-Frontal (o)	0	0	0	0
12	Lacrimal-Jugal (f)	0	0	0	0
13	Lacrimal-Jugal (o)	0	0	0	0
14	Maxillary-Lacrimal (o)	0	0	0	0
15	Nasal-Frontal	0	0	0	0
16	Basisphenoid - Presphenoid	0	0	1	0
17	Jugal-Squamosal	0	0	0	0
18	Frontal-Parietal	1	1	1	1
19	Interfrontal	1	1	1	0
20	Parietal-Squamosal	0	0	0	1
21	Supraoccipital-Parietal	2	2	2	2
22	Basisphenoid-Basioccipital	2	2	2	0
23	Exoccipital-Basioccipital	2	2	2	2
24	Exoccipital-Supraoccipital	2	1	2	1
25	Orbitosphenoid-Frontal	0	0	0	0
26	Alisphenoid-Squamosal	0	0	0	0
	Suture Closure Score	0.38	0.35	0.42	0.27
	Breed	Scottish Highland	Niata	Zebu	Spanish Fighting
	Inventory Number	SNM-KU MK 572	MLP 1556	SNM-KU CN 319	SNM-KU CN 2812
	Age in months	42	48	48	48
	Sex	Male	Female	Male	Male
1	Intermaxillary	0	0	0	0
2	Internasal	0	1	1	1
3	Nasal-Maxillary	0	0	0	0
4	Premaxillary-Maxillary (f)	0	0	0	0
5	Premaxillary-Maxillary (v)	0	0	0	0
6	Interpalatine	0	0	0	0
7	Maxillary-Lacrimal (f)	0	0	0	0
8	Maxillary-Palatine (v)	0	0	0	0

9	Jugal-Frontal	0	0	0	0
10	Lacrimal-Frontal (f)	0	0	0	0
11	Lacrimal-Frontal (o)	0	0	0	0
12	Lacrimal-Jugal (f)	0	0	0	0
13	Lacrimal-Jugal (o)	0	0	0	0
14	Maxillary-Lacrimal (o)	0	0	0	0
15	Nasal-Frontal	0	0	0	0
16	Basisphenoid - Presphenoid	0	0	0	0
17	Jugal-Squamosal	0	0	0	0
18	Frontal-Parietal	1	1	1	1
19	Interfrontal	1	1	1	1
20	Parietal-Squamosal	0	0	1	1
21	Supraoccipital-Parietal	2	2	2	2
22	Basisphenoid-Basioccipital	2	2	2	2
23	Exoccipital-Basioccipital	2	2	2	2
24	Exoccipital-Supraoccipital	2	1	2	2
25	Orbitosphenoid-Frontal	1	1	1	0
26	Alisphenoid-Squamosal	0	0	0	0
	Suture Closure Score	0.42	0.42	0.50	0.46
	Breed	Spanish Fighting	Svensk Fjeldrace	Schwyz (Swiss Brown)	Shorthorn
	Inventory Number	SNM-KU CN 2815	SNM-KU MK 137	ZNS Haustierkunde B swyz 5	ZNS Haustierkunde B shh 14
	Age in months	60	60	64	72
	Sex	Male	Male	Female	Male
1	Intermaxillary	0	0	0	0
2	Internasal	0	0	0	1
3	Nasal-Maxillary	0	0	0	0
4	Premaxillary-Maxillary (f)	0	0	0	0
5	Premaxillary-Maxillary (v)	0	0	0	0
6	Interpalatine	0	0	0	0
7	Maxillary-Lacrimal (f)	0	0	0	0
8	Maxillary-Palatine (v)	0	0	0	0

9	Jugal-Frontal	1	0	1	1
10	Lacrimal-Frontal (f)	1	0	1	1
11	Lacrimal-Frontal (o)	0	1	1	1
12	Lacrimal-Jugal (f)	1	0	0	2
13	Lacrimal-Jugal (o)	1	1	0	1
14	Maxillary-Lacrimal (o)	0	0	0	0
15	Nasal-Frontal	0	1	0	0
16	Basisphenoid - Presphenoid	0	2	2	1
17	Jugal-Squamosal	0	0	0	0
18	Frontal-Parietal	1	1	1	1
19	Interfrontal	1	1	1	1
20	Parietal-Squamosal	1	1	2	1
21	Supraoccipital-Parietal	2	2	2	2
22	Basisphenoid-Basioccipital	1	2	2	2
23	Exoccipital-Basioccipital	2	2	2	2
24	Exoccipital-Supraoccipital	2	2	2	2
25	Orbitosphenoid-Frontal	1	0	1	1
26	Alisphenoid-Squamosal	1	0	2	1
	Suture Closure Score	0.62	0.62	0.77	0.81
	Breed	Jersey	Niata	Dexter	Red Danish
	Inventory Number	SNM-KU MK 341	MLP 1465	IfH 33421	RDM 14/11-1959
	Age in months	84	72	96	99
	Sex	Male	Female ?	Female	Female
1	Intermaxillary	0	0	0	0
2	Internasal	0	1	0	1
3	Nasal-Maxillary	0	0	0	0
4	Premaxillary-Maxillary (f)	0	0	0	0
5	Premaxillary-Maxillary (v)	0	0	0	0
6	Interpalatine	0	0	0	0
7	Maxillary-Lacrimal (f)	0	0	0	0
8	Maxillary-Palatine (v)	0	0	0	0
9	Jugal-Frontal	0	0	0	1
10	Lacrimal-Frontal (f)	1	0	1	1

11	Lacrimal-Frontal (o)	1	0	0	0
12	Lacrimal-Jugal (f)	0	0	0	0
13	Lacrimal-Jugal (o)	0	0	1	0
14	Maxillary-Lacrimal (o)	0	0	0	0
15	Nasal-Frontal	0	0	0	0
16	Basisphenoid - Presphenoid	2	2	2	2
17	Jugal-Squamosal	0	0	0	0
18	Frontal-Parietal	1	1	1	1
19	Interfrontal	1	1	1	1
20	Parietal-Squamosal	1	1	1	1
21	Supraoccipital-Parietal	2	2	2	2
22	Basisphenoid-Basioccipital	2	2	2	2
23	Exoccipital-Basioccipital	2	2	2	2
24	Exoccipital-Supraoccipital	2	2	2	2
25	Orbitosphenoid-Frontal	1	2	2	1
26	Alisphenoid-Squamosal	1	2	1	2
	Suture Closure Score	0.65	0.69	0.69	0.73
	Breed	Niata	x	x	Angeln
	Inventory Number	MLP 1126	SNM-KU MK 177	SNM-KU MK 176	ZNS Haustierkunde B agl 7
	Age in months	144	120	124	126
	Sex	Male	Male	Female	Female
1	Intermaxillary	0	1	1	0
2	Internasal	2	2	0	0
3	Nasal-Maxillary	0	0	0	0
4	Premaxillary-Maxillary (f)	1	0	0	0
5	Premaxillary-Maxillary (v)	0	1	0	0
6	Interpalatine	1	0	0	0
7	Maxillary-Lacrimal (f)	2	1	0	0
8	Maxillary-Palatine (v)	1	0	0	0
9	Jugal-Frontal	2	2	1	0
10	Lacrimal-Frontal (f)	1	1	1	0
11	Lacrimal-Frontal (o)	2	1	1	0

12	Lacrimal-Jugal (f)	2	2	0	0
13	Lacrimal-Jugal (o)	2	2	0	0
14	Maxillary-Lacrimal (o)	0	0	0	0
15	Nasal-Frontal	2	1	0	0
16	Basisphenoid - Presphenoid	2	2	2	2
17	Jugal-Squamosal	1	1	0	0
18	Frontal-Parietal	2	1	1	1
19	Interfrontal	1	1	1	1
20	Parietal-Squamosal	2	1	2	1
21	Supraoccipital-Parietal	2	2	2	2
22	Basisphenoid-Basioccipital	2	2	2	2
23	Exoccipital-Basioccipital	2	2	2	2
24	Exoccipital-Supraoccipital	2	2	2	2
25	Orbitosphenoid-Frontal	2	2	1	1
26	Alisphenoid-Squamosal	2	1	1	1
	Suture Closure Score	1.46	1.19	0.77	0.58
	Breed	x	Galloway	x	German Black Pied
	Inventory Number	SNM-KU MK 166	ZNS Haustierkunde B glw 3	SNM-KU MK 179	IfH 24769
	Age in months	132	159	174	252
	Sex	Female	Female	Female	Female
1	Intermaxillary	0	0	0	0
2	Internasal	0	0	1	1
3	Nasal-Maxillary	0	0	0	0
4	Premaxillary-Maxillary (f)	0	0	0	0
5	Premaxillary-Maxillary (v)	0	0	0	0
6	Interpalatine	0	0	0	1
7	Maxillary-Lacrimal (f)	0	0	0	0
8	Maxillary-Palatine (v)	0	0	1	1
9	Jugal-Frontal	1	1	1	2
10	Lacrimal-Frontal (f)	1	1	1	1
11	Lacrimal-Frontal (o)	1	1	1	2

12	Lacrimal-Jugal (f)	0	2	1	2
13	Lacrimal-Jugal (o)	1	1	1	1
14	Maxillary-Lacrimal (o)	0	0	0	0
15	Nasal-Frontal	0	0	0	0
16	Basisphenoid - Presphenoid	2	2	2	2
17	Jugal-Squamosal	0	1	1	1
18	Frontal-Parietal	1	1	1	2
19	Interfrontal	1	1	1	1
20	Parietal-Squamosal	1	2	2	2
21	Supraoccipital-Parietal	2	2	2	2
22	Basisphenoid-Basioccipital	2	2	2	2
23	Exoccipital-Basioccipital	2	2	2	2
24	Exoccipital-Supraoccipital	2	2	2	2
25	Orbitosphenoid-Frontal	2	2	2	2
26	Alisphenoid-Squamosal	2	2	2	2
	Suture Closure Score	0.81	0.96	1.00	1.19
	Breed	Jersey	x	x	
	Inventory Number	IfH 142	SNM-KU MK 161	SNM-KU MK 169	
	Age in months	288	318	348	
	Sex	Female	Female	Female	
1	Intermaxillary	0	0	0	
2	Internasal	0	0	1	
3	Nasal-Maxillary	0	0	0	
4	Premaxillary-Maxillary (f)	0	0	0	
5	Premaxillary-Maxillary (v)	0	1	1	
6	Interpalatine	0	1	0	
7	Maxillary-Lacrimal (f)	0	0	0	
8	Maxillary-Palatine (v)	0	1	0	
9	Jugal-Frontal	0	1	1	
10	Lacrimal-Frontal (f)	1	1	1	
11	Lacrimal-Frontal (o)	0	0	0	
12	Lacrimal-Jugal (f)	0	1	0	
13	Lacrimal-Jugal (o)	0	0	1	

14	Maxillary-Lacrimal (o)	0	0	0
15	Nasal-Frontal	0	0	0
16	Basisphenoid - Presphenoid	2	2	2
17	Jugal-Squamosal	1	1	1
18	Frontal-Parietal	1	1	1
19	Interfrontal	1	1	1
20	Parietal-Squamosal	2	1	1
21	Supraoccipital-Parietal	2	2	2
22	Basisphenoid-Basioccipital	2	2	2
23	Exoccipital-Basioccipital	2	2	2
24	Exoccipital-Supraoccipital	2	2	2
25	Orbitosphenoid-Frontal	1	1	1
26	Alisphenoid-Squamosal	2	2	1
	Suture Closure Score	0.73	0.88	0.81

Supplementary Table 2: Basicranial angle of examined cattle breeds.

Breed (n: 36)	Average Angle	StDev	n	Dorso-Basal Curvature
Abessinian	173	-	1	klinorhynchy
Andalusian	165	-	1	klinorhynchy
Angeln	169	0	2	klinorhynchy
Bos primigenius (Aurochs)	158	1.71	4	klinorhynchy
Brazilian	171	6.24	3	klinorhynchy
Cretan	166	2.83	2	klinorhynchy
Danish Red	174	-	1	klinorhynchy
Dexter	179	-	1	klinorhynchy
Galloway	172	-	1	klinorhynchy
German Black Pied	169	-	1	klinorhynchy
Graubündner (Swiss Brown)	171	-	1	klinorhynchy
Guernsey	168	-	1	klinorhynchy
Hinterwälder	169	-	1	klinorhynchy
Iceland	170	0	2	klinorhynchy
Jamtland	169	3.21	5	klinorhynchy
Japanese	167	-	1	klinorhynchy
Jersey	178	3.64	8	klinorhynchy
Jutland	165	2.12	2	klinorhynchy
Kerry	172	-	1	klinorhynchy
Limousin	167	-	1	klinorhynchy
Longhorn (British)	172	-	1	klinorhynchy
Niata	193	10.61	2	airorhynchy
Red Poll	170	-	1	klinorhynchy
Sanga	160	-	1	klinorhynchy
Sardinian	165	4.24	2	klinorhynchy
Schwyz (Swiss Brown)	170	3.54	2	klinorhynchy
Scottish Highland	168	-	1	klinorhynchy
Shorthorn (British)	169	2.08	3	klinorhynchy
Sicilian	179	-	1	klinorhynchy
Småland	171	7.78	2	klinorhynchy
Spanish Fighting	166	1.99	7	klinorhynchy
Swedish Mountain	170	-	1	klinorhynchy
Watussi	169	2.83	2	klinorhynchy
White Park	169	1.41	2	klinorhynchy
Wilstermarsch (German Red Pied)	168	-	1	klinorhynchy
Zebu	164	6.32	9	klinorhynchy

Supplementary Table 3: Body size estimates for the Niata specimen MLP 1126.

source	skeletal part	measurement (cm)	estimated bodyweight (kg)
Scott 1983	humerus length (H2)	32.3	411.63
Anderson et al. 1985	humerus circumference	16.7	
	femur circumference	15.5	545.06
Janis 1990	m1 length	2.2	
	m2 length	2.6	335.48
		**Average**	430.73
		**SD**	106.09

Supplementary Table 4: Body sizes of taurine cattle breeds as published by Felius 1995 and Niata.

Breed	Medium Steer weight (kg)	Medium Steer height (cm)	Medium Cow weight (kg)	Medium Cow height (cm)
Aberdeen-Angus	1000	135	650	125
Abondance	1000	147	600	133
Agerolese	650	135	450	125
Akshi	-	135	200	105
Ala Tau	900	-	485	130
Alberes	362.5	-	285	125
Algerian Guelma	400	125	250	115
Alistana-Sanabresa	800	150.5	575	142
Anatolian Black	-	-	225	107.5
Andalusian Black	812.5	140	550	135
Angeln	975	145.5	560	131
Aosta Black Pied	600	130	500	125
Aosta Red Pied	650	132.5	500	130
Apulian Podolian	700	152.5	405	127.5
Argentine Criollo	700	160	450	135
Argentine Criollo (Fronterizo)	-	-	325	135
Arouquesa	660	134	405	123
Asturian Mountain	675	132.5	450	122.5
Asturian Valley	1000	147.5	700	135
Aubrac	825	140	580	130
Aulie Ata	885	140	495	130
Aure et St Girons	-	-	600	135
Aurochs (*Bos primigenius*)	800	180	600	160
Austrian Pinzgauer	1000	143.5	650	134
Avilena-Black Iberian	825	145	550	138
Ayrshire	700	140	550	131
Baoule	265	105	175	95
Barrosa	625	127.5	367.5	118.5
Barroso	812.5	150	450	135
Bazadais	1050	145	700	135
Beef Shorthorn	1025	145	650	135
Belgian Red	1200	153	687.5	138
Belgian White and Red	1350	155	750	138
Belgian White Blue	1275	151.5	750	132.5

Bérnais	-	130	550	125
Berrenda Black Pied	900	143	575	138
Berrenda Red Pied	900	143	575	138
Bestuzhev	870	140	520	130
Black Pied Dairy Cattle	-	-	600	135
Blacksided Trondheim and Nordland	850	-	445	117
Blonde d'Aquitaine	1400	160	950	150
Bohemian Red	-	-	500	125
Brazilian Polled	750	132	420	130
Breton Black Pied	650	123	450	117
British White	850	120	575	110
Burlina	625	130	525	120
Byelorus Red	750	135	460	130
Cabannina	550	125	375	120
Cachena	412.5	122	265	100
Caldelana	675	132	450	128
Calvana	1025	157.5	700	147.5
Camargue	437.5	130	304.5	120
Canary Island	900	152	575	148
Carinthian Blond	825	140	550	132.5
Caucasian Brown	780	-	485	126.5
Cauyen Red	855	138	450	125
Chaco Criollo	-	-	500	155
Charolais	1425	145	975	135
Chianina	1215	167.5	900	155
Chillingham	300	-	280	110
Chinese Black and White	1020	150	562.5	135
Cinesara	650	145	475	135
Corsican	450	130	340	115
Croatian Busa	-	-	215	111
Czech Pied	1300	150	700	140
Dairy Shorthorn	1200	150	675	140
Dalmatian Grey	-	-	275	122
Danish Jersey	-	-	450	120
Danish Red	925	154	637.5	137
Devon	1000	-	500	130

Dexter	450	95	300	95
Donnersberg Red	1106	150	625	138
Doran	500	-	350	120
Dutch Black Pied (Holstein-Friesian)	1100	165	750	150
East Finncattle	600	135	440	118
East German Black Pied Cattle	1000	140	650	130
Estonian Black Pied	900	138	500	128
Estonian Native	800	134	520	125
Estonian Red	850	135	500	128
Ferrandais	-	-	650	138
Fighting Cattle	650	127.5	350	115
Forest Muturu	-	-	200	89
Froment du Léon	-	-	550	135
Fuzhou	765	138	415	130
Gacko	-	-	300	117
Galician Blond	1000	145	650	135
Galloway	750	135	520	120
Garfagnina	600	140	425	125
Gascon	850	145	550	135
Gelbvieh	1200	152	750	140
German Original Black Pied	900	155	600	136
German Pinzgauer	900	137	700	135
German Red Pied	1300	150	700	137
German-Angus	1100	142.5	625	132.5
Ghana Shorthorn	-	-	185	88
Glan cattle	1000	148	650	140
Gloucester	750	130	562.5	125
Golpayegani	-	-	230	110
Gorbatov Red	830	133	480	122
Greater Caucasus	-	-	250	104
Greek Shorthorn	-	-	275	107
Greek Steppe	400	123	265	112.5
Grey Alpine	950	133	575	126
Groningen Whiteheaded	-	-	600	137.5
Guernsey	800	152	500	137
Guinean N'Dama	305	118	255	112

Harz Red	1100	150	600	135
Hazake	435	120	300	110
Heckrind	600	135	-	125
Hereford	1100	152	700	140
Hérens	775	128	610	123
Highland	700	128	470	115
Hinterwald	775	132.5	415	119
Holstein	1125	175	850	155
Hungarian Grey Steppe	750	150	535	138
Icelandic Dairy Cattle	-	-	425	130
Iskar	750	140	400	118
Istoben	755	-	440	127.5
Istrian	825	148	585	137.5
Japanese Black	825	135	517.5	125
Japanese Brown	950	143	600	130
Japanese Shorthorn	950	142	660	128
Jaulan	-	-	350	114
Jersey	550	127	387.5	117.5
Kalmyk	750	137	537.5	127.5
Kapsiki	380	115	230	110
Kazakh	500	130	272.5	115
Kazakh Whiteheaded	825	-	530	134
Kerry	590	140	375	122
Kholmogory	885	145	580	132.5
Kolubara	550	-	380	124
Korean Native	460	135	370	125
Kostroma	850	140	520	132
Kurgan	850	140	535	130
Lages Criollo	-	140	475	127
Lagune	-	95	155	85
Lakenvelder	700	137	550	132
Latvian Brown	800	140	455	130
Lebedin	900	142	575	133
Leonese	650	136	500	130
Liberian Dwarf	-	-	120	90
Libyan Shorthorn	380	120	290	110

Limousin	1200	145	700	137
Limpurger	1000	145	625	135
Lithuanian Black Pied	950	140	550	129
Lithuanian Red	750	135	490	126
Longhorn	1000	150	850	135
Lourdais	-	-	525	135
Luing	900	138.5	-	-
Maine-Anjou	1259	152	850	142
Marchigiana	1300	157.5	800	145
Maremmana	900	155	600	145
Marinhoa	875	150	585	140
Maronesa	600	140	375	130
Menorcan	850	144	612.5	138
Meuse-Rhine-Yssel	1100	150	600	135
Middle German Red	-	-	625	130
Mingrelian Red	465	-	300	112.5
Mirandais	750	130	450	120
Mirandesa Beiroa	950	147.5	575	137.5
Mishima	450	123	280	112.5
Modenese	1050	155	650	145
Modicana	925	152.5	505	137.5
Mongolian Gobi	-	-	270	107
Mongolian Halhin Gol	117.5	377.5	360	115
Mongolian Intermediate	-	-	350	110
Montana	700	140	500	130
Montbéliard	1150	148	685	137.5
Moroccon Brown Atlas	375	135	300	115
Morucha	750	145	475	140
Mostrenca	900	145	550	135
Murboden	900	145	600	135
Murcian	850	140	575	132.5
Murnau-Werdenfels	925	138	550	128
Namchi	200	103	186	99
Niata	430	130	-	-
Normande	1100	152	750	140
North Finncattle	550	128	350	112.5

Northern Blue	1050	148	725	135
Norwegian Red	1000	142	575	130
Original Allgäu	900	145	550	135
Oropa	850	135	625	125
Oulmes-zaer	450	135	325	120
Palmera	850	138	487.5	133
Parthenais	1075	142.5	725	135
Piedmont	900	150	600	145
Pirenaica	950	140	575	132.5
Pisana	815	160	525	147.5
Polish Black-and-White Lowland	650	132	550	128
Polish Red Highland	775	135	465	122.5
Pontremolese	750	145	475	128
Posavina	600	-	300	120
Pustertal	800	138.5	500	129
Red Flemish	1100	148	675	140
Red Poll	750	145	525	129.5
Red Polled Eastland	775	140	475	123
Red Steppe	900	139	500	128.5
Reggiana	650	150	450	140
Rendena	600	130	475	125
Retinta	825	146	540	137.5
Rhaetian Grey Cattle	750	130	450	117.5
Rodopi	350	115	240	95
Romagnola	1150	145	800	135
Romanian Brown	1050	142	550	128
Romanian Mountain	-	-	215	104
Romanian Simmental	1040	148	600	135
Romanian Steppe	600	130	300	118
Romosinuano	650	135	550	130
Russian Swiss	850	139	500	131
Salers	975	150	700	140
San Martinero	800	135	475	130
Sanhe	1050	157.5	697.5	132.5
Sardinian	290	105	250	102.5
Sardo-Modicana	600	145	525	135

Sayaguesa	1050	160	650	145
Serrano	-	-	500	140
Shetland	-	-	290	105
Slovakian Pied	1050	145	650	135
Slovakian-Carpathian Brown	800	-	450	125
Slovanian Steppe	-	-	465	127
Slovenian and Croatian Simmental	1150	152	575	137
Slowakian Pinzgauer	850	136	550	128
Slowenian/Yugoslavian Brown	1100	152	600	134
Somba	205	-	160	94.5
South and West Norwegian	812.5	136	475	117
South Devon	1250	155	675	138
Suksun	920	-	390	130
Sussex	950	150	600	140
Swedish Mountain	590	130	395	120
Swedish Red -and-White	975	135	600	130
Swedish Red Polled	650	130	400	120
Swiss Brown	1087.5	150	675	137.5
Swiss Simmental	1300	154	800	141
Sychevka	1050	146	630	133
Tagil	887.5	-	480	130
Tambov Red	700	136	460	127
Tarentaise	800	140	575	127
Telemark	825	-	400	120
Texas Longhorn	670	130	400	120
Tibetan	215	105	200	100
Transylvanian Pinzgauer	900	134	450	127
Tudanca	540	135	370	130
Turkish Brown	-	-	600	140
Turkish Grey Steppe	400	-	340	120
Tux-Zillertal	-	-	550	125
Tyrol Grey	950	137.5	570	127.5
Ukrainian Polish Red	750	135	465	125
Ukrainian Steppe	800	137	525	129
Ukrainian Whiteheaded	750	136	425	128
Urkainian-Carpathian Brown	910	137	490	128

Vianesa	850	138.5	525	135
Villard-de-Lans	-	-	600	140
Vogelsberg Cattle	600	138	500	128
Vorderwald	975	148	625	132.5
Vosges	700	142.5	500	130
Waldviertler Blond	900	140	575	132
Welsh Black	1000	145	675	130
West Finncattle	737.5	140	470	123
Westphalian Red	1100	150	650	135
White park	955	-	542.5	130
Xinjiang Brown	760	137	432.5	121.5
Yakut	525	122	375	111
Yanbien	467.5	130	365	120
Yaroslavl	822	-	480	128
Yugoslavian Busa	405	117.5	200	105
Yurino	775	132	490	122.5

Supplementary Table 5: Definition of the landmarks used on the skull in this study.

LM	Definition
1	Medialmost point of the alveolar process (premaxilla) sin
2	Medialmost point of the alveolar process (premaxilla) dext
3	Lateralmost point of the anterior part of the premaxilla sin
4	Lateralmost point of the anterior part of the premaxilla dext
5	Inferior-lateral sutureboarder between premaxilla and maxilla sin
6	Inferior-lateral sutureboarder between premaxilla and maxilla dxt
7	Superior-posteriormost point of the premaxillary bone (nasal process) sin
8	Superior-posteriormost point of the premaxillary bone (nasal process) dxt
9	Anteriormost point of the medial processus of the nasal bone sin
10	Anteriormost point of the medial processus of the nasal bone dxt
11	Medial boarder between nasal bone and frontal bone
12	Boarder between lacrimal, maxillar and zygomatic bone sin
13	Boarder between lacrimal, maxillar and zygomatic bone dxt
14	Inferior suture between lacrimal bone and orbit sin
15	Inferior suture between lacrimal bone and orbit dxt
16	Superior suture between lacrimal bone and orbit sin
17	Superior suture between lacrimal bone and orbit dxt
18	Superiormost point of the orbit sin
19	Superiormost point of the orbit dxt
20	Boarder between frontal and temporal bone in the orbit sin
21	Boarder between frontal and temporal bone in the orbit dxt
22	Inferiormost point of the orbit sin
23	Inferiormost point of the orbit dxt
24	Anteriormost point of the zygomatic bone behind the orbit sin
25	Anteriormost point of the zygomatic bone behind the orbit dxt
26	Boarder between frontal and temporal bone behind the orbit sin
27	Boarder between frontal and temporal bone behind the orbit dxt
28	Superiormost point of the zygomatic arch sin
29	Superiormost point of the zygomatic arch dxt
30	Posteriormost point of the frontal bone in the midline
31	Medial-posteriormost point of the fossa temporalis sin
32	Medial-posteriormost point of the fossa temporalis dxt
33	Superiormost point of the occipital articulation surface sin
34	Superiormost point of the occipital articulation surface dxt
35	Lateralmost point of the occipital articulation surface sin
36	Lateralmost point of the occipital articulation surface dxt
**Skull turned around**	
37	Anteriormost point of the palatine fissure sin
38	Anteriormost point of the palatine fissure dext
39	Inferior-medial sutureborder between premaxilla and maxilla sin
40	Inferior-medial sutureborder between premaxilla and maxilla dxt
41	Inferior-medial sutureborder between premaxilla (palatine process) and maxilla
42	Anterior boarder of premolar 2 sin
43	Anterior boarder of premolar 2 dext
44	Posterior boarder of molar 3 sin
45	Posterior boarder of molar 3 dext
46	Posteriormost point of the horizontal part of the palatal bone (in the midline)
47	Posteriormost suturepoint between zygomatic and temporal bone from ventral sin
48	Posteriormost suturepoint between zygomatic and temporal bone from ventral dxt
49	Inferior-Medial boarder of the foramen magnum

50 Medialmost point of the flexure between dorsal and ventral part of the occipital articulation surface sin
51 Medialmost point of the flexure between dorsal and ventral part of the occipital articulation surface dxt
52 Tip of the notch above the opening of the acoustic canal sin
53 Tip of the notch above the opening of the acoustic canal dxt

Supplementary Table 6: Details for the CT scans used for FEA analyses.

Skull speci-men ID	Slice thick-ness [mm]	Inter-slice dis-tance [mm]	Cur-rent [µA]	Volt-age [kV]	Expo-sure time [ms]	Voxel size [mm]	Nr. im-ages	Images exported as DI-COM stacks imple-mented in soft-ware	CT Scan-ner type	Scanner location
Niata MLP 1126	0.67	0.33	229	120	655	0.976 563	194 3	Version 2.6.2	Scan Philips Bril-liance 64	Centro de Imágenes Médicas (CIMED), La Plata, Argentina
Sim-mental-er ZMUZH 17765	1.00	0.20	132	120	1000	0.947 266	285 0	syngo CT VA50A	Sie-mens SOMA-MA-TOM Force	Universi-tätsspital Zürich, Zürich, Switzerland
Zebu ZMUZH 17767	1.00	0.20	132	120	1000	0.904 297	275 0	syngo CT VA50A	Sie-mens SOMA-MA-TOM Force	Universi-tätsspital Zürich, Zürich, Switzerland

Supplementary Table 7: Muscle forces used for each jaw muscle group in un-scaled intrinsic models. Muscle forces were calculated using muscle mass proportions following the ‘dry skull’ method, where maximum cross-sectional area (CSA) was calculated for each muscle group[15]. Muscle forces were scaled relative to body mass for each specimen following a 2/3 power relationship, whereby muscle force is proportional to cross-sectional area whereas body mass is proportional to volume[16]. MLP 1126 was used as the target (=reference) specimen for scaling.

**Jaw muscle group**	**Muscle force (Newtons) per jaw muscle group (one side)**		
	Niata (MLP 1126)	Simmentaler (ZMUZH 17765)	Zebu (ZMUZH 17767)
m. masseter	1434.59	2304.43	2178.16
m. temporalis	957.98	1538.82	1454.51
m. pterygoideus lateralis	309.32	496.87	469.64
m. pterygoideus medialis	889.87	1429.42	1351.11
TOTAL	3951.75	5769.54	5453.42
Scaled muscle force	1.00	1.46	1.38

Supplementary Table 8: Measurements of all examined Niata skulls.						
**Skull**						
**von den Driesch 1976 (sin/dex averaged)**		**MLP 1126**	**MLP 1556**	**MLP 1465**	**ZMB_Mam_105902**	**MNHN 1933-122**
1	total length	39.6	36.9	32.6	34.4	37
2	condylobasal length	38.6	37.5	33	34	36
3	basal length	34.3	34.8	30.7	31.7	33
4	small skull length	27.5	27.6	23.4	25.6	-
5	premolar - prosthion	11.7	10.6	9.7	9.4	-
6	neurocranial length	25	23.1	24	20.5	-
7	viscerocranial length	19.2	17.5	13.9	14.9	17.4
8	median frontal length	20	18.5	16.8	18.1	18.5
9	biggest frontal length	20.5	23	20.4	21.7	22.8
10	small upper skull length	28.8	27.2	24.9	26.5	27.3
11	akrokranion - Infraorbital	29.5	30	25.2	28.1	-
12	biggest nasal length	9.1	8.8	7.8	8.6	8.6
13	posterior margin of condylus occipitale - entorbitale	23	22.9	18.2	18.7	-
14	lateral face length	23.9	24.5	21	21.4	22.7
15	posterior margin of condylus occipitale - infraorbitale	28.5	28.8	23	25.6	-
16	infraorbitale - prosthion	12.5	17.3	10.3	10.5	-
17	prosthion - posterior margin of M3	23.3	23.4	20.7	17.6	21.3
18	prosthion - anterior margin of palate bone from ventral	16.8	15.2	14	14	14.9
19	premaxillary length	12.2	11	11.4	10.7	12.3
20	length tooth row	11.4	12.4	10.4	11.4	-
21	length molar row	7.5	8.1	7.1	6.8	8.7
22	length premolar row	5.3	5.3	4.6	6.4	-
23	length orbita	5.4	6.5	5.4	5.8	5.8
24	width orbita	6.4	6.7	5.6	6.9	6.6
25	mastoid width	25.5	23.5	17.2	20.7	24.3
26	width between occipital condyli	11.4	10.8	8.8	10.4	9.6
27	width between the bases of the processi jugulare	17.5	17.4	12.4	16	-
28	width foramen magnum	4.3	3.6	3.5	3.1	3.2
29	height foramen magnum	4.5	4	3.4	4.3	3.7
30	parietal width	18.5	16.8	11.1	13.5	14.8

31	width between bases of the horns	19.5	22.1	15.9	22.2	16.5
32	smallest frontal width	21.3	20.9	15.2	19	20
33	biggest frontal width between orbits	24	24	19.1	21	23.2
34	smallest frontal width between orbits	17.5	17	14.2	15	17.6
35	cheek width	19.5	18.5	16.3	15.3	-
36	nasal width	-	7	5.6	6.1	7.6
37	width of os incisivi	8.9	8.7	7.5	7	8.2
38	biggest width between lateral margins of alveola	15.2	14.2	13	12.2	-
39	height fossa temporalis	3.7	4	3.3	4.5	4.5
40	biggest height of occipital	17.5	18.5	13.9	15.3	16.4
41	smallest height of occipital	13.3	14.9	10.6	12.3	12.7
42	width between tips of the horns (without curvature)	60.5	69	54.2	59	79.2
	length P2	1.8	1.6	-	-	-
	width P2	1.6	1.3	-	-	-
	length P3	1.9	1.8	-	-	-
	width P3	1.6	1.5	-	-	-
	length P4	1.9	1.8	1.7	-	-
	width P4	1.9	1.7	1.9	-	-
	length M1	2	2.5	2.1	2.8	2.3
	width M1	2.1	2	2	1.9	1.9
	length M2	2.6	2.9	2.5	3	2.9
	width M2	2.2	1.9	2.3	1.7	1.9
	length M3	3	2.9	2.8	-	2.6
	width M3	2.3	1.6	1.9	-	1.7
	**Mandible**					
1	length mandible (gonion caudale - infradentale)	34.7	34.3	30	30	-
2	length mandible (posterior margin of proc. condyloideus-infradentale)	32.9	32.3	29	29.6	-
3	width ascending ramus	11.4	10.7	9	9.4	-
4	length body of mandible	23.3	23.2	21.1	22	-
5	gonion caudale - anterior alveola p2	24.7	25	21.5	22.9	-
6	gonion caudale - foramen mentale	28.4	28.4	25.7	26.6	-
7	length tooth row	13.7	14.4	12.8	13.7	-
8	length molar row	9.3	9.4	8.5	8.4	-

9	length premolar row	5.1	5.5	4.8	5.8	-
11	length diastema	8.6	8	7.5	7.5	-
12	gonion ventrale - tip processus condyloideus	17.7	16.4	15.6	14	-
13	gonion ventrale - ventralmost tip of incisura mandibulae	15.5	15	14	13.5	-
14	length ascending ramus	23	21.1	20.2	19.8	-
15a	height mandible posterior to m3	8.1	7.7	6.1	6.9	-
15b	height mandible between p4 and m1	5.3	5.4	4.3	4.9	-
15c	height mandible anterior to p2	4.1	3.8	3.3	3.6	-
	length p2	1	1.1	-	-	-
	width p2	0.9	0.9	-	-	-
	length p3	1.8	1.9	-	-	-
	width p3	1.1	1	-	-	-
	length p4	2.2	2.2	2.1	2.9	-
	width p4	1.2	1.2	1.2	1.2	-
	length m1	2.2	2.5	2.2	2.7	-
	width m1	1.4	1.2	1.2	1.3	-
	length m2	2.6	2.8	2.5	2.4	-
	width m2	1.5	1.2	1.3	1.2	-
10	length m3	4	3.5	3.7	-	-
10	width m3	1.6	1.3	1.3	-	-

Supplementary Table 9: Postcranial measurements to compare frontlimb and hindlimb length between breeds.

Breed	Inventory No.	Sex	Humerus length (cm)	Radius length (cm)	Metacarpal length (cm)	Femur length (cm)	Tibia length (cm)	Metatarsal length (cm)	Frontleg length (cm)	Hindleg length (cm)
Zebu (Indian)	ZNS Haustierkunde B i zb 18	Male	31.5	27	14	37	31.8	17.5	72.5	86.3
Angeln	ZNS Haustierkunde B agl 2	Female	30	28.3	20.5	39.7	32.5	23.5	78.8	95.7
Zebu (Indian)	ZNS Haustierkunde B i zb 24	Male	35.5	35.2	24	45.5	40	28	94.7	113.5
Angeln	ZNS Haustierkunde B agl 7	Female	30.2	29.5	20.3	40.2	33.8	23.2	80	97.2
Schwyz (Swiss Brown)	ZNS Haustierkunde B swyz 5	Female	30	27.5	19.8	40	31.3	22.8	77.3	94.1
Holland	ZNS Haustierkunde B hld 18	Female	31	28.8	20.1	41.5	32.5	23.5	79.9	97.5
Longhorn (British)	ZNS Haustierkunde B lgh 1	Female	30.8	28	19.1	40.9	33	22.7	77.9	96.6
Longhorn (British)	ZNS Haustierkunde B lgh 3	Female	31.1	28.2	19.5	40.6	32.5	22.5	78.8	95.6
Zebu (Indian)	ZNS Haustierkunde B i zb 19	Female	24	24.3	17	31.3	27.5	20.5	65.3	79.3
Shorthorn (British)	ZNS Haustierkunde B shh 3	Male	35.8	31.6	20.7	47.7	34.2	24.9	88.1	106.8
Shorthorn (British)	ZNS Haustierkunde B shh 121	Female	31.8	29.8	22.7	42	33	26	84.3	101
Jersey	ZNS Haustierkunde B jrs 12	Female	27	25.8	18.2	37.2	29.4	21	71	87.6
Jersey	ZNS Haustierkunde B jrs 14	Female	30.1	27	19.8	38.8	32.6	22.9	76.9	94.3
Jersey	ZNS Haustierkunde B jrs 13	Female	28.6	26.8	19	37.7	31.8	22	74.4	91.5
Hereford	ZNS Haustierkunde B hrfd 1	Female	32.4	30.4	22	43.1	34.5	24.9	84.8	102.5
Breitenburger	ZNS HaustierkundeB brtb 5	Female	31.9	29.5	21.7	42.7	32.5	24.5	83.1	99.7
Jersey	ZNS Haustierkunde B jrs 10	Male	33.8	29.2	19.9	43.3	34.6	23.3	82.9	101.2
Vogelsberger	ZNS Haustierkunde B vgb 3	Female	29.6	27.5	19.7	39.7	31.9	23	76.8	94.6
Vogtländer	ZNS Haustierkunde B vgtl 2	Female	31	29.7	21.5	41.1	34.2	25.2	82.2	100.5
Ansbach-Triesdorfer	ZNS Haustierkunde B ans-tr 1	Female	30.2	28.2	21	40.4	32.5	24	79.4	96.9
Simmental	ZNS Haustierkunde B sim 13	Female	31.5	29.4	21.7	42.3	33.7	25	82.6	101
Silesian Red	ZNS Haustierkunde B sl rtv 2	Female	32.4	29.6	21.5	42.6	33	24.5	83.5	100.1
Watussi	ZNS Haustierkunde B wa 2	Female	29.8	28.5	19.9	40.1	33.9	23.1	78.2	97.1
Hungarian Grey	ZNS Haustierkunde B ug stp 4	?	30.5	29.8	21.2	41.1	34.8	25.2	81.5	101.1
Devon	ZNS Haustierkunde B dv 2	Female	26.8	24.8	17.6	35.6	28.4	20	69.2	84
Podolian	ZNS Haustierkunde B pd stp 1	Female	32.2	30.4	20.9	43.3	33.5	24.3	83.5	101.1
Prätigauer	ZNS Haustierkunde B prtg 4	Female	33.8	31.2	22.1	45.8	34.6	24.8	87.1	105.2
Prätigauer	ZNS Haustierkunde B prtg 3	Female	31.3	29.5	20.8	42.3	32.3	24	81.6	98.6
Frisian Red	ZNS Haustierkunde B fr r 1	Female	30	28.6	20.9	40	31.3	23.8	79.5	95.1

Devon	ZNS Haustierkunde B dv 4	Female	30.4	26.4	18.8	39	31	22.1	75.6	92.1
Dwarf Zebu	IfH 20159	Female	23.7	22.4	14.7	29.3	24.8	17.5	60.8	71.6
Black Pied	IfH 16507	Female	31.7	29.2	20.6	41.6	31.4	23.5	81.5	96.5
Black Pied	IfH 24769	Female	30	27.9	19.9	40.3	30.5	22.8	77.8	93.6
Heck	IfH 32545	-	32.8	29.8	19.5	41.2	33.9	23.2	82.1	98.3
Watussi	IfH 8664	Female	30.8	31.3	21.3	40.2	34.4	25	83.4	99.6
Watussi	IfH 1574	Male	32.3	31.2	20.9	44.6	34.9	24.4	84.4	103.9
Niata	MLP 1126	Male	32.2	29.9	19.5	41.2	33	22.8	81.6	97

Supplementary Table 10: Postcranial measurements to compare axial and appendicular skeleton between breeds.

Breed	Ca. No.	Sex	Frontlimb (cm)	Hindlimb (cm)	Average Limb Length (cm)	Cervical-Sacrum Length (cm)
Kerry	ZNS Haustierkunde B kry 3	Female	89.5	97	93.25	145
Watussi	ZNS Haustierkunde B wa 1	Male	128.5	136.5	132.5	192
Zebu (Indian)	ZNS Haustierkunde No nmb	Female	89.7	93.5	91.6	133.5
Indian Dwarf Zebu	ZNS Haustierkunde B zw zb 1	Female	69.5	69	69.25	92
Holland	ZNS Haustierkunde No nmb	Female	126	136	131	201
Niata	MLP 1126	Male	124.5	127.5	126	197
Red Pied	IfH 7218	Male	124.8	126.6	125.7	212.7

Supplementary Table 11: Procrustes distances among skulls from cattle breeds represented in morphospace.

	Ayrshire	Blondvieh: Carinthian	Blondvieh: Lavanttaler	Blondvieh: Mariahofer	Blondvieh: Murbodner	Blondvieh: Waldviertler
Ayrshire		0.04145858	0.04568212	0.0481806	0.04192959	0.04112138
Blondvieh: Carinthian	0.04145858		0.02268568	0.04410361	0.02160092	0.02441471
Blondvieh: Lavanttaler	0.04568212	0.02268568		0.03985392	0.03020312	0.02880286
Blondvieh: Mariahofer	0.0481806	0.04410361	0.03985392		0.04496145	0.04680739
Blondvieh: Murbodner	0.04192959	0.02160092	0.03020312	0.04496145		0.03177846
Blondvieh: Waldviertler	0.04112138	0.02441471	0.02880286	0.04680739	0.03177846	
Bos primigenius (Aurochs)	0.07093008	0.0612221	0.05920285	0.0744248	0.06107915	0.05684014
Brazilian	0.05696098	0.04855212	0.0502479	0.04753273	0.04407159	0.05123755
Montafon	0.04630264	0.02043162	0.02507415	0.04394026	0.02410098	0.02425462
Buša	0.04560968	0.03916672	0.03737979	0.04972352	0.04169473	0.02764485
Chianina	0.05727331	0.04261312	0.04332959	0.06016584	0.04649006	0.03764011
Chillingham	0.06252938	0.06804382	0.06527605	0.06438198	0.07006097	0.06017193
Devon	0.03309541	0.03418129	0.03836734	0.03854228	0.03305862	0.03643554
Egerlaender	0.06076628	0.03786217	0.03620175	0.05600836	0.04091772	0.04440701
Hérens	0.04234304	0.05647958	0.05547971	0.04777126	0.05291751	0.05349782
Fjällko	0.03893607	0.02938978	0.03435946	0.05161792	0.03812976	0.02509881
Bern Red Pied	0.04450029	0.01894205	0.02430595	0.04380573	0.02398069	0.02771246
Freiburg Red Pied	0.05841863	0.04295133	0.04786474	0.0543934	0.0432899	0.04653733
Simmental	0.04203723	0.03956863	0.04274552	0.04005688	0.03774863	0.04249473
South Moravian Red Pied	0.04547014	0.026307	0.02916775	0.04161196	0.03034678	0.03382951
Rubia Gallega	0.07522174	0.05409574	0.06027351	0.07853502	0.05309046	0.05139284
Allgaeuer Grey	0.05545357	0.04439598	0.04626755	0.03978667	0.04617771	0.04647115
Krainer Grey	0.04611448	0.04395034	0.04715144	0.05303002	0.04035527	0.04109348
Oberinntaler Grey	0.032581	0.03012498	0.03180937	0.04118202	0.02900536	0.03180227
Haná-Berne	0.05479099	0.03136373	0.03645954	0.05629895	0.03378817	0.03140024
Iceland	0.03321779	0.04926187	0.05361721	0.05516607	0.04886281	0.04485787
Jersey	0.04928422	0.06706818	0.06685773	0.05146604	0.06482129	0.06654353
Jersey (polled)	0.046877	0.06347792	0.06498357	0.04821187	0.05943391	0.06361918
Jutland	0.05252131	0.03576369	0.03892032	0.06156031	0.04390795	0.03057168
Kampeten: Styrian Bergscheck	0.03452227	0.03052628	0.0285883	0.03480781	0.03219736	0.02919254

Kampeten: Welser Schecken	0.06231373	0.04616969	0.04741743	0.05933208	0.04848935	0.04409933
Kerry	0.03543689	0.0334083	0.03780142	0.05207182	0.03785828	0.02672226
Cretan	0.06436702	0.05907785	0.05543973	0.05651424	0.05990687	0.04920387
Kuhlaender	0.04140128	0.0196602	0.02642788	0.04186169	0.02130145	0.02152577
Landschlag von Warnsdorf	0.0528842	0.04731202	0.05184264	0.04941892	0.044865	0.05012264
Landschlag von Winkelsdorf	0.06853204	0.04062382	0.04369393	0.0661983	0.04857035	0.04155981
Landschlag X Simmental	0.05672837	0.03404019	0.03665635	0.05556613	0.03473488	0.03952114
Limousin	0.04035789	0.04044946	0.04491207	0.04004022	0.03542762	0.04769658
Moravian Landschlag	0.04323182	0.0359338	0.04337642	0.04178116	0.03433548	0.03750949
Normande	0.03961834	0.04561506	0.04440367	0.03845904	0.04632123	0.05309553
North Wales	0.04038236	0.02739349	0.03253143	0.04327838	0.02759978	0.02503625
White Park	0.05521112	0.05929033	0.05850102	0.05805194	0.06182317	0.04958168
Pinzgauer	0.03504277	0.02943521	0.03574653	0.03651396	0.02446642	0.03428901
Pinzgauer X Scottish Highland	0.05902845	0.0543728	0.05143479	0.05309988	0.04757542	0.06078693
Pinzgauer X Simmentaler	0.05074525	0.06152537	0.06271501	0.05327875	0.05349779	0.06148398
Pinzgauer: Moelltaler	0.05108962	0.02744421	0.03224799	0.04466143	0.0274293	0.0300203
Pinzgauer: Pustertaler	0.071816	0.07532121	0.07106856	0.05869017	0.07238881	0.07453269
Pirenaica	0.03331536	0.03506909	0.03885921	0.03620295	0.03839296	0.03573131
Red Poll	0.06336932	0.06967291	0.07033559	0.05256456	0.06905703	0.06988514
Maas-Rhein-Ijsselschlag	0.03308227	0.02704046	0.03088575	0.03717815	0.02980659	0.02650736
Danish Red	0.04911076	0.02799221	0.02965098	0.05110631	0.03774435	0.02978659
Angeln	0.04292993	0.04095237	0.04036793	0.04481188	0.03814	0.03777714
Bohemian Red	0.04091239	0.01878645	0.0242754	0.04297238	0.02254212	0.02046472
Polish Red	0.0534471	0.03277301	0.03553664	0.05468765	0.03889612	0.02685246
Sanga	0.08324567	0.06127921	0.05996404	0.08007684	0.06783411	0.05930493
Sardinian	0.05683412	0.04198042	0.04062088	0.05987378	0.04656537	0.04603027
Scheinfelder	0.0593029	0.04289409	0.04253323	0.05644176	0.04302931	0.04235654
Schoenhengster	0.04652963	0.02176712	0.02539212	0.04683583	0.0247152	0.02290094
Holland Black Pied	0.0461939	0.0431021	0.04490939	0.04122318	0.04537137	0.04267554
Andalusian Black	0.05273037	0.03506691	0.03398604	0.05342524	0.03879405	0.03318252
Sweden (Jaemtland or Vatterbotten)	0.0580801	0.05929315	0.06003434	0.07492729	0.06463642	0.05953403
Scottish Highland	0.05001926	0.05727377	0.05982746	0.0500434	0.05034591	0.05621169
Sicilian	0.06159061	0.05756516	0.05803314	0.06168711	0.05196366	0.06399953

Småland	0.05316119	0.04405478	0.04771227	0.06632894	0.05031339	0.03641398
South Devon	0.0462412	0.02563752	0.03507044	0.05195239	0.03135909	0.02856157
Spanish Fighting Cattle	0.05389274	0.05919467	0.05673718	0.05078145	0.05803057	0.0604356
Buchara Grey	0.12661497	0.10285956	0.10395813	0.12211763	0.10763348	0.09775977
Hungarian Grey	0.04384131	0.03594663	0.04191359	0.04222561	0.03147786	0.04307875
Hungarian Grey X Bern Red Pied	0.06685376	0.06635114	0.07190107	0.06552472	0.06270357	0.07006501
Sudeten	0.04378285	0.03059601	0.03677814	0.04720786	0.03649404	0.03076469
Sudeten X Simmental	0.04213798	0.04925052	0.04711952	0.04638151	0.05188329	0.05195697
Sudeten X Tesstal?	0.05073924	0.03991095	0.04602137	0.04812805	0.04041977	0.04656831
Tarentaise	0.04310943	0.0471061	0.04114471	0.04139913	0.04805287	0.04438519
Telemark	0.03585532	0.03358829	0.03727013	0.04960964	0.03501106	0.02407105
Tudanca	0.05448215	0.03661116	0.03985649	0.05182328	0.04034021	0.0339334
Tuxer	0.08074834	0.08976983	0.08790813	0.06704336	0.0853241	0.09023329
Heck	0.03680974	0.03970676	0.04370497	0.04848608	0.03777958	0.03440533
Niata	0.2001066	0.21785852	0.21672426	0.1917154	0.2101028	0.2214515
Veredelter Landschlag	0.05387092	0.04180249	0.04548011	0.05881627	0.04117698	0.04350537
Vogtlaender	0.03652396	0.04223469	0.04475204	0.04034813	0.03791814	0.04750086
Waldviertler X Scheinfelder	0.04424611	0.03628689	0.03634589	0.03856881	0.04135505	0.03887055
Watussi	0.05480974	0.03945152	0.04357396	0.06131459	0.04096565	0.04534438
Zebu	0.06806406	0.05104088	0.05145921	0.07225489	0.05785764	0.04493392
Zebu (Africa)	0.06710952	0.05984224	0.06155172	0.07812052	0.05570806	0.0574628
Zebu (Asian)	0.05310591	0.05480562	0.0563118	0.05812017	0.05199334	0.05517691
Zebu (Indian)	0.0702599	0.0495255	0.04967786	0.06659112	0.05470104	0.04628484
Zillertaler	0.05075463	0.05929228	0.05790197	0.04280034	0.05450565	0.05831793

	Bos primigenius (Aurochs)	Brazilian	Montafon	Buša	Chianina	Chillingham
Ayrshire	0.07093008	0.05696098	0.04630264	0.04560968	0.05727331	0.06252938
Blondvieh: Carinthian	0.0612221	0.04855212	0.02043162	0.03916672	0.04261312	0.06804382
Blondvieh: Lavanttaler	0.05920285	0.0502479	0.02507415	0.03737979	0.04332959	0.06527605
Blondvieh: Mariahofer	0.0744248	0.04753273	0.04394026	0.04972352	0.06016584	0.06438198
Blondvieh: Murbodner	0.06107915	0.04407159	0.02410098	0.04169473	0.04649006	0.07006097
Blondvieh: Waldviertler	0.05684014	0.05123755	0.02425462	0.02764485	0.03764011	0.06017193
Bos primigenius (Aurochs)		0.05795143	0.05485477	0.06291999	0.05669677	0.07885418

Brazilian	0.05795143		0.04334788	0.05602378	0.05394826	0.07041845
Montafon	0.05485477	0.04334788		0.03397181	0.03872687	0.06375185
Buša	0.06291999	0.05602378	0.03397181		0.04735867	0.05563863
Chianina	0.05669677	0.05394826	0.03872687	0.04735867		0.07510348
Chillingham	0.07885418	0.07041845	0.06375185	0.05563863	0.07510348	
Devon	0.07156995	0.04462448	0.03552495	0.04001785	0.04755113	0.06419388
Egerlaender	0.06987937	0.0611182	0.03901831	0.05268385	0.05494246	0.08615137
Hérens	0.07989819	0.05792069	0.05503425	0.05034229	0.072591	0.0540848
Fjällko	0.06540084	0.06025736	0.03385324	0.02879805	0.04706215	0.06536066
Bern Red Pied	0.05888646	0.04610689	0.02009628	0.03942585	0.04313193	0.0690759
Freiburg Red Pied	0.06496897	0.04975063	0.0432722	0.06275237	0.04890527	0.08109641
Simmental	0.06555693	0.03607501	0.03820072	0.04943872	0.05383947	0.06340088
South Moravian Red Pied	0.05697777	0.04157918	0.02836344	0.04581382	0.04625114	0.0673283
Rubia Gallega	0.06420744	0.06298685	0.04819643	0.05643811	0.05136276	0.08639266
Allgaeuer Grey	0.06423542	0.04553115	0.04427546	0.05467211	0.06258833	0.06490901
Krainer Grey	0.06468882	0.04976058	0.04420041	0.05253288	0.04758729	0.07296681
Oberinntaler Grey	0.06007611	0.04886537	0.02996574	0.03951443	0.05147103	0.05869594
Haná-Berne	0.05110096	0.05225589	0.03109418	0.04678487	0.04436539	0.07626295
Iceland	0.07494792	0.06132485	0.05200643	0.04544503	0.06473214	0.05848915
Jersey	0.10029968	0.07162385	0.07120834	0.06593598	0.07980806	0.07718128
Jersey (polled)	0.09428409	0.06718752	0.06438353	0.06237141	0.07579964	0.07434527
Jutland	0.05645617	0.06114146	0.03566687	0.03745221	0.04235048	0.06733402
Kampeten: Styrian Bergscheck	0.05636559	0.0449613	0.02947221	0.03445063	0.05045315	0.05569062
Kampeten: Welser Schecken	0.04363695	0.05537262	0.04240493	0.05506862	0.04880736	0.07390826
Kerry	0.06052267	0.05348561	0.03175527	0.02623326	0.04591078	0.05522087
Cretan	0.07698265	0.05665569	0.05369294	0.04068807	0.04943993	0.06769895
Kuhlaender	0.05911853	0.04577396	0.02328545	0.03609398	0.04028807	0.06562845
Landschlag von Warnsdorf	0.08587268	0.05651841	0.05175198	0.05128729	0.06589155	0.07724012
Landschlag von Winkelsdorf	0.06293015	0.06416328	0.04324698	0.05188176	0.05040062	0.08337797
Landschlag X Simmental	0.06813722	0.0573605	0.03331943	0.04476709	0.05242066	0.07420916
Limousin	0.07934974	0.04536744	0.04425001	0.05352391	0.0593372	0.07514389
Moravian Landschlag	0.07032274	0.04863008	0.03860797	0.04522441	0.05531119	0.06655889
Normande	0.08369193	0.05720895	0.0499145	0.05962417	0.06135516	0.07664625

North Wales	0.06178933	0.04062687	0.02678434	0.03453965	0.0387315	0.05843507
White Park	0.08362061	0.07021076	0.05817668	0.04136405	0.06793738	0.06035397
Pinzgauer	0.06740743	0.04154334	0.03126671	0.0444649	0.04634518	0.06476089
Pinzgauer X Scottish Highland	0.07356953	0.06549124	0.05327952	0.06545919	0.07465066	0.07751943
Pinzgauer X Simmentaler	0.08858689	0.06125236	0.06332775	0.06741579	0.07290662	0.07522347
Pinzgauer: Moelltaler	0.05960256	0.03970387	0.0237834	0.0419312	0.03491606	0.06915359
Pinzgauer: Pustertaler	0.08438199	0.06807053	0.07111485	0.07848648	0.08225006	0.0775206
Pirenaica	0.07086095	0.04327476	0.03825494	0.04248067	0.04934593	0.06056365
Red Poll	0.08253071	0.0583106	0.06868407	0.06950149	0.08238254	0.06553
Maas-Rhein-Ijsselschlag	0.0657585	0.04772667	0.02944362	0.0313126	0.04346927	0.06273233
Danish Red	0.06828854	0.06150761	0.03297571	0.03865895	0.04865581	0.07166504
Angeln	0.05900014	0.04455889	0.03670145	0.03556148	0.05100791	0.05483268
Bohemian Red	0.05885495	0.0488027	0.02150598	0.03161205	0.04241488	0.06688423
Polish Red	0.06465915	0.05770961	0.03243361	0.02840205	0.04310193	0.06525063
Sanga	0.07393976	0.07849673	0.05793314	0.05785965	0.06206184	0.0859188
Sardinian	0.07018777	0.06070054	0.04273589	0.055145	0.05101828	0.08131681
Scheinfelder	0.06114722	0.0501272	0.04005134	0.04250847	0.0512264	0.07342043
Schoenhengster	0.0572913	0.04945387	0.01981632	0.03535129	0.04218385	0.06673126
Holland Black Pied	0.06149029	0.04423005	0.04262663	0.04932859	0.05683121	0.06056219
Andalusian Black	0.04381656	0.04367314	0.02764374	0.03700149	0.04412854	0.0643616
Sweden (Jaemtland or Vatterbotten)	0.08591642	0.09054907	0.06362874	0.05901932	0.07986387	0.08617472
Scottish Highland	0.07457872	0.0460348	0.05436627	0.05957775	0.06816892	0.06349475
Sicilian	0.06893892	0.05825798	0.05908531	0.07267269	0.07213992	0.09147061
Småland	0.06846919	0.0672605	0.04410009	0.03618026	0.05375634	0.06327861
South Devon	0.06533874	0.04965205	0.02834701	0.03818905	0.03601091	0.07111721
Spanish Fighting Cattle	0.06594447	0.0502273	0.05532777	0.05926604	0.07538017	0.05638618
Buchara Grey	0.10571126	0.11200608	0.09798845	0.09868669	0.09151505	0.11748491
Hungarian Grey	0.06721091	0.03326778	0.03707033	0.05135918	0.05068916	0.06914704
Hungarian Grey X Bern Red Pied	0.07993064	0.04775365	0.06740633	0.07731051	0.07273043	0.08878886
Sudeten	0.0645172	0.05223672	0.03702177	0.04356036	0.04754669	0.06904169
Sudeten X Simmental	0.08808579	0.06891977	0.05565977	0.05537594	0.07089079	0.06821072
Sudeten X Tesstal?	0.07119904	0.0480865	0.04358297	0.05461022	0.0554933	0.07778613
Tarentaise	0.067714	0.05632764	0.04614827	0.04476911	0.06331203	0.05391231

Telemark	0.05921042	0.05357093	0.03365515	0.03138406	0.03981271	0.05971192
Tudanca	0.05470843	0.04615491	0.03275885	0.04207617	0.03600972	0.06766769
Tuxer	0.10231258	0.07640454	0.08796387	0.09162124	0.09940741	0.08383216
Heck	0.06455687	0.04360212	0.03921452	0.03889971	0.04680111	0.05474822
Niata	0.23560062	0.19940255	0.21836387	0.22076865	0.22550462	0.20999175
Veredelter Landschlag	0.06960616	0.05904333	0.04448275	0.04896987	0.05919109	0.07194605
Vogtlaender	0.07853255	0.0507037	0.04739855	0.05623123	0.06386175	0.07317974
Waldviertler X Scheinfelder	0.07533774	0.05357903	0.04055802	0.04440324	0.06124246	0.06309944
Watussi	0.06657304	0.06224117	0.04204826	0.05310046	0.0594277	0.08259489
Zebu	0.07369871	0.07309735	0.04797925	0.04114813	0.05261153	0.0762221
Zebu (Africa)	0.07473395	0.06988286	0.05694083	0.05412012	0.0686945	0.08520247
Zebu (Asian)	0.07939341	0.05977788	0.05674525	0.05955969	0.0549719	0.08092564
Zebu (Indian)	0.07110945	0.06492214	0.04539981	0.05003073	0.04525843	0.08024363
Zillertaler	0.08006397	0.05277231	0.05753961	0.06356017	0.06797234	0.06990026

	Devon	Egerlaender	Hérens	Fjällko	Bern Red Pied	Freiburg Red Pied
Ayrshire	0.03309541	0.06076628	0.04234304	0.03893607	0.04450029	0.05841863
Blondvieh: Carinthian	0.03418129	0.03786217	0.05647958	0.02938978	0.01894205	0.04295133
Blondvieh: Lavanttaler	0.03836734	0.03620175	0.05547971	0.03435946	0.02430595	0.04786474
Blondvieh: Mariahofer	0.03854228	0.05600836	0.04777126	0.05161792	0.04380573	0.0543934
Blondvieh: Murbodner	0.03305862	0.04091772	0.05291751	0.03812976	0.02398069	0.0432899
Blondvieh: Waldviertler	0.03643554	0.04440701	0.05349782	0.02509881	0.02771246	0.04653733
Bos primigenius (Aurochs)	0.07156995	0.06987937	0.07989819	0.06540084	0.05888646	0.06496897
Brazilian	0.04462448	0.0611182	0.05792069	0.06025736	0.04610689	0.04975063
Montafon	0.03552495	0.03901831	0.05503425	0.03385324	0.02009628	0.0432722
Buša	0.04001785	0.05268385	0.05034229	0.02879805	0.03942585	0.06275237
Chianina	0.04755113	0.05494246	0.072591	0.04706215	0.04313193	0.04890527
Chillingham	0.06419388	0.08615137	0.0540848	0.06536066	0.0690759	0.08109641
Devon		0.05198388	0.04395428	0.03646949	0.03494223	0.04975387
Egerlaender	0.05198388		0.0706161	0.04766145	0.03467359	0.05296667
Hérens	0.04395428	0.0706161		0.05443071	0.0551326	0.06681226
Fjällko	0.03646949	0.04766145	0.05443071		0.03374956	0.05658273
Bern Red Pied	0.03494223	0.03467359	0.0551326	0.03374956		0.03987062

Freiburg Red Pied	0.04975387	0.05296667	0.06681226	0.05658273	0.03987062	
Simmental	0.03524452	0.05554776	0.04028269	0.05047723	0.03699327	0.04446405
South Moravian Red Pied	0.04090541	0.04566856	0.05165371	0.04248905	0.02873317	0.04473824
Rubia Gallega	0.06433497	0.06236963	0.08740918	0.05989717	0.05424755	0.06343278
Allgaeuer Grey	0.04935956	0.05740989	0.05424937	0.05488076	0.04480879	0.05035184
Krainer Grey	0.04620313	0.05869029	0.05825666	0.05135333	0.04661268	0.04616141
Oberinntaler Grey	0.035362	0.04827201	0.04128763	0.03610294	0.02855792	0.04551884
Haná-Berne	0.04853142	0.04165455	0.06518531	0.04026387	0.0320187	0.04405213
Iceland	0.04073393	0.07089616	0.04192558	0.04122555	0.05211784	0.0668104
Jersey	0.04939675	0.08016218	0.05021225	0.06330574	0.06979548	0.0756177
Jersey (polled)	0.04773388	0.07940183	0.05118105	0.06371847	0.06533333	0.0738197
Jutland	0.0512945	0.05365359	0.06789287	0.03139728	0.04022344	0.05836319
Kampeten: Styrian Bergscheck	0.03386158	0.04763202	0.04010875	0.03653201	0.02885536	0.04946639
Kampeten: Welser Schecken	0.06078245	0.05813884	0.07154436	0.05565752	0.04449527	0.05218958
Kerry	0.0343283	0.05538626	0.04805885	0.02423758	0.03594035	0.05621864
Cretan	0.04924526	0.06927349	0.06706324	0.05379833	0.05992822	0.06971282
Kuhlaender	0.03221987	0.04003985	0.05206984	0.03213261	0.0240652	0.04434161
Landschlag von Warnsdorf	0.03947961	0.05730359	0.05094791	0.047944	0.0495598	0.06028473
Landschlag von Winkelsdorf	0.06126889	0.04920365	0.08036907	0.04662542	0.04304889	0.06114716
Landschlag X Simmental	0.0474073	0.0404866	0.06693832	0.04446289	0.0340922	0.05948201
Limousin	0.02915565	0.05600788	0.0498028	0.04902054	0.04274113	0.05024509
Moravian Landschlag	0.03552862	0.05608825	0.04898589	0.04361315	0.04059085	0.05060705
Normande	0.03623937	0.05849572	0.05300083	0.05290015	0.04794351	0.058522
North Wales	0.0270173	0.04869071	0.04897482	0.03466578	0.03002464	0.04371414
White Park	0.04705943	0.07054329	0.05064562	0.04481568	0.06074582	0.07599673
Pinzgauer	0.02517862	0.05047027	0.04365916	0.0412989	0.03098035	0.03919278
Pinzgauer X Scottish Highland	0.06033746	0.0560804	0.06240813	0.06625988	0.05032733	0.0658261
Pinzgauer X Simmentaler	0.04708433	0.07421976	0.04511493	0.0675096	0.06092176	0.05902019
Pinzgauer: Moelltaler	0.0349088	0.04165657	0.05957862	0.04145451	0.02534533	0.03892514
Pinzgauer: Pustertaler	0.07254632	0.08304038	0.06583582	0.0843537	0.072208	0.0794709
Pirenaica	0.02971606	0.05681196	0.04515009	0.04157507	0.03951584	0.04966014
Red Poll	0.06157651	0.08591209	0.0546287	0.07486053	0.06927664	0.07701759
Maas-Rhein-Ijsselschlag	0.02375714	0.04659032	0.04571217	0.0264277	0.03145225	0.04886808

Danish Red	0.0427413	0.03904281	0.06522596	0.02853915	0.03187625	0.0560535
Angeln	0.03710902	0.05720841	0.03957151	0.0419151	0.03983993	0.05441776
Bohemian Red	0.03399686	0.03742785	0.05092996	0.02689155	0.02153411	0.04350122
Polish Red	0.04282682	0.04913054	0.06262523	0.03041254	0.03793114	0.06102627
Sanga	0.07197535	0.06186087	0.09340492	0.06070583	0.06242533	0.08091916
Sardinian	0.05061024	0.0489214	0.07637989	0.05067935	0.04387853	0.06165589
Scheinfelder	0.04570541	0.05069248	0.06395935	0.04706131	0.03971706	0.05568409
Schoenhengster	0.03861066	0.03779567	0.05331883	0.03246504	0.02141882	0.04357104
Holland Black Pied	0.04356257	0.06138847	0.04596957	0.04915268	0.04368586	0.05152196
Andalusian Black	0.04493857	0.04567743	0.06102196	0.04113023	0.03178579	0.05015919
Sweden (Jaemtland or Vatterbotten)	0.06565591	0.06546357	0.07222729	0.04475646	0.06171359	0.08242201
Scottish Highland	0.04829763	0.07476643	0.04170849	0.06387333	0.05730306	0.06035773
Sicilian	0.06061632	0.06446396	0.06666363	0.06666923	0.05768972	0.06361149
Småland	0.05155519	0.06053085	0.06411113	0.03331505	0.04804739	0.06681601
South Devon	0.03054815	0.04513003	0.06232179	0.02969815	0.03065773	0.0478108
Spanish Fighting Cattle	0.05562106	0.07535483	0.04479005	0.06501515	0.05753268	0.06815371
Buchara Grey	0.11345115	0.10569843	0.13509403	0.10461014	0.10286567	0.11207304
Hungarian Grey	0.03100524	0.05425324	0.04912121	0.04845509	0.03571665	0.04227381
Hungarian Grey X Bern Red Pied	0.05893705	0.07841225	0.06839362	0.07641016	0.06734605	0.06233222
Sudeten	0.04003462	0.04696937	0.05421751	0.0351097	0.03701598	0.04596383
Sudeten X Simmental	0.04496237	0.06356471	0.04340722	0.0480045	0.05271679	0.06660909
Sudeten X Tesstal?	0.03981275	0.05631878	0.0576939	0.04833455	0.04327043	0.0497793
Tarentaise	0.04696332	0.06058537	0.04147074	0.05122657	0.046335	0.06086134
Telemark	0.03431855	0.05377897	0.04997393	0.02667697	0.0364832	0.05119719
Tudanca	0.04594533	0.05208502	0.0644971	0.04044464	0.03712596	0.04928874
Tuxer	0.07900308	0.1015944	0.06405662	0.09737494	0.08794526	0.08910063
Heck	0.0310871	0.05887488	0.04378607	0.04013857	0.04247428	0.04813792
Niata	0.20038637	0.22620483	0.18321264	0.22397731	0.21749059	0.20962226
Veredelter Landschlag	0.04756851	0.05685267	0.05986739	0.04717186	0.04631252	0.05994269
Vogtlaender	0.03513325	0.0576769	0.04030343	0.05108186	0.04438415	0.05026706
Waldviertler X Scheinfelder	0.03531828	0.05258861	0.04700253	0.04079254	0.0365365	0.05549489
Watussi	0.04825022	0.04676637	0.06947185	0.04563175	0.03868477	0.05728023
Zebu	0.05654236	0.05812739	0.07847647	0.04459471	0.05185039	0.07334223

Zebu (Africa)	0.06046848	0.06886728	0.0719655	0.05727699	0.05814418	0.07491208
Zebu (Asian)	0.04480694	0.06609164	0.06800851	0.05847066	0.05563374	0.06318753
Zebu (Indian)	0.05612818	0.05293166	0.07948352	0.05355058	0.0490313	0.06334873
Zillertaler	0.05138418	0.07335994	0.04261535	0.06738378	0.05899267	0.06092899

	Simmental	South Moravian Red Pied	Rubia Gallega	Allgaeuer Grey	Krainer Grey	Oberinntaler Grey
Ayrshire	0.04203723	0.04547014	0.07522174	0.05545357	0.04611448	0.032581
Blondvieh: Carinthian	0.03956863	0.026307	0.05409574	0.04439598	0.04395034	0.03012498
Blondvieh: Lavanttaler	0.04274552	0.02916775	0.06027351	0.04626755	0.04715144	0.03180937
Blondvieh: Mariahofer	0.04005688	0.04161196	0.07853502	0.03978667	0.05303002	0.04118202
Blondvieh: Murbodner	0.03774863	0.03034678	0.05309046	0.04617771	0.04035527	0.02900536
Blondvieh: Waldviertler	0.04249473	0.03382951	0.05139284	0.04647115	0.04109348	0.03180227
Bos primigenius (Aurochs)	0.06555693	0.05697777	0.06420744	0.06423542	0.06468882	0.06007611
Brazilian	0.03607501	0.04157918	0.06298685	0.04553115	0.04976058	0.04886537
Montafon	0.03820072	0.02836344	0.04819643	0.04427546	0.04420041	0.02996574
Buša	0.04943872	0.04581382	0.05643811	0.05467211	0.05253288	0.03951443
Chianina	0.05383947	0.04625114	0.05136276	0.06258833	0.04758729	0.05147103
Chillingham	0.06340088	0.0673283	0.08639266	0.06490901	0.07296681	0.05869594
Devon	0.03524452	0.04090541	0.06433497	0.04935956	0.04620313	0.035362
Egerlaender	0.05554776	0.04566856	0.06236963	0.05740989	0.05869029	0.04827201
Hérens	0.04028269	0.05165371	0.08740918	0.05424937	0.05825666	0.04128763
Fjällko	0.05047723	0.04248905	0.05989717	0.05488076	0.05135333	0.03610294
Bern Red Pied	0.03699327	0.02873317	0.05424755	0.04480879	0.04661268	0.02855792
Freiburg Red Pied	0.04446405	0.04473824	0.06343278	0.05035184	0.04616141	0.04551884
Simmental		0.03248331	0.06991845	0.04001385	0.04496943	0.03279607
South Moravian Red Pied	0.03248331		0.06239477	0.0413066	0.04402949	0.03139294
Rubia Gallega	0.06991845	0.06239477		0.07534736	0.06276493	0.06489676
Allgaeuer Grey	0.04001385	0.0413066	0.07534736		0.05541979	0.04398781
Krainer Grey	0.04496943	0.04402949	0.06276493	0.05541979		0.04172244
Oberinntaler Grey	0.03279607	0.03139294	0.06489676	0.04398781	0.04172244	
Haná-Berne	0.047328	0.03567605	0.04950752	0.05195417	0.04653202	0.03760056
Iceland	0.04440294	0.05091408	0.07844427	0.05896701	0.05342344	0.03858968
Jersey	0.05747794	0.06770895	0.10039117	0.06744605	0.06549005	0.06002673

Jersey (polled)	0.05252843	0.06174267	0.09162524	0.06545523	0.05977638	0.05280285
Jutland	0.0593784	0.04722706	0.05317243	0.0611957	0.05461172	0.04628911
Kampeten: Styrian Bergscheck	0.0319722	0.02917475	0.06621696	0.03807557	0.04280747	0.01942265
Kampeten: Welser Schecken	0.05226691	0.04112148	0.05766907	0.05474676	0.05142193	0.04631578
Kerry	0.04336093	0.04170216	0.05531352	0.05545611	0.04681823	0.03217155
Cretan	0.0597982	0.06137867	0.06533979	0.06498243	0.06274728	0.06257159
Kuhlaender	0.03693137	0.02740702	0.05375819	0.04371162	0.04091607	0.02889109
Landschlag von Warnsdorf	0.04697745	0.05095722	0.07577701	0.0544639	0.05678311	0.04956513
Landschlag von Winkelsdorf	0.06359263	0.04805719	0.05649714	0.06091931	0.06366794	0.05708831
Landschlag X Simmental	0.05421554	0.04331337	0.05319598	0.05971052	0.05792519	0.04434978
Limousin	0.0342985	0.04245781	0.07348205	0.04963284	0.04878215	0.04071775
Moravian Landschlag	0.0346816	0.03646457	0.06665358	0.04310574	0.04621363	0.0354874
Normande	0.0461031	0.04650192	0.08324525	0.05801746	0.05372772	0.04596651
North Wales	0.03465225	0.03505966	0.04987875	0.04698628	0.03951016	0.03350329
White Park	0.05696099	0.0638946	0.07813358	0.06649461	0.06919821	0.05675438
Pinzgauer	0.02687786	0.03111707	0.06402558	0.04111658	0.03454639	0.02610268
Pinzgauer X Scottish Highland	0.0606421	0.05211574	0.08571198	0.05650948	0.06400328	0.04871919
Pinzgauer X Simmentaler	0.04706518	0.06113604	0.08935675	0.05939712	0.05658949	0.05158546
Pinzgauer: Moelltaler	0.03683455	0.03062242	0.04878372	0.04537686	0.04277205	0.03707811
Pinzgauer: Pustertaler	0.06234202	0.06578957	0.1016757	0.06322215	0.07202274	0.06214192
Pirenaica	0.03331829	0.03498781	0.06721733	0.04656963	0.03984798	0.03595907
Red Poll	0.05321712	0.05949189	0.09833576	0.05451562	0.0688533	0.06006574
Maas-Rhein-Ijsselschlag	0.03683291	0.03619287	0.06037489	0.04608885	0.04335763	0.03232149
Danish Red	0.05668588	0.04366044	0.05655139	0.05635297	0.05454793	0.04282839
Angeln	0.03460842	0.03920079	0.06499036	0.04537392	0.04782017	0.03474168
Bohemian Red	0.03652585	0.02685293	0.05386946	0.04447369	0.04209026	0.0264805
Polish Red	0.05329366	0.04208847	0.05114621	0.0583835	0.05634692	0.04431474
Sanga	0.08394779	0.07181681	0.05810271	0.08215926	0.08057567	0.07320288
Sardinian	0.06162577	0.05231864	0.0594432	0.06620184	0.05759406	0.05208276
Scheinfelder	0.04787274	0.04778732	0.04974598	0.05824766	0.05630132	0.04741084
Schoenhengster	0.0370482	0.02757921	0.05173099	0.04586641	0.0423131	0.02900026
Holland Black Pied	0.03361768	0.03413945	0.07400418	0.04050474	0.04656117	0.03799367
Andalusian Black	0.04316695	0.03817729	0.04668788	0.05164627	0.05120021	0.03903151

Sweden (Jaemtland or Vatterbotten)	0.07955806	0.06992602	0.08950768	0.07889928	0.08031665	0.05900831
Scottish Highland	0.04031538	0.04895656	0.08087421	0.05045008	0.04589841	0.04790721
Sicilian	0.05693563	0.05239607	0.08687662	0.05506429	0.06211009	0.0557175
Småland	0.06047726	0.0562756	0.05711006	0.06714519	0.06199549	0.04841289
South Devon	0.04712543	0.04078595	0.04716568	0.05457536	0.04773135	0.04283922
Spanish Fighting Cattle	0.04376624	0.04984061	0.08553282	0.05260696	0.06162969	0.04539337
Buchara Grey	0.12141696	0.11001367	0.07781375	0.11899932	0.11684854	0.11694197
Hungarian Grey	0.02980927	0.03457542	0.0630602	0.04539009	0.042535	0.03522193
Hungarian Grey X Bern Red Pied	0.04793391	0.05938711	0.08569337	0.06141236	0.06438066	0.06581193
Sudeten	0.04238462	0.03600175	0.06646598	0.04460194	0.04616449	0.03847859
Sudeten X Simmental	0.05140951	0.0506728	0.08981568	0.05957512	0.05962813	0.04547412
Sudeten X Tesstal?	0.0415195	0.04012172	0.07034706	0.05207571	0.04820728	0.04563898
Tarentaise	0.04114081	0.04461065	0.08004731	0.04939302	0.05530836	0.03303365
Telemark	0.04518199	0.04143559	0.05508099	0.05322942	0.03915911	0.033949
Tudanca	0.04950914	0.03713752	0.05399622	0.05044295	0.04806854	0.04486086
Tuxer	0.06755247	0.07792068	0.11919144	0.07059129	0.08209764	0.0787816
Heck	0.03634379	0.0418108	0.06064637	0.05159659	0.03946429	0.03671476
Niata	0.19246641	0.20687725	0.24610543	0.19928193	0.20303588	0.20515185
Veredelter Landschlag	0.04990193	0.04480491	0.0643776	0.06014574	0.06066889	0.04379805
Vogtlaender	0.03429012	0.04090744	0.08130445	0.04758063	0.04406961	0.03614105
Waldviertler X Scheinfelder	0.04021584	0.04234253	0.0717653	0.04445098	0.05765322	0.03718349
Watussi	0.05610289	0.05100197	0.06057663	0.06209621	0.06255104	0.04507432
Zebu	0.07100937	0.06186849	0.05421053	0.07587165	0.07223729	0.06121353
Zebu (Africa)	0.06513375	0.06485944	0.0640188	0.08113661	0.06915268	0.05672112
Zebu (Asian)	0.05574051	0.0572934	0.07412051	0.06854335	0.06114882	0.05678138
Zebu (Indian)	0.06621872	0.05626586	0.05169998	0.07214186	0.06532857	0.05944954
Zillertaler	0.04042996	0.04941387	0.08991773	0.05162818	0.04975503	0.04831563
	Haná-Berne	Iceland	Jersey	Jersey (polled)	Jutland	Kampeten: Styrian Bergscheck
Ayrshire	0.05479099	0.03321779	0.04928422	0.046877	0.05252131	0.03452227
Blondvieh: Carinthian	0.03136373	0.04926187	0.06706818	0.06347792	0.03576369	0.03052628
Blondvieh: Lavanttaler	0.03645954	0.05361721	0.06685773	0.06498357	0.03892032	0.0285883
Blondvieh: Mariahofer	0.05629895	0.05516607	0.05146604	0.04821187	0.06156031	0.03480781

Blondvieh: Murbodner	0.03378817	0.04886281	0.06482129	0.05943391	0.04390795	0.03219736
Blondvieh: Waldviertler	0.03140024	0.04485787	0.06654353	0.06361918	0.03057168	0.02919254
Bos primigenius (Aurochs)	0.05110096	0.07494792	0.10029968	0.09428409	0.05645617	0.05636559
Brazilian	0.05225589	0.06132485	0.07162385	0.06718752	0.06114146	0.0449613
Montafon	0.03109418	0.05200643	0.07120834	0.06438353	0.03566687	0.02947221
Buša	0.04678487	0.04544503	0.06593598	0.06237141	0.03745221	0.03445063
Chianina	0.04436539	0.06473214	0.07980806	0.07579964	0.04235048	0.05045315
Chillingham	0.07626295	0.05848915	0.07718128	0.07434527	0.06733402	0.05569062
Devon	0.04853142	0.04073393	0.04939675	0.04773388	0.0512945	0.03386158
Egerlaender	0.04165455	0.07089616	0.08016218	0.07940183	0.05365359	0.04763202
Hérens	0.06518531	0.04192558	0.05021225	0.05118105	0.06789287	0.04010875
Fjällko	0.04026387	0.04122555	0.06330574	0.06371847	0.03139728	0.03653201
Bern Red Pied	0.0320187	0.05211784	0.06979548	0.06533333	0.04022344	0.02885536
Freiburg Red Pied	0.04405213	0.0668104	0.0756177	0.0738197	0.05836319	0.04946639
Simmental	0.047328	0.04440294	0.05747794	0.05252843	0.0593784	0.0319722
South Moravian Red Pied	0.03567605	0.05091408	0.06770895	0.06174267	0.04722706	0.02917475
Rubia Gallega	0.04950752	0.07844427	0.10039117	0.09162524	0.05317243	0.06621696
Allgaeuer Grey	0.05195417	0.05896701	0.06744605	0.06545523	0.0611957	0.03807557
Krainer Grey	0.04653202	0.05342344	0.06549005	0.05977638	0.05461172	0.04280747
Oberinntaler Grey	0.03760056	0.03858968	0.06002673	0.05280285	0.04628911	0.01942265
Haná-Berne		0.05762333	0.08041276	0.07577391	0.04125391	0.0403325
Iceland	0.05762333		0.05395115	0.04955065	0.05557115	0.04121266
Jersey	0.08041276	0.05395115		0.04477592	0.08109701	0.0596893
Jersey (polled)	0.07577391	0.04955065	0.04477592		0.08008478	0.0541005
Jutland	0.04125391	0.05557115	0.08109701	0.08008478		0.04515243
Kampeten: Styrian Bergscheck	0.0403325	0.04121266	0.0596893	0.0541005	0.04515243	
Kampeten: Welser Schecken	0.03964303	0.0675558	0.09042115	0.08006016	0.04922001	0.0446258
Kerry	0.04224705	0.03557467	0.06452465	0.05944948	0.0345072	0.03333158
Cretan	0.06480959	0.06221684	0.06900924	0.06995088	0.05747061	0.05743647
Kuhlaender	0.0297461	0.04495938	0.06384276	0.06003122	0.03763203	0.02874163
Landschlag von Warnsdorf	0.05706364	0.05290068	0.05087011	0.05952562	0.06344136	0.04827437
Landschlag von Winkelsdorf	0.04275357	0.07048797	0.09018616	0.08867748	0.04526864	0.05443987
Landschlag X Simmental	0.0432666	0.06262222	0.0779825	0.07362043	0.04807697	0.04481601

Limousin	0.05479167	0.04640614	0.04823267	0.04557587	0.0613957	0.04280084
Moravian Landschlag	0.04664381	0.04253928	0.05621417	0.05118641	0.05376107	0.03534489
Normande	0.06315644	0.05618198	0.04786025	0.04645562	0.06470313	0.0458265
North Wales	0.03766059	0.04400875	0.06155026	0.05856947	0.03799744	0.03370327
White Park	0.06475632	0.04549914	0.06159976	0.06350438	0.06049017	0.05358801
Pinzgauer	0.04221156	0.04164893	0.05163709	0.04531043	0.05185175	0.02880622
Pinzgauer X Scottish Highland	0.06252783	0.07320131	0.08006469	0.07311459	0.07023809	0.04700205
Pinzgauer X Simmentaler	0.07033828	0.05759352	0.04926376	0.05281189	0.07831955	0.05353538
Pinzgauer: Moelltaler	0.03253071	0.05537067	0.07142076	0.06606348	0.04341386	0.03608506
Pinzgauer: Pustertaler	0.07870346	0.08115105	0.08253387	0.0728746	0.08812317	0.0575155
Pirenaica	0.05021974	0.04065813	0.05070007	0.04732888	0.05128235	0.03392058
Red Poll	0.07859308	0.06290673	0.06866104	0.06130313	0.08360647	0.05325459
Maas-Rhein-Ijsselschlag	0.04173428	0.03964265	0.05298141	0.04964024	0.04010082	0.03135541
Danish Red	0.04010774	0.05683018	0.07187753	0.07083687	0.03527052	0.04287763
Angeln	0.04926493	0.04276307	0.05929726	0.05439034	0.05109173	0.03329575
Bohemian Red	0.02921202	0.04453882	0.06260418	0.05896749	0.03769888	0.02735386
Polish Red	0.03999138	0.05192137	0.07317966	0.07212309	0.03359346	0.0418578
Sanga	0.0629596	0.08486337	0.10582867	0.10119549	0.05599992	0.07031523
Sardinian	0.05114948	0.06645599	0.0820912	0.07821579	0.0485175	0.05115351
Scheinfelder	0.0444436	0.05756424	0.07859519	0.07058914	0.05170265	0.04630128
Schoenhengster	0.02795632	0.04796075	0.06988825	0.06464261	0.03806388	0.02961409
Holland Black Pied	0.04812529	0.04846206	0.06424892	0.05785026	0.05718328	0.03364204
Andalusian Black	0.03461249	0.05590338	0.08187606	0.07453949	0.03996591	0.03481305
Sweden (Jaemtland or Vatterbotten)	0.06898383	0.06475345	0.07884687	0.08268069	0.05985098	0.06175126
Scottish Highland	0.06439453	0.05233039	0.06021796	0.05143257	0.07113895	0.04563605
Sicilian	0.06284305	0.07181814	0.07180891	0.07607262	0.0736051	0.05323996
Småland	0.0473726	0.04807235	0.07733335	0.07566845	0.03770982	0.05167689
South Devon	0.03883264	0.05164277	0.06699462	0.06527367	0.03726812	0.04271661
Spanish Fighting Cattle	0.06676728	0.05591001	0.0709312	0.06358493	0.07119718	0.04021988
Buchara Grey	0.10071729	0.12554845	0.14876288	0.14365432	0.09107113	0.11452085
Hungarian Grey	0.04682392	0.04921226	0.05931665	0.05410085	0.05560958	0.03814438
Hungarian Grey X Bern Red Pied	0.06865983	0.07174674	0.07540957	0.07411331	0.08184993	0.06463858
Sudeten	0.03908216	0.04886451	0.05904119	0.06497399	0.04498254	0.03769155

Sudeten X Simmental	0.06491073	0.04908897	0.04290894	0.05211355	0.06633238	0.04598858
Sudeten X Tesstal?	0.04798867	0.0529987	0.06248188	0.06275583	0.05664633	0.04493601
Tarentaise	0.05614359	0.04803316	0.06009782	0.05632126	0.05838768	0.0287234
Telemark	0.03833616	0.03981323	0.06171403	0.05882304	0.03366613	0.03575799
Tudanca	0.04191334	0.0598226	0.07695607	0.07425541	0.03721381	0.04135579
Tuxer	0.09942955	0.08452961	0.0769015	0.06932441	0.1062414	0.07269224
Heck	0.04590395	0.0380076	0.05561512	0.05492696	0.04715603	0.03764615
Niata	0.22771185	0.20193559	0.17428497	0.17558512	0.23651907	0.20448827
Veredelter Landschlag	0.04404392	0.04936592	0.07154265	0.07008359	0.05266474	0.04614728
Vogtlaender	0.05478804	0.0458232	0.04344124	0.04471132	0.06470462	0.03728852
Waldviertler X Scheinfelder	0.05143832	0.04807461	0.06110581	0.05867523	0.05312331	0.03407778
Watussi	0.04555523	0.06095363	0.08012647	0.07529861	0.05389136	0.04680642
Zebu	0.05628593	0.06746473	0.09127122	0.08722718	0.04534552	0.05905037
Zebu (Africa)	0.05678896	0.05942589	0.08944247	0.08148819	0.0625783	0.0608519
Zebu (Asian)	0.06212372	0.06063769	0.06469653	0.06575601	0.06334888	0.05735414
Zebu (Indian)	0.05246812	0.07422879	0.09250924	0.08551404	0.04938252	0.05769999
Zillertaler	0.06701203	0.05893648	0.05527222	0.04856614	0.0751891	0.0453463

	Kampeten: Welser Schecken	Kerry	Cretan	Kuhlaender	Landschlag von Warnsdorf	Landschlag von Winkelsdorf
Ayrshire	0.06231373	0.03543689	0.06436702	0.04140128	0.0528842	0.06853204
Blondvieh: Carinthian	0.04616969	0.0334083	0.05907785	0.0196602	0.04731202	0.04062382
Blondvieh: Lavanttaler	0.04741743	0.03780142	0.05543973	0.02642788	0.05184264	0.04369393
Blondvieh: Mariahofer	0.05933208	0.05207182	0.05651424	0.04186169	0.04941892	0.0661983
Blondvieh: Murbodner	0.04848935	0.03785828	0.05990687	0.02130145	0.044865	0.04857035
Blondvieh: Waldviertler	0.04409933	0.02672226	0.04920387	0.02152577	0.05012264	0.04155981
Bos primigenius (Aurochs)	0.04363695	0.06052267	0.07698265	0.05911853	0.08587268	0.06293015
Brazilian	0.05537262	0.05348561	0.05665569	0.04577396	0.05651841	0.06416328
Montafon	0.04240493	0.03175527	0.05369294	0.02328545	0.05175198	0.04324698
Buša	0.05506862	0.02623326	0.04068807	0.03609398	0.05128729	0.05188176
Chianina	0.04880736	0.04591078	0.04943993	0.04028807	0.06589155	0.05040062
Chillingham	0.07390826	0.05522087	0.06769895	0.06562845	0.07724012	0.08337797
Devon	0.06078245	0.0343283	0.04924526	0.03221987	0.03947961	0.06126889
Egerlaender	0.05813884	0.05538626	0.06927349	0.04003985	0.05730359	0.04920365

Hérens	0.07154436	0.04805885	0.06706324	0.05206984	0.05094791	0.08036907
Fjällko	0.05565752	0.02423758	0.05379833	0.03213261	0.047944	0.04662542
Bern Red Pied	0.04449527	0.03594035	0.05992822	0.0240652	0.0495598	0.04304889
Freiburg Red Pied	0.05218958	0.05621864	0.06971282	0.04434161	0.06028473	0.06114716
Simmental	0.05226691	0.04336093	0.0597982	0.03693137	0.04697745	0.06359263
South Moravian Red Pied	0.04112148	0.04170216	0.06137867	0.02740702	0.05095722	0.04805719
Rubia Gallega	0.05766907	0.05531352	0.06533979	0.05375819	0.07577701	0.05649714
Allgaeuer Grey	0.05474676	0.05545611	0.06498243	0.04371162	0.0544639	0.06091931
Krainer Grey	0.05142193	0.04681823	0.06274728	0.04091607	0.05678311	0.06366794
Oberinntaler Grey	0.04631578	0.03217155	0.06257159	0.02889109	0.04956513	0.05708831
Haná-Berne	0.03964303	0.04224705	0.06480959	0.0297461	0.05706364	0.04275357
Iceland	0.0675558	0.03557467	0.06221684	0.04495938	0.05290068	0.07048797
Jersey	0.09042115	0.06452465	0.06900924	0.06384276	0.05087011	0.09018616
Jersey (polled)	0.08006016	0.05944948	0.06995088	0.06003122	0.05952562	
Jutland	0.04922001	0.0345072	0.05747061	0.03763203	0.06344136	0.04526864
Kampeten: Styrian Bergscheck	0.0446258	0.03333158	0.05743647	0.02874163	0.04827437	0.05443987
Kampeten: Welser Schecken		0.05006288	0.0698326	0.0437112	0.07465328	0.05646329
Kerry	0.05006288		0.05178796	0.03350378	0.05332254	0.05446085
Cretan	0.0698326	0.05178796		0.05238149	0.05922395	0.06458369
Kuhlaender	0.0437112	0.03350378	0.05238149		0.04515006	0.04264283
Landschlag von Warnsdorf	0.07465328	0.05332254	0.05922395	0.04515006		0.06653157
Landschlag von Winkelsdorf	0.05646329	0.05446085	0.06458369	0.04264283	0.06653157	
Landschlag X Simmental	0.0579148	0.04545557	0.06228238	0.03381894	0.05828286	0.04215622
Limousin	0.06474193	0.04786801	0.05944172	0.03823029	0.04487756	0.06762615
Moravian Landschlag	0.05356226	0.04093789	0.05670386	0.03098916	0.04386363	0.05980261
Normande	0.06876686	0.05372748	0.06706652	0.04679858	0.05778615	0.07351399
North Wales	0.04933214	0.02925481	0.04538394	0.02438523	0.04534039	0.0509614
White Park	0.07616929	0.04517886	0.05241183	0.05522999	0.05225762	0.07437653
Pinzgauer	0.05134775	0.03811471	0.05643078	0.02726769	0.04119607	0.05855491
Pinzgauer X Scottish Highland	0.06429105	0.06745119	0.08625902	0.05532467	0.07118902	0.07491505
Pinzgauer X Simmentaler	0.0782341	0.06333374	0.07392009	0.05575164	0.05349487	0.08816221
Pinzgauer: Moelltaler	0.04334726	0.04016553	0.05018084	0.02510078	0.04940864	0.04583055
Pinzgauer: Pustertaler	0.07293351	0.07985107	0.08677649	0.07095016	0.08375789	0.09375115

Pirenaica	0.05566547	0.03699828	0.050906	0.03455614	0.04698681	0.06102273
Red Poll	0.07432893	0.0672097	0.078743	0.06766193	0.06792455	0.08946096
Maas-Rhein-Ijsselschlag	0.05403745	0.02873205	0.0460416	0.02515488	0.04308049	0.05257455
Danish Red	0.05549252	0.03812506	0.05743507	0.03364597	0.05611851	0.04272137
Angeln	0.05286352	0.03254057	0.05194902	0.0367334	0.04875098	0.06074806
Bohemian Red	0.04399155	0.02967113	0.05329684	0.01826221	0.04309572	0.04144209
Polish Red	0.05397461	0.03337658	0.0435946	0.02947076	0.05158207	0.03973129
Sanga	0.07463999	0.06626136	0.06704099	0.06398647	0.08292995	0.05440583
Sardinian	0.06193428	0.05367393	0.06511637	0.04439664	0.06858212	0.05052835
Scheinfelder	0.0536904	0.04391415	0.05322662	0.0401564	0.05575566	0.05505139
Schoenhengster	0.04158083	0.03275056	0.05667081	0.02014905	0.04845552	0.042032
Holland Black Pied	0.04679773	0.04387589	0.06156205	0.04152582	0.05607511	0.06320691
Andalusian Black	0.03956974	0.0347805	0.05608263	0.03663776	0.06106568	0.0463464
Sweden (Jaemtland or Vatterbotten)	0.08177579	0.05788031	0.08584366	0.06312248	0.0737639	0.07297514
Scottish Highland	0.06603566	0.05439381	0.07111706	0.05371469	0.05666859	0.08221077
Sicilian	0.07059644	0.0699507	0.08609028	0.057332	0.06360661	0.07493262
Småland	0.06217619	0.03206047	0.05506278	0.04295178	0.06365999	0.05470054
South Devon	0.05573941	0.03315721	0.0489089	0.029045	0.04886902	0.04484036
Spanish Fighting Cattle	0.0601383	0.0532241	0.07616286	0.05907887	0.06884454	0.08032679
Buchara Grey	0.09987045	0.104018	0.09399417	0.10202659	0.12462814	0.09094893
Hungarian Grey	0.0545016	0.04400028	0.05803786	0.03473993	0.04759919	0.06066596
Hungarian Grey X Bern Red Pied	0.07035255	0.07040254	0.07716042	0.06316913	0.06659815	0.08366749
Sudeten	0.05660971	0.04159442	0.05817963	0.03012961	0.0449372	0.04881196
Sudeten X Simmental	0.07636512	0.05272112	0.06953812	0.0502899	0.05183536	0.0719206
Sudeten X Tesstal?	0.0588225	0.04784745	0.06459204	0.04158298	0.04577238	0.06441986
Tarentaise	0.05812826	0.04496781	0.06464466	0.04419816	0.05952195	0.06796265
Telemark	0.04840666	0.02436683	0.05060085	0.02959791	0.05067832	0.05366125
Tudanca	0.04518263	0.04309677	0.05122573	0.03618714	0.05711422	0.0471081
Tuxer	0.09322267	0.09162134	0.09726794	0.08569177	0.08475512	0.10906038
Heck	0.0561721	0.0321467	0.04932887	0.03612828	0.04432479	0.06315743
Niata	0.22708997	0.21899999	0.21617914	0.21367789	0.19815463	0.24138393
Veredelter Landschlag	0.06044118	0.04632448	0.06071678	0.03701564	0.05631096	0.05225479
Vogtlaender	0.06670175	0.05086289	0.06878152	0.0404372	0.04341149	0.06962594

Waldviertler X Scheinfelder	0.06166055	0.043467	0.05991302	0.03738192	0.04663095	0.05739888
Watussi	0.06185824	0.04989997	0.0726784	0.04360306	0.06013102	0.05675341
Zebu	0.06609835	0.04795029	0.05173739	0.05105565	0.07033085	0.05101402
Zebu (Africa)	0.06853898	0.05119627	0.06799722	0.05591948	0.07193207	0.06971519
Zebu (Asian)	0.06707126	0.05798711	0.05578213	0.04848799	0.06349601	0.07177995
Zebu (Indian)	0.05630159	0.05337604	0.05422269	0.04699666	0.07119827	0.05289867
Zillertaler	0.06718179	0.06235916	0.07174291	0.05412765	0.0617426	0.08288851

	Landschlag X Simmental	Limousin	Moravian Landschlag	Normande	North Wales	White Park
Ayrshire	0.05672837	0.04035789	0.04323182	0.03961834	0.04038236	0.05521112
Blondvieh: Carinthian	0.03404019	0.04044946	0.0359338	0.04561506	0.02739349	0.05929033
Blondvieh: Lavanttaler	0.03665635	0.04491207	0.04337642	0.04440367	0.03253143	0.05850102
Blondvieh: Mariahofer	0.05556613	0.04004022	0.04178116	0.03845904	0.04327838	0.05805194
Blondvieh: Murbodner	0.03473488	0.03542762	0.03433548	0.04632123	0.02759978	0.06182317
Blondvieh: Waldviertler	0.03952114	0.04769658	0.03750949	0.05309553	0.02503625	0.04958168
Bos primigenius (Aurochs)	0.06813722	0.07934974	0.07032274	0.08369193	0.06178933	0.08362061
Brazilian	0.0573605	0.04536744	0.04863008	0.05720895	0.04062687	0.07021076
Montafon	0.03331943	0.04425001	0.03860797	0.0499145	0.02678434	0.05817668
Buša	0.04476709	0.05352391	0.04522441	0.05962417	0.03453965	0.04136405
Chianina	0.05242066	0.0593372	0.05531119	0.06135516	0.0387315	0.06793738
Chillingham	0.07420916	0.07514389	0.06655889	0.07664625	0.05843507	0.06035397
Devon	0.0474073	0.02915565	0.03552862	0.03623937	0.0270173	0.04705943
Egerlaender	0.0404866	0.05600788	0.05608825	0.05849572	0.04869071	0.07054329
Hérens	0.06693832	0.0498028	0.04898589	0.05300083	0.04897482	0.05064562
Fjällko	0.04446289	0.04902054	0.04361315	0.05290015	0.03466578	0.04481568
Bern Red Pied	0.0340922	0.04274113	0.04059085	0.04794351	0.03002464	0.06074582
Freiburg Red Pied	0.05948201	0.05024509	0.05060705	0.058522	0.04371414	0.07599673
Simmental	0.05421554	0.0342985	0.0346816	0.0461031	0.03465225	0.05696099
South Moravian Red Pied	0.04331337	0.04245781	0.03646457	0.04650192	0.03505966	0.0638946
Rubia Gallega	0.05319598	0.07348205	0.06665358	0.08324525	0.04987875	0.07813358
Allgaeuer Grey	0.05971052	0.04963284	0.04310574	0.05801746	0.04698628	0.06649461
Krainer Grey	0.05792519	0.04878215	0.04621363	0.05372772	0.03951016	0.06919821
Oberinntaler Grey	0.04434978	0.04071775	0.0354874	0.04596651	0.03350329	0.05675438

Haná-Berne	0.0432666	0.05479167	0.04664381	0.06315644	0.03766059	0.06475632
Iceland	0.06262222	0.04640614	0.04253928	0.05618198	0.04400875	0.04549914
Jersey	0.0779825	0.04823267	0.05621417	0.04786025	0.06155026	0.06159976
Jersey (polled)	0.07362043	0.04557587	0.05118641	0.04645562	0.05856947	0.06350438
Jutland	0.04807697	0.0613957	0.05376107	0.06470313	0.03799744	0.06049017
Kampeten: Styrian Bergscheck	0.04481601	0.04280084	0.03534489	0.0458265	0.03370327	0.05358801
Kampeten: Welser Schecken	0.0579148	0.06474193	0.05356226	0.06876686	0.04933214	0.07616929
Kerry	0.04545557	0.04786801	0.04093789	0.05372748	0.02925481	0.04517886
Cretan	0.06228238	0.05944172	0.05670386	0.06706652	0.04538394	0.05241183
Kuhlaender	0.03381894	0.03823029	0.03098916	0.04679858	0.02438523	0.05522999
Landschlag von Warnsdorf	0.05828286	0.04487756	0.04386363	0.05778615	0.04534039	0.05225762
Landschlag von Winkelsdorf	0.04215622	0.06762615	0.05980261	0.07351399	0.0509614	0.07437653
Landschlag X Simmental		0.05485403	0.04968481	0.05965951	0.04035817	0.06855405
Limousin	0.05485403		0.03458237	0.03358822	0.0368046	0.06083747
Moravian Landschlag	0.04968481	0.03458237		0.04998058	0.03577754	0.05521437
Normande	0.05965951	0.03358822	0.04998058		0.04674399	0.06659138
North Wales	0.04035817	0.0368046	0.03577754	0.04674399		0.0495452
White Park	0.06855405	0.06083747	0.05521437	0.06659138	0.0495452	
Pinzgauer	0.04629777	0.02728767	0.02831836	0.03777846	0.02813222	0.05742625
Pinzgauer X Scottish Highland	0.06041817	0.0621978	0.06070918	0.05830927	0.06253771	0.08280509
Pinzgauer X Simmentaler	0.07317865	0.04441211	0.05042168	0.05335417	0.05335208	0.06814936
Pinzgauer: Moelltaler	0.03865333	0.04217654	0.03872308	0.05214954	0.02591048	0.06088525
Pinzgauer: Pustertaler	0.08198472	0.07285477	0.07244499	0.06914251	0.07321797	0.09022956
Pirenaica	0.05296783	0.03377919	0.03641919	0.03651274	0.03005756	0.05373973
Red Poll	0.08110903	0.06572756	0.06036764	0.06828162	0.06701808	0.07532988
Maas-Rhein-Ijsselschlag	0.04359965	0.03228804	0.03249807	0.0377044	0.02429076	0.04358141
Danish Red	0.03915675	0.05210983	0.05020875	0.05158834	0.03689056	0.05794972
Angeln	0.04930962	0.04555041	0.040199	0.05239516	0.03470004	0.0496329
Bohemian Red	0.03477905	0.04018409	0.0315165	0.04812367	0.02765865	0.05238122
Polish Red	0.03507863	0.05488151	0.04412096	0.06198353	0.03294871	0.05026187
Sanga	0.06036257	0.08657005	0.08050437	0.08807763	0.06390713	0.07486165
Sardinian	0.04683201	0.05810537	0.06075221	0.05598211	0.04697042	0.07240147
Scheinfelder	0.04659184	0.05367366	0.0500287	0.0643773	0.0380524	0.05421514

Schoenhengster	0.03531604	0.04458719	0.03487123	0.05301329	0.0291787	0.05598689
Holland Black Pied	0.06094948	0.04692862	0.03881497	0.05138478	0.0429289	0.05973898
Andalusian Black	0.04255433	0.05459045	0.04864471	0.06250334	0.03487307	0.05887317
Sweden (Jaemtland or Vatterbotten)	0.06896254	0.0741499	0.07173518	0.0700175	0.07023695	0.06838392
Scottish Highland	0.07063856	0.04920176	0.04742141	0.05656646	0.04911508	0.06827201
Sicilian	0.06977608	0.05995568	0.05885101	0.0615586	0.06511929	0.08432552
Småland	0.04986992	0.06170473	0.05533895	0.06917016	0.04083429	0.04918777
South Devon	0.03966414	0.0438779	0.04193588	0.04988292	0.02716794	0.05425143
Spanish Fighting Cattle	0.06956781	0.05885674	0.05501067	0.06145599	0.0572226	0.07014671
Buchara Grey	0.09779348	0.12407691	0.11539933	0.1306243	0.09911153	0.1156078
Hungarian Grey	0.05009317	0.02876841	0.03653543	0.04213248	0.02980069	0.0642237
Hungarian Grey X Bern Red Pied	0.08134647	0.05180322	0.05759004	0.06721642	0.06096665	0.08331393
Sudeten	0.04850385	0.04528251	0.03632585	0.05293464	0.03694305	0.05540987
Sudeten X Simmental	0.06138916	0.04693223	0.05161578	0.0397019	0.0515475	0.05722837
Sudeten X Tesstal?	0.05654279	0.04176672	0.03934717	0.05215655	0.04222244	0.06266122
Tarentaise	0.05606982	0.0510784	0.0464058	0.0507449	0.04696258	0.05754494
Telemark	0.0472139	0.04661896	0.04136634	0.05156046	0.02645853	0.04717776
Tudanca	0.04821708	0.05558279	0.0486542	0.06103337	0.03655916	0.0637195
Tuxer	0.10049797	0.07589794	0.07538204	0.07659845	0.08578937	0.09479388
Heck	0.05497694	0.04100489	0.03973018	0.05157877	0.02507081	0.04638604
Niata	0.22641364	0.18976865	0.19943347	0.19025759	0.21148885	0.21498226
Veredelter Landschlag	0.04776374	0.04942809	0.04285264	0.06242511	0.04132526	0.05913685
Vogtlaender	0.0583015	0.02863936	0.03638792	0.03534051	0.04283257	0.06336112
Waldviertler X Scheinfelder	0.05033356	0.04059151	0.04218459	0.04648631	0.03607662	0.04954937
Watussi	0.05047159	0.05644954	0.05259734	0.06172016	0.04967788	0.06605886
Zebu	0.05208427	0.07223318	0.06470773	0.07575571	0.05084178	0.05759544
Zebu (Africa)	0.05983607	0.06498749	0.06227668	0.07881741	0.05582075	0.06802198
Zebu (Asian)	0.06287692	0.04615196	0.05334081	0.05162916	0.04857515	0.06782318
Zebu (Indian)	0.04876466	0.06872085	0.06162309	0.07088812	0.04937395	0.06864225
Zillertaler	0.07227381	0.04654102	0.05127947	0.04719363	0.0537662	0.07242997

	Pinzgauer	Pinzgauer X Scottish Highland	Pinzgauer X Simmental	Pinzgauer: Moelltaler	Pinzgauer: Pustertaler	Pirenaica
Ayrshire	0.03504277	0.05902845	0.05074525	0.05108962	0.071816	0.03331536

Blondvieh: Carinthian	0.02943521	0.0543728	0.06152537	0.02744421	0.07532121	0.03506909
Blondvieh: Lavanttaler	0.03574653	0.05143479	0.06271501	0.03224799	0.07106856	0.03885921
Blondvieh: Mariahofer	0.03651396	0.05309988	0.05327875	0.04466143	0.05869017	0.03620295
Blondvieh: Murbodner	0.02446642	0.04757542	0.05349779	0.0274293	0.07238881	0.03839296
Blondvieh: Waldviertler	0.03428901	0.06078693	0.06148398	0.0300203	0.07453269	0.03573131
Bos primigenius (Aurochs)	0.06740743	0.07356953	0.08858689	0.05960256	0.08438199	0.07086095
Brazilian	0.04154334	0.06549124	0.06125236	0.03970387	0.06807053	0.04327476
Montafon	0.03126671	0.05327952	0.06332775	0.0237834	0.07111485	0.03825494
Buša	0.0444649	0.06545919	0.06741579	0.0419312	0.07848648	0.04248067
Chianina	0.04634518	0.07465066	0.07290662	0.03491606	0.08225006	0.04934593
Chillingham	0.06476089	0.07751943	0.07522347	0.06915359	0.0775206	0.06056365
Devon	0.02517862	0.06033746	0.04708433	0.0349088	0.07254632	0.02971606
Egerlaender	0.05047027	0.0560804	0.07421976	0.04165657	0.08304038	0.05681196
Hérens	0.04365916	0.06240813	0.04511493	0.05957862	0.06583582	0.04515009
Fjällko	0.0412989	0.06625988	0.0675096	0.04145451	0.0843537	0.04157507
Bern Red Pied	0.03098035	0.05032733	0.06092176	0.02534533	0.072208	0.03951584
Freiburg Red Pied	0.03919278	0.0658261	0.05902019	0.03892514	0.0794709	0.04966014
Simmental	0.02687786	0.0606421	0.04706518	0.03683455	0.06234202	0.03331829
South Moravian Red Pied	0.03111707	0.05211574	0.06113604	0.03062242	0.06578957	0.03498781
Rubia Gallega	0.06402558	0.08571198	0.08935675	0.04878372	0.1016757	0.06721733
Allgaeuer Grey	0.04111658	0.05650948	0.05939712	0.04537686	0.06322215	0.04656963
Krainer Grey	0.03454639	0.06400328	0.05658949	0.04277205	0.07202274	0.03984798
Oberinntaler Grey	0.02610268	0.04871919	0.05158546	0.03707811	0.06214192	0.03595907
Haná-Berne	0.04221156	0.06252783	0.07033828	0.03253071	0.07870346	0.05021974
Iceland	0.04164893	0.07320131	0.05759352	0.05537067	0.08115105	0.04065813
Jersey	0.05163709	0.08006469	0.04926376	0.07142076	0.08253387	0.05070007
Jersey (polled)	0.04531043	0.07311459	0.05281189	0.06606348	0.0728746	0.04732888
Jutland	0.05185175	0.07023809	0.07831955	0.04341386	0.08812317	0.05128235
Kampeten: Styrian Bergscheck	0.02880622	0.04700205	0.05353538	0.03608506	0.0575155	0.03392058
Kampeten: Welser Schecken	0.05134775	0.06429105	0.0782341	0.04334726	0.07293351	0.05566547
Kerry	0.03811471	0.06745119	0.06333374	0.04016553	0.07985107	0.03699828
Cretan	0.05643078	0.08625902	0.07392009	0.05018084	0.08677649	0.050906
Kuhlaender	0.02726769	0.05532467	0.05575164	0.02510078	0.07095016	0.03455614

Landschlag von Warnsdorf	0.04119607	0.07118902	0.05349487	0.04940864	0.08375789	0.04698681
Landschlag von Winkelsdorf	0.05855491	0.07491505	0.08816221	0.04583055	0.09375115	0.06102273
Landschlag X Simmental	0.04629777	0.06041817	0.07317865	0.03865333	0.08198472	0.05296783
Limousin	0.02728767	0.0621978	0.04441211	0.04217654	0.07285477	0.03377919
Moravian Landschlag	0.02831836	0.06070918	0.05042168	0.03872308	0.07244499	0.03641919
Normande	0.03777846	0.05830927	0.05335417	0.05214954	0.06914251	0.03651274
North Wales	0.02813222	0.06253771	0.05335208	0.02591048	0.07321797	0.03005756
White Park	0.05742625	0.08280509	0.06814936	0.06088525	0.09022956	0.05373973
Pinzgauer		0.05309714	0.04119425	0.03094287	0.06414774	0.02911083
Pinzgauer X Scottish Highland	0.05309714		0.06686611	0.05887539	0.06244211	0.06286391
Pinzgauer X Simmentaler	0.04119425	0.06686611		0.06066189	0.07120599	0.05389686
Pinzgauer: Moelltaler	0.03094287	0.05887539	0.06066189		0.07324698	0.03880213
Pinzgauer: Pustertaler	0.06414774	0.06244211	0.07120599	0.07324698		0.06912696
Pirenaica	0.02911083	0.06286391	0.05389686	0.03880213	0.06912696	
Red Poll	0.05924695	0.0710677	0.06957473	0.06782858	0.06576335	0.05594206
Maas-Rhein-Ijsselschlag	0.02807652	0.05846094	0.0511397	0.03359132	0.07207902	0.03012762
Danish Red	0.04602298	0.06061487	0.07276434	0.03952159	0.08581565	0.04508088
Angeln	0.03545422	0.06135539	0.05195526	0.04057061	0.07077925	0.04052535
Bohemian Red	0.02776795	0.05520379	0.05769684	0.02783787	0.07491304	0.03565174
Polish Red	0.04664623	0.07086601	0.07341386	0.0350655	0.08549116	0.0460796
Sanga	0.07833644	0.08607199	0.10524062	0.06271317	0.10467708	0.07640032
Sardinian	0.05241603	0.06674897	0.07926907	0.04657539	0.08485139	0.05188059
Scheinfelder	0.04763194	0.07104821	0.06855785	0.04035863	0.08362055	0.05287014
Schoenhengster	0.03092533	0.05619463	0.06055135	0.02653097	0.07464911	0.0404013
Holland Black Pied	0.03854044	0.05939902	0.05950668	0.04264106	0.06497973	0.0379182
Andalusian Black	0.04544181	0.06231355	0.07303943	0.03430945	0.07608	0.04639594
Sweden (Jaemtland or Vatterbotten)	0.07047662	0.07334647	0.08611379	0.07524387	0.10390869	0.07164333
Scottish Highland	0.04041485	0.06221807	0.0483736	0.05470747	0.06547776	0.04338389
Sicilian	0.05334207	0.05758767	0.06482116	0.0615476	0.07830055	0.06360318
Småland	0.05506338	0.08044594	0.07660906	0.05205178	0.09363195	0.05321025
South Devon	0.03606375	0.06756454	0.06469213	0.02950064	0.08485617	0.03971189
Spanish Fighting Cattle	0.05221629	0.06231095	0.06544877	0.06177479	0.06233908	0.05040342
Buchara Grey	0.11751627	0.12934227	0.14131583	0.0932288	0.14487604	0.11492516

Hungarian Grey	0.02550828	0.05944639	0.04863163	0.03294734	0.06904434	0.03422522
Hungarian Grey X Bern Red Pied	0.05625353	0.08347551	0.06537279	0.05997098	0.08216507	0.05770602
Sudeten	0.03580536	0.06210365	0.05771147	0.03987367	0.07831474	0.03945733
Sudeten X Simmental	0.04473946	0.06415043	0.05510306	0.0600082	0.07743803	0.04455174
Sudeten X Tesstal?	0.03760322	0.0667908	0.06088003	0.04027869	0.08315607	0.04198729
Tarentaise	0.04307757	0.0546689	0.05564042	0.05170263	0.06077526	0.04234276
Telemark	0.03455284	0.06360775	0.05636496	0.03677348	0.07718721	0.03686007
Tudanca	0.04390969	0.06303912	0.07170912	0.03034898	0.07583583	0.04509081
Tuxer	0.07246003	0.07813122	0.06809716	0.08751029	0.06420203	0.07556486
Heck	0.0323122	0.06668611	0.05100621	0.03824009	0.07542103	0.0293085
Niata	0.19465177	0.20390199	0.17358481	0.21494517	0.18533463	0.19881641
Veredelter Landschlag	0.04608973	0.07029871	0.06475692	0.0458069	0.08439979	0.05151258
Vogtlaender	0.02692809	0.05539606	0.03835895	0.04956436	0.06595957	0.0352773
Waldviertler X Scheinfelder	0.03902701	0.05932752	0.05457793	0.04449626	0.07031532	0.03889479
Watussi	0.05078905	0.06268249	0.07334531	0.04722149	0.08974922	0.05648177
Zebu	0.06509787	0.08309298	0.09164552	0.05216186	0.10014325	0.06274648
Zebu (Africa)	0.06255126	0.08504145	0.08139099	0.05954109	0.0982495	0.06699976
Zebu (Asian)	0.04881184	0.07478445	0.06364088	0.0510689	0.08152895	0.05018705
Zebu (Indian)	0.05997134	0.07817723	0.08650298	0.04310434	0.09269184	0.06021756
Zillertaler	0.04125113	0.06042988	0.0439769	0.05784772	0.04884842	0.04449393

	Red Poll	Maas-Rhein-Ijsselschlag	Danish Red	Angeln	Bohemian Red	Polish Red
Ayrshire	0.06336932	0.03308227	0.04911076	0.04292993	0.04091239	0.0534471
Blondvieh: Carinthian	0.06967291	0.02704046	0.02799221	0.04095237	0.01878645	0.03277301
Blondvieh: Lavanttaler	0.07033559	0.03088575	0.02965098	0.04036793	0.0242754	0.03553664
Blondvieh: Mariahofer	0.05256456	0.03717815	0.05110631	0.04481188	0.04297238	0.05468765
Blondvieh: Murbodner	0.06905703	0.02980659	0.03774435	0.03814	0.02254212	0.03889612
Blondvieh: Waldviertler	0.06988514	0.02650736	0.02978659	0.03777714	0.02046472	0.02685246
Bos primigenius (Aurochs)	0.08253071	0.0657585	0.06828854	0.05900014	0.05885495	0.06465915
Brazilian	0.0583106	0.04772667	0.06150761	0.04455889	0.0488027	0.05770961
Montafon	0.06868407	0.02944362	0.03297571	0.03670145	0.02150598	0.03243361
Buša	0.06950149	0.0313126	0.03865895	0.03556148	0.03161205	0.02840205

Chianina	0.08238254	0.04346927	0.04865581	0.05100791	0.04241488	0.04310193
Chillingham	0.06553	0.06273233	0.07166504	0.05483268	0.06688423	0.06525063
Devon	0.06157651	0.02375714	0.0427413	0.03710902	0.03399686	0.04282682
Egerlaender	0.08591209	0.04659032	0.03904281	0.05720841	0.03742785	0.04913054
Hérens	0.0546287	0.04571217	0.06522596	0.03957151	0.05092996	0.06262523
Fjällko	0.07486053	0.0264277	0.02853915	0.0419151	0.02689155	0.03041254
Bern Red Pied	0.06927664	0.03145225	0.03187625	0.03983993	0.02153411	0.03793114
Freiburg Red Pied	0.07701759	0.04886808	0.0560535	0.05441776	0.04350122	0.06102627
Simmental	0.05321712	0.03683291	0.05668588	0.03460842	0.03652585	0.05329366
South Moravian Red Pied	0.05949189	0.03619287	0.04366044	0.03920079	0.02685293	0.04208847
Rubia Gallega	0.09833576	0.06037489	0.05655139	0.06499036	0.05386946	0.05114621
Allgaeuer Grey	0.05451562	0.04608885	0.05635297	0.04537392	0.04447369	0.0583835
Krainer Grey	0.0688533	0.04335763	0.05454793	0.04782017	0.04209026	0.05634692
Oberinntaler Grey	0.06006574	0.03232149	0.04282839	0.03474168	0.0264805	0.04431474
Haná-Berne	0.07859308	0.04173428	0.04010774	0.04926493	0.02921202	0.03999138
Iceland	0.06290673	0.03964265	0.05683018	0.04276307	0.04453882	0.05192137
Jersey	0.06866104	0.05298141	0.07187753	0.05929726	0.06260418	0.07317966
Jersey (polled)	0.06130313	0.04964024	0.07083687	0.05439034	0.05896749	0.07212309
Jutland	0.08360647	0.04010082	0.03527052	0.05109173	0.03769888	0.03359346
Kampeten: Styrian Bergscheck	0.05325459	0.03135541	0.04287763	0.03329575	0.02735386	0.0418578
Kampeten: Welser Schecken	0.07432893	0.05403745	0.05549252	0.05286352	0.04399155	0.05397461
Kerry	0.0672097	0.02873205	0.03812506	0.03254057	0.02967113	0.03337658
Cretan	0.078743	0.0460416	0.05743507	0.05194902	0.05329684	0.0435946
Kuhlaender	0.06766193	0.02515488	0.03364597	0.0367334	0.01826221	0.02947076
Landschlag von Warnsdorf	0.06792455	0.04308049	0.05611851	0.04875098	0.04309572	0.05158207
Landschlag von Winkelsdorf	0.08946096	0.05257455	0.04272137	0.06074806	0.04144209	0.03973129
Landschlag X Simmental	0.08110903	0.04359965	0.03915675	0.04930962	0.03477905	0.03507863
Limousin	0.06572756	0.03228804	0.05210983	0.04555041	0.04018409	0.05488151
Moravian Landschlag	0.06036764	0.03249807	0.05020875	0.040199	0.0315165	0.04412096
Normande	0.06828162	0.0377044	0.05158834	0.05239516	0.04812367	0.06198353
North Wales	0.06701808	0.02429076	0.03689056	0.03470004	0.02765865	0.03294871
White Park	0.07532988	0.04358141	0.05794972	0.0496329	0.05238122	0.05026187
Pinzgauer	0.05924695	0.02807652	0.04602298	0.03545422	0.02776795	0.04664623

Pinzgauer X Scottish Highland	0.0710677	0.05846094	0.06061487	0.06135539	0.05520379	0.07086601
Pinzgauer X Simmentaler	0.06957473	0.0511397	0.07276434	0.05195526	0.05769684	0.07341386
Pinzgauer: Moelltaler	0.06782858	0.03359132	0.03952159	0.04057061	0.02783787	0.0350655
Pinzgauer: Pustertaler	0.06576335	0.07207902	0.08581565	0.07077925	0.07491304	0.08549116
Pirenaica	0.05594206	0.03012762	0.04508088	0.04052535	0.03565174	0.0460796
Red Poll		0.06585008	0.08117582	0.05422473	0.06813852	0.07834088
Maas-Rhein-Ijsselschlag	0.06585008		0.03267304	0.03262808	0.02486933	0.03481744
Danish Red	0.08117582	0.03267304		0.05006631	0.03041691	0.03417977
Angeln	0.05422473	0.03262808	0.05006631		0.03411712	0.04482402
Bohemian Red	0.06813852	0.02486933	0.03041691	0.03411712		0.02969264
Polish Red	0.07834088	0.03481744	0.03417977	0.04482402	0.02969264	
Sanga	0.10378891	0.06645617	0.05315203	0.07607072	0.06364921	0.05142357
Sardinian	0.08807132	0.04884217	0.04074724	0.06424479	0.04693873	0.04981217
Scheinfelder	0.0766889	0.04154465	0.04796217	0.04450684	0.03776973	0.04262515
Schoenhengster	0.07179437	0.0307507	0.03489728	0.03710918	0.01549548	0.03223348
Holland Black Pied	0.04627316	0.03949742	0.054256	0.0364614	0.04122772	0.05382809
Andalusian Black	0.07085684	0.04028724	0.04296348	0.04159562	0.03347094	0.03876446
Sweden (Jaemtland or Vatterbotten)	0.09717826	0.05764575	0.05164271	0.06915321	0.0575096	0.06331911
Scottish Highland	0.04271026	0.0518309	0.0708524	0.04215887	0.05468536	0.06964007
Sicilian	0.07164249	0.06158129	0.07056371	0.06128009	0.05709961	0.0737763
Småland	0.08836003	0.04063141	0.04071464	0.05049878	0.04122597	0.03394405
South Devon	0.07704287	0.02677842	0.03063663	0.04311631	0.02941113	0.03104462
Spanish Fighting Cattle	0.04173264	0.05853413	0.07191645	0.04543793	0.05739338	0.06931109
Buchara Grey	0.14082368	0.10877181	0.09690636	0.11338081	0.10485396	0.08701192
Hungarian Grey	0.05998785	0.03492534	0.04965389	0.03958434	0.03677065	0.050978
Hungarian Grey X Bern Red Pied	0.06611462	0.06373247	0.08107931	0.06128214	0.06553126	0.07819605
Sudeten	0.07084329	0.03210953	0.04198224	0.04372084	0.02931785	0.04097408
Sudeten X Simmental	0.07044195	0.04267863	0.05287971	0.05187559	0.04669041	0.0587981
Sudeten X Tesstal?	0.06341098	0.04193889	0.05321807	0.04682792	0.04060158	0.05224043
Tarentaise	0.05524464	0.04315457	0.0552441	0.04022587	0.04249329	0.05396237
Telemark	0.07071238	0.0253282	0.03554883	0.035462	0.02978395	0.03569051
Tudanca	0.07117147	0.04015871	0.04447101	0.04729582	0.03827887	0.03776734
Tuxer	0.05264051	0.08298691	0.10253863	0.0744871	0.08774543	0.10200742

Heck	0.06085537	0.03300248	0.04821903	0.0357609	0.03640725	0.04411254
Niata	0.1789749	0.20775596	0.23030579	0.20188278	0.21521437	0.23009036
Veredelter Landschlag	0.08033455	0.04310245	0.05038313	0.05167729	0.03787231	0.03890568
Vogtlaender	0.06074761	0.03800407	0.05615615	0.04612214	0.04070603	0.06032597
Waldviertler X Scheinfelder	0.06475632	0.03295134	0.04254307	0.04533556	0.03752901	0.04666443
Watussi	0.08449106	0.04676555	0.04514056	0.05797922	0.04096201	0.04916075
Zebu	0.09649187	0.05214669	0.04447968	0.06322968	0.04968191	0.03340395
Zebu (Africa)	0.09494296	0.05848057	0.0625064	0.06104988	0.05275484	0.05276478
Zebu (Asian)	0.08540272	0.04740082	0.05909346	0.06161924	0.05423916	0.05530938
Zebu (Indian)	0.09180316	0.05370648	0.04839428	0.06186807	0.04962465	0.03935037
Zillertaler	0.05367539	0.05094394	0.07222405	0.04985848	0.0571053	0.07236065

	Sanga	Sardinian	Scheinfelder	Schoenhengster	Holland Black Pied	Andalusian Black
Ayrshire	0.08324567	0.05683412	0.0593029	0.04652963	0.0461939	0.05273037
Blondvieh: Carinthian	0.06127921	0.04198042	0.04289409	0.02176712	0.0431021	0.03506691
Blondvieh: Lavanttaler	0.05996404	0.04062088	0.04253323	0.02539212	0.04490939	0.03398604
Blondvieh: Mariahofer	0.08007684	0.05987378	0.05644176	0.04683583	0.04122318	0.05342524
Blondvieh: Murbodner	0.06783411	0.04656537	0.04302931	0.0247152	0.04537137	0.03879405
Blondvieh: Waldviertler	0.05930493	0.04603027	0.04235654	0.02290094	0.04267554	0.03318252
Bos primigenius (Aurochs)	0.07393976	0.07018777	0.06114722	0.0572913	0.06149029	0.04381656
Brazilian	0.07849673	0.06070054	0.0501272	0.04945387	0.04423005	0.04367314
Montafon	0.05793314	0.04273589	0.04005134	0.01981632	0.04262663	0.02764374
Buša	0.05785965	0.055145	0.04250847	0.03535129	0.04932859	0.03700149
Chianina	0.06206184	0.05101828	0.0512264	0.04218385	0.05683121	0.04412854
Chillingham	0.0859188	0.08131681	0.07342043	0.06673126	0.06056219	0.0643616
Devon	0.07197535	0.05061024	0.04570541	0.03861066	0.04356257	0.04493857
Egerlaender	0.06186087	0.0489214	0.05069248	0.03779567	0.06138847	0.04567743
Hérens	0.09340492	0.07637989	0.06395935	0.05331883	0.04596957	0.06102196
Fjällko	0.06070583	0.05067935	0.04706131	0.03246504	0.04915268	0.04113023
Bern Red Pied	0.06242533	0.04387853	0.03971706	0.02141882	0.04368586	0.03178579
Freiburg Red Pied	0.08091916	0.06165589	0.05568409	0.04357104	0.05152196	0.05015919
Simmental	0.08394779	0.06162577	0.04787274	0.0370482	0.03361768	0.04316695
South Moravian Red Pied	0.07181681	0.05231864	0.04778732	0.02757921	0.03413945	0.03817729

Rubia Gallega	0.05810271	0.0594432	0.04974598	0.05173099	0.07400418	0.04668788
Allgaeuer Grey	0.08215926	0.06620184	0.05824766	0.04586641	0.04050474	0.05164627
Krainer Grey	0.08057567	0.05759406	0.05630132	0.0423131	0.04656117	0.05120021
Oberinntaler Grey	0.07320288	0.05208276	0.04741084	0.02900026	0.03799367	0.03903151
Haná-Berne	0.0629596	0.05114948	0.0444436	0.02795632	0.04812529	0.03461249
Iceland	0.08486337	0.06645599	0.05756424	0.04796075	0.04846206	0.05590338
Jersey	0.10582867	0.0820912	0.07859519	0.06988825	0.06424892	0.08187606
Jersey (polled)	0.10119549	0.07821579	0.07058914	0.06464261	0.05785026	0.07453949
Jutland	0.05599992	0.0485175	0.05170265	0.03806388	0.05718328	0.03996591
Kampeten: Styrian Bergscheck	0.07031523	0.05115351	0.04630128	0.02961409	0.03364204	0.03481305
Kampeten: Welser Schecken	0.07463999	0.06193428	0.0536904	0.04158083	0.04679773	0.03956974
Kerry	0.06626136	0.05367393	0.04391415	0.03275056	0.04387589	0.0347805
Cretan	0.06704099	0.06511637	0.05322662	0.05667081	0.06156205	0.05608263
Kuhlaender	0.06398647	0.04439664	0.0401564	0.02014905	0.04152582	0.03663776
Landschlag von Warnsdorf	0.08292995	0.06858212	0.05575566	0.04845552	0.05607511	0.06106568
Landschlag von Winkelsdorf	0.05440583	0.05052835	0.05505139	0.042032	0.06320691	0.0463464
Landschlag X Simmental	0.06036257	0.04683201	0.04659184	0.03531604	0.06094948	0.04255433
Limousin	0.08657005	0.05810537	0.05367366	0.04458719	0.04692862	0.05459045
Moravian Landschlag	0.08050437	0.06075221	0.0500287	0.03487123	0.03881497	0.04864471
Normande	0.08807763	0.05598211	0.0643773	0.05301329	0.05138478	0.06250334
North Wales	0.06390713	0.04697042	0.0380524	0.0291787	0.0429289	0.03487307
White Park	0.07486165	0.07240147	0.05421514	0.05598689	0.05973898	0.05887317
Pinzgauer	0.07833644	0.05241603	0.04763194	0.03092533	0.03854044	0.04544181
Pinzgauer X Scottish Highland	0.08607199	0.06674897	0.07104821	0.05619463	0.05939902	0.06231355
Pinzgauer X Simmentaler	0.10524062	0.07926907	0.06855785	0.06055135	0.05950668	0.07303943
Pinzgauer: Moelltaler	0.06271317	0.04657539	0.04035863	0.02653097	0.04264106	0.03430945
Pinzgauer: Pustertaler	0.10467708	0.08485139	0.08362055	0.07464911	0.06497973	0.07608
Pirenaica	0.07640032	0.05188059	0.05287014	0.0404013	0.0379182	0.04639594
Red Poll	0.10378891	0.08807132	0.0766889	0.07179437	0.04627316	0.07085684
Maas-Rhein-Ijsselschlag	0.06645617	0.04884217	0.04154465	0.0307507	0.03949742	0.04028724
Danish Red	0.05315203	0.04074724	0.04796217	0.03489728	0.054256	0.04296348
Angeln	0.07607072	0.06424479	0.04450684	0.03710918	0.0364614	0.04159562
Bohemian Red	0.06364921	0.04693873	0.03776973	0.01549548	0.04122772	0.03347094

Polish Red	0.05142357	0.04981217	0.04262515	0.03223348	0.05382809	0.03876446
Sanga		0.05537724	0.06029101	0.06366644	0.08207079	0.05566383
Sardinian	0.05537724		0.05300672	0.04798146	0.06755692	0.04842382
Scheinfelder	0.06029101	0.05300672		0.03941488	0.05570224	0.0341475
Schoenhengster	0.06366644	0.04798146	0.03941488		0.04326784	0.03173196
Holland Black Pied	0.08207079	0.06755692	0.05570224	0.04326784		0.04566839
Andalusian Black	0.05566383	0.04842382	0.0341475	0.03173196	0.04566839	
Sweden (Jaemtland or Vatterbotten)	0.07946201	0.07151472	0.07679341	0.06267348	0.07521207	0.07168379
Scottish Highland	0.09883563	0.07829803	0.06746109	0.05578151	0.04157824	0.06166932
Sicilian	0.09643994	0.07205903	0.07428724	0.05878601	0.06153994	0.06810285
Småland	0.06023298	0.0575928	0.04779918	0.04276425	0.06210927	0.04507223
South Devon	0.05650667	0.04388193	0.04260037	0.03183549	0.05089228	0.03970393
Spanish Fighting Cattle	0.09382456	0.07613557	0.0648186	0.05834408	0.04092275	0.05231464
Buchara Grey	0.07601348	0.10008878	0.09697809	0.10185908	0.11824343	0.09437283
Hungarian Grey	0.07809096	0.05412093	0.04801129	0.03895597	0.04053493	0.04576103
Hungarian Grey X Bern Red Pied	0.10457349	0.08340902	0.07369663	0.06855878	0.05266981	0.06849729
Sudeten	0.07072106	0.05797113	0.05447329	0.03410107	0.04523631	0.04716319
Sudeten X Simmental	0.08850645	0.06590113	0.06553059	0.05350499	0.05455263	0.06685897
Sudeten X Tesstal?	0.08195257	0.06316869	0.05494192	0.04272638	0.04287687	0.05191797
Tarentaise	0.08236021	0.06202822	0.05763385	0.04544187	0.04268417	0.04860682
Telemark	0.06717946	0.0521178	0.04561522	0.03236096	0.04403615	0.04113671
Tudanca	0.0604634	0.05282596	0.05121423	0.03782187	0.04723012	0.03930362
Tuxer	0.12724822	0.10464387	0.09706321	0.08850945	0.06733427	0.09326599
Heck	0.07396168	0.05773137	0.04775402	0.04056116	0.04175913	0.04351156
Niata	0.25790662	0.23070526	0.22249147	0.21814479	0.19849536	0.2274588
Veredelter Landschlag	0.06944694	0.05651618	0.04812267	0.04070212	0.05678975	0.04810589
Vogtlaender	0.09129852	0.06237641	0.06104304	0.04486137	0.04460366	0.05876631
Waldviertler X Scheinfelder	0.07215004	0.05315468	0.04691077	0.04087818	0.04481606	0.04612413
Watussi	0.06458756	0.04904321	0.0484355	0.04066798	0.06100109	0.04494645
Zebu	0.038611	0.05194901	0.05045257	0.04996459	0.07157913	0.0481152
Zebu (Africa)	0.07410639	0.06777168	0.05012848	0.05303795	0.07131717	0.0525349
Zebu (Asian)	0.08024642	0.05878771	0.06114959	0.05719426	0.06314435	0.06312054
Zebu (Indian)	0.04905263	0.04981456	0.0513121	0.04815304	0.06626308	0.04800194

Zillertaler	0.10213818	0.07639703	0.0721494	0.05827318	0.04469871	0.06695781
	Sweden (Jaemtland or Vatterbotten)	Scottish Highland	Sicilian	Småland	South Devon	Spanish Fighting Cattle
Ayrshire	0.0580801	0.05001926	0.06159061	0.05316119	0.0462412	0.05389274
Blondvieh: Carinthian	0.05929315	0.05727377	0.05756516	0.04405478	0.02563752	0.05919467
Blondvieh: Lavanttaler	0.06003434	0.05982746	0.05803314	0.04771227	0.03507044	0.05673718
Blondvieh: Mariahofer	0.07492729	0.0500434	0.06168711	0.06632894	0.05195239	0.05078145
Blondvieh: Murbodner	0.06463642	0.05034591	0.05196366	0.05031339	0.03135909	0.05803057
Blondvieh: Waldviertler	0.05953403	0.05621169	0.06399953	0.03641398	0.02856157	0.0604356
Bos primigenius (Aurochs)	0.08591642	0.07457872	0.06893892	0.06846919	0.06533874	0.06594447
Brazilian	0.09054907	0.0460348	0.05825798	0.0672605	0.04965205	0.0502273
Montafon	0.06362874	0.05436627	0.05908531	0.04410009	0.02834701	0.05532777
Buša	0.05901932	0.05957775	0.07267269	0.03618026	0.03818905	0.05926604
Chianina	0.07986387	0.06816892	0.07213992	0.05375634	0.03601091	0.07538017
Chillingham	0.08617472	0.06349475	0.09147061	0.06327861	0.07111721	0.05638618
Devon	0.06565591	0.04829763	0.06061632	0.05155519	0.03054815	0.05562106
Egerlaender	0.06546357	0.07476643	0.06446396	0.06053085	0.04513003	0.07535483
Hérens	0.07222729	0.04170849	0.06666363	0.06411113	0.06232179	0.04479005
Fjällko	0.04475646	0.06387333	0.06666923	0.03331505	0.02969815	0.06501515
Bern Red Pied	0.06171359	0.05730306	0.05768972	0.04804739	0.03065773	0.05753268
Freiburg Red Pied	0.08242201	0.06035773	0.06361149	0.06681601	0.0478108	0.06815371
Simmental	0.07955806	0.04031538	0.05693563	0.06047726	0.04712543	0.04376624
South Moravian Red Pied	0.06992602	0.04895656	0.05239607	0.0562756	0.04078595	0.04984061
Rubia Gallega	0.08950768	0.08087421	0.08687662	0.05711006	0.04716568	0.08553282
Allgaeuer Grey	0.07889928	0.05045008	0.05506429	0.06714519	0.05457536	0.05260696
Krainer Grey	0.08031665	0.04589841	0.06211009	0.06199549	0.04773135	0.06162969
Oberinntaler Grey	0.05900831	0.04790721	0.0557175	0.04841289	0.04283922	0.04539337
Haná-Berne	0.06898383	0.06439453	0.06284305	0.0473726	0.03883264	0.06676728
Iceland	0.06475345	0.05233039	0.07181814	0.04807235	0.05164277	0.05591001
Jersey	0.07884687	0.06021796	0.07180891	0.07733335	0.06699462	0.0709312
Jersey (polled)	0.08268069	0.05143257	0.07607262	0.07566845	0.06527367	0.06358493
Jutland	0.05985098	0.07113895	0.0736051	0.03770982	0.03726812	0.07119718
Kampeten: Styrian Bergscheck	0.06175126	0.04563605	0.05323996	0.05167689	0.04271661	0.04021988

Kampeten: Welser Schecken	0.08177579	0.06603566	0.07059644	0.06217619	0.05573941	0.0601383
Kerry	0.05788031	0.05439381	0.0699507	0.03206047	0.03315721	0.0532241
Cretan	0.08584366	0.07111706	0.08609028	0.05506278	0.0489089	0.07616286
Kuhlaender	0.06312248	0.05371469	0.057332	0.04295178	0.029045	0.05907887
Landschlag von Warnsdorf	0.0737639	0.05666859	0.06360661	0.06365999	0.04886902	0.06884454
Landschlag von Winkelsdorf	0.07297514	0.08221077	0.07493262	0.05470054	0.04484036	0.08032679
Landschlag X Simmental	0.06896254	0.07063856	0.06977608	0.04986992	0.03966414	0.06956781
Limousin	0.0741499	0.04920176	0.05995568	0.06170473	0.0438779	0.05885674
Moravian Landschlag	0.07173518	0.04742141	0.05885101	0.05533895	0.04193588	0.05501067
Normande	0.0700175	0.05656646	0.0615586	0.06917016	0.04988292	0.06145599
North Wales	0.07023695	0.04911508	0.06511929	0.04083429	0.02716794	0.0572226
White Park	0.06838392	0.06827201	0.08432552	0.04918777	0.05425143	0.07014671
Pinzgauer	0.07047662	0.04041485	0.05334207	0.05506338	0.03606375	0.05221629
Pinzgauer X Scottish Highland	0.07334647	0.06221807	0.05758767	0.08044594	0.06756454	0.06231095
Pinzgauer X Simmentaler	0.08611379	0.0483736	0.06482116	0.07660906	0.06469213	0.06544877
Pinzgauer: Moelltaler	0.07524387	0.05470747	0.0615476	0.05205178	0.02950064	0.06177479
Pinzgauer: Pustertaler	0.10390869	0.06547776	0.07830055	0.09363195	0.08485617	0.06233908
Pirenaica	0.07164333	0.04338389	0.06360318	0.05321025	0.03971189	0.05040342
Red Poll	0.09717826	0.04271026	0.07164249	0.08836003	0.07704287	0.04173264
Maas-Rhein-Ijsselschlag	0.05764575	0.0518309	0.06158129	0.04063141	0.02677842	0.05853413
Danish Red	0.05164271	0.0708524	0.07056371	0.04071464	0.03063663	0.07191645
Angeln	0.06915321	0.04215887	0.06128009	0.05049878	0.04311631	0.04543793
Bohemian Red	0.0575096	0.05468536	0.05709961	0.04122597	0.02941113	0.05739338
Polish Red	0.06331911	0.06964007	0.0737763	0.03394405	0.03104462	0.06931109
Sanga	0.07946201	0.09883563	0.09643994	0.06023298	0.05650667	0.09382456
Sardinian	0.07151472	0.07829803	0.07205903	0.0575928	0.04388193	0.07613557
Scheinfelder	0.07679341	0.06746109	0.07428724	0.04779918	0.04260037	0.0648186
Schoenhengster	0.06267348	0.05578151	0.05878601	0.04276425	0.03183549	0.05834408
Holland Black Pied	0.07521207	0.04157824	0.06153994	0.06210927	0.05089228	0.04092275
Andalusian Black	0.07168379	0.06166932	0.06810285	0.04507223	0.03970393	0.05231464
Sweden (Jaemtland or Vatterbotten)		0.08944157	0.07781926	0.05997852	0.06353016	0.08494366
Scottish Highland	0.08944157		0.05937057	0.07609938	0.06259757	0.04383706
Sicilian	0.07781926	0.05937057		0.08514217	0.06484373	0.06615259

Småland	0.05997852	0.07609938	0.08514217		0.04085278	0.07433398
South Devon	0.06353016	0.06259757	0.06484373	0.04085278		0.06938915
Spanish Fighting Cattle	0.08494366	0.04383706	0.06615259	0.07433398	0.06938915	
Buchara Grey	0.12835333	0.13370404	0.13893317	0.0965673	0.09552283	0.13306514
Hungarian Grey	0.07671419	0.04469558	0.0566197	0.05931283	0.03919561	0.05253812
Hungarian Grey X Bern Red Pied	0.10316817	0.05602907	0.07182988	0.08721445	0.06898852	0.0648603
Sudeten	0.06175899	0.0576994	0.05605841	0.04879282	0.03619763	0.06432984
Sudeten X Simmental	0.06084579	0.06207287	0.06869447	0.06345581	0.05690148	0.06302952
Sudeten X Tesstal?	0.07597073	0.05086992	0.05677165	0.06410327	0.04503332	0.06018308
Tarentaise	0.06977885	0.05264825	0.0650423	0.05914711	0.05800828	0.04139127
Telemark	0.05947657	0.05290732	0.06611689	0.03692005	0.03248186	0.06143869
Tudanca	0.07239388	0.06014206	0.06365371	0.05306506	0.03768685	0.0651769
Tuxer	0.11587862	0.05501792	0.08013442	0.11169377	0.09774684	0.06210731
Heck	0.07207154	0.04200586	0.06588716	0.04727565	0.03845731	0.0537238
Niata	0.2343151	0.17787335	0.19712786	0.23541219	0.22247606	0.19166878
Veredelter Landschlag	0.07048696	0.06974461	0.07266254	0.04773652	0.04637892	0.06725167
Vogtlaender	0.0717683	0.04190533	0.05088156	0.0669653	0.05136413	0.05301209
Waldviertler X Scheinfelder	0.06445527	0.05827489	0.06711512	0.05307461	0.04510115	0.05486278
Watussi	0.05993102	0.07451502	0.06100253	0.05578396	0.04282319	0.07022655
Zebu	0.06745728	0.08911941	0.09139259	0.04343429	0.04349876	0.08440074
Zebu (Africa)	0.07697242	0.08207946	0.08811619	0.0493143	0.05741617	0.07707167
Zebu (Asian)	0.08101122	0.07412975	0.07686251	0.06359087	0.04969324	0.07766413
Zebu (Indian)	0.07918716	0.08363677	0.08627507	0.05501351	0.04459459	0.08136256
Zillertaler	0.08993977	0.03587191	0.06269382	0.0796255	0.06736383	0.05109725

	Bucharan Grey	Hungarian Grey	Hungarian Grey X Bern Red Pied	Sudeten	Sudeten X Simmental	Sudeten X Tesstal?
Ayrshire	0.12661497	0.04384131	0.06685376	0.04378285	0.04213798	0.05073924
Blondvieh: Carinthian	0.10285956	0.03594663	0.06635114	0.03059601	0.04925052	0.03991095
Blondvieh: Lavanttaler	0.10395813	0.04191359	0.07190107	0.03677814	0.04711952	0.04602137
Blondvieh: Mariahofer	0.12211763	0.04222561	0.06552472	0.04720786	0.04638151	0.04812805
Blondvieh: Murbodner	0.10763348	0.03147786	0.06270357	0.03649404	0.05188329	0.04041977
Blondvieh: Waldviertler	0.09775977	0.04307875	0.07006501	0.03076469	0.05195697	0.04656831

Bos primigenius (Aurochs)	0.10571126	0.06721091	0.07993064	0.0645172	0.08808579	0.07119904
Brazilian	0.11200608	0.03326778	0.04775365	0.05223672	0.06891977	0.0480865
Montafon	0.09798845	0.03707033	0.06740633	0.03702177	0.05565977	0.04358297
Buša	0.09868669	0.05135918	0.07731051	0.04356036	0.05537594	0.05461022
Chianina	0.09151505	0.05068916	0.07273043	0.04754669	0.07089079	0.0554933
Chillingham	0.11748491	0.06914704	0.08878886	0.06904169	0.06821072	0.07778613
Devon	0.11345115	0.03100524	0.05893705	0.04003462	0.04496237	0.03981275
Egerlaender	0.10569843	0.05425324	0.07841225	0.04696937	0.06356471	0.05631878
Hérens	0.13509403	0.04912121	0.06839362	0.05421751	0.04340722	0.0576939
Fjällko	0.10461014	0.04845509	0.07641016	0.0351097	0.0480045	0.04833455
Bern Red Pied	0.10286567	0.03571665	0.06734605	0.03701598	0.05271679	0.04327043
Freiburg Red Pied	0.11207304	0.04227381	0.06233222	0.04596383	0.06660909	0.0497793
Simmental	0.12141696	0.02980927	0.04793391	0.04238462	0.05140951	0.0415195
South Moravian Red Pied	0.11001367	0.03457542	0.05938711	0.03600175	0.0506728	0.04012172
Rubia Gallega	0.07781375	0.0630602	0.08569337	0.06646598	0.08981568	0.07034706
Allgaeuer Grey	0.11899932	0.04539009	0.06141236	0.04460194	0.05957512	0.05207571
Krainer Grey	0.11684854	0.042535	0.06438066	0.04616449	0.05962813	0.04820728
Oberinntaler Grey	0.11694197	0.03522193	0.06581193	0.03847859	0.04547412	0.04563898
Haná-Berne	0.10071729	0.04682392	0.06865983	0.03908216	0.06491073	0.04798867
Iceland	0.12554845	0.04921226	0.07174674	0.04886451	0.04908897	0.0529987
Jersey	0.14876288	0.05931665	0.07540957	0.05904119	0.04290894	0.06248188
Jersey (polled)	0.14365432	0.05410085	0.07411331	0.06497399	0.05211355	0.06275583
Jutland	0.09107113	0.05560958	0.08184993	0.04498254	0.06633238	0.05664633
Kampeten: Styrian Bergscheck	0.11452085	0.03814438	0.06463858	0.03769155	0.04598858	0.04493601
Kampeten: Welser Schecken	0.09987045	0.0545016	0.07035255	0.05660971	0.07636512	0.0588225
Kerry	0.104018	0.04400028	0.07040254	0.04159442	0.05272112	0.04784745
Cretan	0.09399417	0.05803786	0.07716042	0.05817963	0.06953812	0.06459204
Kuhlaender	0.10202659	0.03473993	0.06316913	0.03012961	0.0502899	0.04158298
Landschlag von Warnsdorf	0.12462814	0.04759919	0.06659815	0.0449372	0.05183536	0.04577238
Landschlag von Winkelsdorf	0.09094893	0.06066596	0.08366749	0.04881196	0.0719206	0.06441986
Landschlag X Simmental	0.09779348	0.05009317	0.08134647	0.04850385	0.06138916	0.05654279
Limousin	0.12407691	0.02876841	0.05180322	0.04528251	0.04693223	0.04176672
Moravian Landschlag	0.11539933	0.03653543	0.05759004	0.03632585	0.05161578	0.03934717

Normande	0.1306243	0.04213248	0.06721642	0.05293464	0.0397019	0.05215655
North Wales	0.09911153	0.02980069	0.06096665	0.03694305	0.0515475	0.04222244
White Park	0.1156078	0.0642237	0.08331393	0.05540987	0.05722837	0.06266122
Pinzgauer	0.11751627	0.02550828	0.05625353	0.03580536	0.04473946	0.03760322
Pinzgauer X Scottish Highland	0.12934227	0.05944639	0.08347551	0.06210365	0.06415043	0.0667908
Pinzgauer X Simmentaler	0.14131583	0.04863163	0.06537279	0.05771147	0.05510306	0.06088003
Pinzgauer: Moelltaler	0.0932288	0.03294734	0.05997098	0.03987367	0.0600082	0.04027869
Pinzgauer: Pustertaler	0.14487604	0.06904434	0.08216507	0.07831474	0.07743803	0.08315607
Pirenaica	0.11492516	0.03422522	0.05770602	0.03945733	0.04455174	0.04198729
Red Poll	0.14082368	0.05998785	0.06611462	0.07084329	0.07044195	0.06341098
Maas-Rhein-Ijsselschlag	0.10877181	0.03492534	0.06373247	0.03210953	0.04267863	0.04193889
Danish Red	0.09690636	0.04965389	0.08107931	0.04198224	0.05287971	0.05321807
Angeln	0.11338081	0.03958434	0.06128214	0.04372084	0.05187559	0.04682792
Bohemian Red	0.10485396	0.03677065	0.06553126	0.02931785	0.04669041	0.04060158
Polish Red	0.08701192	0.050978	0.07819605	0.04097408	0.0587981	0.05224043
Sanga	0.07601348	0.07809096	0.10457349	0.07072106	0.08850645	0.08195257
Sardinian	0.10008878	0.05412093	0.08340902	0.05797113	0.06590113	0.06316869
Scheinfelder	0.09697809	0.04801129	0.07369663	0.05447329	0.06553059	0.05494192
Schoenhengster	0.10185908	0.03895597	0.06855878	0.03410107	0.05350499	0.04272638
Holland Black Pied	0.11824343	0.04053493	0.05266981	0.04523631	0.05455263	0.04287687
Andalusian Black	0.09437283	0.04576103	0.06849729	0.04716319	0.06685897	0.05191797
Sweden (Jaemtland or Vatterbotten)	0.12835333	0.07671419	0.10316817	0.06175899	0.06084579	0.07597073
Scottish Highland	0.13370404	0.04469558	0.05602907	0.0576994	0.06207287	0.05086992
Sicilian	0.13893317	0.0566197	0.07182988	0.05605841	0.06869447	0.05677165
Småland	0.0965673	0.05931283	0.08721445	0.04879282	0.06345581	0.06410327
South Devon	0.09552283	0.03919561	0.06898852	0.03619763	0.05690148	0.04503332
Spanish Fighting Cattle	0.13306514	0.05253812	0.0648603	0.06432984	0.06302952	0.06018308
Buchara Grey		0.11499455	0.13396602	0.1131285	0.13401074	0.11725112
Hungarian Grey	0.11499455		0.04772682	0.04420683	0.0529975	0.04215061
Hungarian Grey X Bern Red Pied	0.13396602	0.04772682		0.06479276	0.07808009	0.05872392
Sudeten	0.1131285	0.04420683	0.06479276		0.04958162	0.04470457
Sudeten X Simmental	0.13401074	0.0529975	0.07808009	0.04958162		0.05755677
Sudeten X Tesstal?	0.11725112	0.04215061	0.05872392	0.04470457	0.05755677	

Tarentaise	0.12480224	0.04991231	0.07065492	0.04975015	0.04767653	0.0578546
Telemark	0.10387214	0.0427834	0.07053002	0.03790442	0.05240273	0.04643517
Tudanca	0.09186041	0.04644528	0.07032943	0.04192727	0.06616527	0.04859225
Tuxer	0.16310817	0.07498555	0.07761504	0.08909286	0.08093821	0.08316408
Heck	0.11001878	0.03672067	0.0577288	0.04029309	0.05193491	0.04306894
Niata	0.2923451	0.19624373	0.18830224	0.21158942	0.19282882	0.2030306
Veredelter Landschlag	0.10626364	0.04704383	0.07250766	0.04613291	0.05821462	0.05630586
Vogtlaender	0.13596732	0.03575106	0.0563863	0.04124561	0.03921067	0.04372402
Waldviertler X Scheinfelder	0.11582919	0.04131901	0.06889046	0.04462415	0.0457746	0.05239115
Watussi	0.10838383	0.05339783	0.08047381	0.05250364	0.06493391	0.05468513
Zebu	0.07611923	0.0673259	0.09458806	0.06023784	0.07425368	0.07110492
Zebu (Africa)	0.10673878	0.06066757	0.08470676	0.06798433	0.07731591	0.0719207
Zebu (Asian)	0.1122263	0.05025934	0.06888739	0.05705628	0.0631518	0.0609244
Zebu (Indian)	0.07517239	0.06120434	0.08600224	0.06080405	0.07724613	0.06526532
Zillertaler	0.14029012	0.04907272	0.05971245	0.05848709	0.05546119	0.0575406
	Tarentaise	Telemark	Tudanca	Tuxer	Heck	Niata
Ayrshire	0.04310943	0.03585532	0.05448215	0.08074834	0.03680974	0.2001066
Blondvieh: Carinthian	0.0471061	0.03358829	0.03661116	0.08976983	0.03970676	0.21785852
Blondvieh: Lavanttaler	0.04114471	0.03727013	0.03985649	0.08790813	0.04370497	0.21672426
Blondvieh: Mariahofer	0.04139913	0.04960964	0.05182328	0.06704336	0.04848608	0.1917154
Blondvieh: Murbodner	0.04805287	0.03501106	0.04034021	0.0853241	0.03777958	0.2101028
Blondvieh: Waldviertler	0.04438519	0.02407105	0.0339334	0.09023329	0.03440533	0.2214515
Bos primigenius (Aurochs)	0.067714	0.05921042	0.05470843	0.10231258	0.06455687	0.23560062
Brazilian	0.05632764	0.05357093	0.04615491	0.07640454	0.04360212	0.19940255
Montafon	0.04614827	0.03365515	0.03275885	0.08796387	0.03921452	0.21836387
Buša	0.04476911	0.03138406	0.04207617	0.09162124	0.03889971	0.22076865
Chianina	0.06331203	0.03981271	0.03600972	0.09940741	0.04680111	0.22550462
Chillingham	0.05391231	0.05971192	0.06766769	0.08383216	0.05474822	0.20999175
Devon	0.04696332	0.03431855	0.04594533	0.07900308	0.0310871	0.20038637
Egerlaender	0.06058537	0.05377897	0.05208502	0.1015944	0.05887488	0.22620483
Hérens	0.04147074	0.04997393	0.0644971	0.06405662	0.04378607	0.18321264
Fjällko	0.05122657	0.02667697	0.04044464	0.09737494	0.04013857	0.22397731
Bern Red Pied	0.046335	0.0364832	0.03712596	0.08794526	0.04247428	0.21749059

Freiburg Red Pied	0.06086134	0.05119719	0.04928874	0.08910063	0.04813792	0.20962226
Simmental	0.04114081	0.04518199	0.04950914	0.06755247	0.03634379	0.19246641
South Moravian Red Pied	0.04461065	0.04143559	0.03713752	0.07792068	0.0418108	0.20687725
Rubia Gallega	0.08004731	0.05508099	0.05399622	0.11919144	0.06064637	0.24610543
Allgaeuer Grey	0.04939302	0.05322942	0.05044295	0.07059129	0.05159659	0.19928193
Krainer Grey	0.05530836	0.03915911	0.04806854	0.08209764	0.03946429	0.20303588
Oberinntaler Grey	0.03303365	0.033949	0.04486086	0.0787816	0.03671476	0.20515185
Haná-Berne	0.05614359	0.03833616	0.04191334	0.09942955	0.04590395	0.22771185
Iceland	0.04803316	0.03981323	0.0598226	0.08452961	0.0380076	0.20193559
Jersey	0.06009782	0.06171403	0.07695607	0.0769015	0.05561512	0.17428497
Jersey (polled)	0.05632126	0.05882304	0.07425541	0.06932441	0.05492696	0.17558512
Jutland	0.05838768	0.03366613	0.03721381	0.1062414	0.04715603	0.23651907
Kampeten: Styrian Bergscheck	0.0287234	0.03575799	0.04135579	0.07269224	0.03764615	0.20448827
Kampeten: Welser Schecken	0.05812826	0.04840666	0.04518263	0.09322267	0.0561721	0.22708997
Kerry	0.04496781	0.02436683	0.04309677	0.09162134	0.0321467	0.21899999
Cretan	0.06464466	0.05060085	0.05122573	0.09726794	0.04932887	0.21617914
Kuhlaender	0.04419816	0.02959791	0.03618714	0.08569177	0.03612828	0.21367789
Landschlag von Warnsdorf	0.05952195	0.05067832	0.05711422	0.08475512	0.04432479	0.19815463
Landschlag von Winkelsdorf	0.06796265	0.05366125	0.0471081	0.10906038	0.06315743	0.24138393
Landschlag X Simmental	0.05606982	0.0472139	0.04821708	0.10049797	0.05497694	0.22641364
Limousin	0.0510784	0.04661896	0.05558279	0.07589794	0.04100489	0.18976865
Moravian Landschlag	0.0464058	0.04136634	0.0486542	0.07538204	0.03973018	0.19943347
Normande	0.0507449	0.05156046	0.06103337	0.07659845	0.05157877	0.19025759
North Wales	0.04696258	0.02645853	0.03655916	0.08578937	0.02507081	0.21148885
White Park	0.05754494	0.04717776	0.0637195	0.09479388	0.04638604	0.21498226
Pinzgauer	0.04307757	0.03455284	0.04390969	0.07246003	0.0323122	0.19465177
Pinzgauer X Scottish Highland	0.0546689	0.06360775	0.06303912	0.07813122	0.06668611	0.20390199
Pinzgauer X Simmentaler	0.05564042	0.05636496	0.07170912	0.06809716	0.05100621	0.17358481
Pinzgauer: Moelltaler	0.05170263	0.03677348	0.03034898	0.08751029	0.03824009	0.21494517
Pinzgauer: Pustertaler	0.06077526	0.07718721	0.07583583	0.06420203	0.07542103	0.18533463
Pirenaica	0.04234276	0.03686007	0.04509081	0.07556486	0.0293085	0.19881641
Red Poll	0.05524464	0.07071238	0.07117147	0.05264051	0.06085537	0.1789749
Maas-Rhein-Ijsselschlag	0.04315457	0.0253282	0.04015871	0.08298691	0.03300248	0.20775596

Danish Red	0.0552441	0.03554883	0.04447101	0.10253863	0.04821903	0.23030579
Angeln	0.04022587	0.035462	0.04729582	0.0744871	0.0357609	0.20188278
Bohemian Red	0.04249329	0.02978395	0.03827887	0.08774543	0.03640725	0.21521437
Polish Red	0.05396237	0.03569051	0.03776734	0.10200742	0.04411254	0.23009036
Sanga	0.08236021	0.06717946	0.0604634	0.12724822	0.07396168	0.25790662
Sardinian	0.06202822	0.0521178	0.05282596	0.10464387	0.05773137	0.23070526
Scheinfelder	0.05763385	0.04561522	0.05121423	0.09706321	0.04775402	0.22249147
Schoenhengster	0.04544187	0.03236096	0.03782187	0.08850945	0.04056116	0.21814479
Holland Black Pied	0.04268417	0.04403615	0.04723012	0.06733427	0.04175913	0.19849536
Andalusian Black	0.04860682	0.04113671	0.03930362	0.09326599	0.04351156	0.2274588
Sweden (Jaemtland or Vatterbotten)	0.06977885	0.05947657	0.07239388	0.11587862	0.07207154	0.2343151
Scottish Highland	0.05264825	0.05290732	0.06014206	0.05501792	0.04200586	0.17787335
Sicilian	0.0650423	0.06611689	0.06365371	0.08013442	0.06588716	0.19712786
Småland	0.05914711	0.03692005	0.05306506	0.11169377	0.04727565	0.23541219
South Devon	0.05800828	0.03248186	0.03768685	0.09774684	0.03845731	0.22247606
Spanish Fighting Cattle	0.04139127	0.06143869	0.0651769	0.06210731	0.0537238	0.19166878
Buchara Grey	0.12480224	0.10387214	0.09186041	0.16310817	0.11001878	0.2923451
Hungarian Grey	0.04991231	0.0427834	0.04644528	0.07498555	0.03672067	0.19624373
Hungarian Grey X Bern Red Pied	0.07065492	0.07053002	0.07032943	0.07761504	0.0577288	0.18830224
Sudeten	0.04975015	0.03790442	0.04192727	0.08909286	0.04029309	0.21158942
Sudeten X Simmental	0.04767653	0.05240273	0.06616527	0.08093821	0.05193491	0.19282882
Sudeten X Tesstal?	0.0578546	0.04643517	0.04859225	0.08316408	0.04306894	0.2030306
Tarentaise		0.04793559	0.05705471	0.07034	0.04672141	0.19986042
Telemark	0.04793559		0.03727606	0.08977874	0.02991952	0.21663914
Tudanca	0.05705471	0.03727606		0.091291	0.04401142	0.22204995
Tuxer	0.07034	0.08977874	0.091291		0.08160581	0.15373326
Heck	0.04672141	0.02991952	0.04401142	0.08160581		0.20379952
Niata	0.19986042	0.21663914	0.22204995	0.15373326	0.20379952	
Veredelter Landschlag	0.05492997	0.04728155	0.05612909	0.09786598	0.04863234	0.21841139
Vogtlaender	0.04503589	0.04816155	0.05988072	0.0668155	0.04269203	0.18287529
Waldviertler X Scheinfelder	0.04177701	0.04375428	0.05238812	0.07998069	0.04454391	0.206752
Watussi	0.05777691	0.05137326	0.0549763	0.10174542	0.05690372	0.22955942
Zebu	0.07046544	0.05256389	0.05327252	0.11843484	0.06025385	0.24769697

Zebu (Africa)	0.0689476	0.05883728	0.07000977	0.11520804	0.06075216	0.2315688
Zebu (Asian)	0.06618855	0.05300663	0.06027731	0.0973639	0.05243446	0.20864069
Zebu (Indian)	0.06938103	0.05444816	0.04956812	0.11153585	0.06084161	0.23925479
Zillertaler	0.04733109	0.05666412	0.06314797	0.04998966	0.05196455	0.17110927

	Veredelter Landschlag	Vogtlaender	Waldviertler X Scheinfelder	Watussi	Zebu	Zebu (Africa)
Ayrshire	0.05387092	0.03652396	0.04424611	0.05480974	0.06806406	0.06710952
Blondvieh: Carinthian	0.04180249	0.04223469	0.03628689	0.03945152	0.05104088	0.05984224
Blondvieh: Lavanttaler	0.04548011	0.04475204	0.03634589	0.04357396	0.05145921	0.06155172
Blondvieh: Mariahofer	0.05881627	0.04034813	0.03856881	0.06131459	0.07225489	0.07812052
Blondvieh: Murbodner	0.04117698	0.03791814	0.04135505	0.04096565	0.05785764	0.05570806
Blondvieh: Waldviertler	0.04350537	0.04750086	0.03887055	0.04534438	0.04493392	0.0574628
Bos primigenius (Aurochs)	0.06960616	0.07853255	0.07533774	0.06657304	0.07369871	0.07473395
Brazilian	0.05904333	0.0507037	0.05357903	0.06224117	0.07309735	0.06988286
Montafon	0.04448275	0.04739855	0.04055802	0.04204826	0.04797925	0.05694083
Buša	0.04896987	0.05623123	0.04440324	0.05310046	0.04114813	0.05412012
Chianina	0.05919109	0.06386175	0.06124246	0.0594277	0.05261153	0.0686945
Chillingham	0.07194605	0.07317974	0.06309944	0.08259489	0.0762221	0.08520247
Devon	0.04756851	0.03513325	0.03531828	0.04825022	0.05654236	0.06046848
Egerlaender	0.05685267	0.0576769	0.05258861	0.04676637	0.05812739	0.06886728
Hérens	0.05986739	0.04030343	0.04700253	0.06947185	0.07847647	0.0719655
Fjällko	0.04717186	0.05108186	0.04079254	0.04563175	0.04459471	0.05727699
Bern Red Pied	0.04631252	0.04438415	0.0365365	0.03868477	0.05185039	0.05814418
Freiburg Red Pied	0.05994269	0.05026706	0.05549489	0.05728023	0.07334223	0.07491208
Simmental	0.04990193	0.03429012	0.04021584	0.05610289	0.07100937	0.06513375
South Moravian Red Pied	0.04480491	0.04090744	0.04234253	0.05100197	0.06186849	0.06485944
Rubia Gallega	0.0643776	0.08130445	0.0717653	0.06057663	0.05421053	0.0640188
Allgaeuer Grey	0.06014574	0.04758063	0.04445098	0.06209621	0.07587165	0.08113661
Krainer Grey	0.06066889	0.04406961	0.05765322	0.06255104	0.07223729	0.06915268
Oberinntaler Grey	0.04379805	0.03614105	0.03718349	0.04507432	0.06121353	0.05672112
Haná-Berne	0.04404392	0.05478804	0.05143832	0.04555523	0.05628593	0.05678896
Iceland	0.04936592	0.0458232	0.04807461	0.06095363	0.06746473	0.05942589

Jersey	0.07154265	0.04344124	0.06110581	0.08012647	0.09127122	0.08944247
Jersey (polled)	0.07008359	0.04471132	0.05867523	0.07529861	0.08722718	0.08148819
Jutland	0.05266474	0.06470462	0.05312331	0.05389136	0.04534552	0.0625783
Kampeten: Styrian Bergscheck	0.04614728	0.03728852	0.03407778	0.04680642	0.05905037	0.0608519
Kampeten: Welser Schecken	0.06044118	0.06670175	0.06166055	0.06185824	0.06609835	0.06853898
Kerry	0.04632448	0.05086289	0.043467	0.04989997	0.04795029	0.05119627
Cretan	0.06071678	0.06878152	0.05991302	0.0726784	0.05173739	0.06799722
Kuhlaender	0.03701564	0.0404372	0.03738192	0.04360306	0.05105565	0.05591948
Landschlag von Warnsdorf	0.05631096	0.04341149	0.04663095	0.06013102	0.07033085	0.07193207
Landschlag von Winkelsdorf	0.05225479	0.06962594	0.05739888	0.05675341	0.05101402	0.06971519
Landschlag X Simmental	0.04776374	0.0583015	0.05033356	0.05047159	0.05208427	0.05983607
Limousin	0.04942809	0.02863936	0.04059151	0.05644954	0.07223318	0.06498749
Moravian Landschlag	0.04285264	0.03638792	0.04218459	0.05259734	0.06470773	0.06227668
Normande	0.06242511	0.03534051	0.04648631	0.06172016	0.07575571	0.07881741
North Wales	0.04132526	0.04283257	0.03607662	0.04967788	0.05084178	0.05582075
White Park	0.05913685	0.06336112	0.04954937	0.06605886	0.05759544	0.06802198
Pinzgauer	0.04608973	0.02692809	0.03902701	0.05078905	0.06509787	0.06255126
Pinzgauer X Scottish Highland	0.07029871	0.05539606	0.05932752	0.06268249	0.08309298	0.08504145
Pinzgauer X Simmentaler	0.06475692	0.03835895	0.05457793	0.07334531	0.09164552	0.08139099
Pinzgauer: Moelltaler	0.0458069	0.04956436	0.04449626	0.04722149	0.05216186	0.05954109
Pinzgauer: Pustertaler	0.08439979	0.06595957	0.07031532	0.08974922	0.10014325	0.0982495
Pirenaica	0.05151258	0.0352773	0.03889479	0.05648177	0.06274648	0.06699976
Red Poll	0.08033455	0.06074761	0.06475632	0.08449106	0.09649187	0.09494296
Maas-Rhein-Ijsselschlag	0.04310245	0.03800407	0.03295134	0.04676555	0.05214669	0.05848057
Danish Red	0.05038313	0.05615615	0.04254307	0.04514056	0.04447968	0.0625064
Angeln	0.05167729	0.04612214	0.04533556	0.05797922	0.06322968	0.06104988
Bohemian Red	0.03787231	0.04070603	0.03752901	0.04096201	0.04968191	0.05275484
Polish Red	0.03890568	0.06032597	0.04666443	0.04916075	0.03340395	0.05276478
Sanga	0.06944694	0.09129852	0.07215004	0.06458756	0.038611	0.07410639
Sardinian	0.05651618	0.06237641	0.05315468	0.04904321	0.05194901	0.06777168
Scheinfelder	0.04812267	0.06104304	0.04691077	0.0484355	0.05045257	0.05012848
Schoenhengster	0.04070212	0.04486137	0.04087818	0.04066798	0.04996459	0.05303795
Holland Black Pied	0.05678975	0.04460366	0.04481606	0.06100109	0.07157913	0.07131717

Andalusian Black	0.04810589	0.05876631	0.04612413	0.04494645	0.0481152	0.0525349
Sweden (Jaemtland or Vatterbotten)	0.07048696	0.0717683	0.06445527	0.05993102	0.06745728	0.07697242
Scottish Highland	0.06974461	0.04190533	0.05827489	0.07451502	0.08911941	0.08207946
Sicilian	0.07266254	0.05088156	0.06711512	0.06100253	0.09139259	0.08811619
Småland	0.04773652	0.0669653	0.05307461	0.05578396	0.04343429	0.0493143
South Devon	0.04637892	0.05136413	0.04510115	0.04282319	0.04349876	0.05741617
Spanish Fighting Cattle	0.06725167	0.05301209	0.05486278	0.07022655	0.08440074	0.07707167
Buchara Grey	0.10626364	0.13596732	0.11582919	0.10838383	0.07611923	0.10673878
Hungarian Grey	0.04704383	0.03575106	0.04131901	0.05339783	0.0673259	0.06066757
Hungarian Grey X Bern Red Pied	0.07250766	0.0563863	0.06889046	0.08047381	0.09458806	0.08470676
Sudeten	0.04613291	0.04124561	0.04462415	0.05250364	0.06023784	0.06798433
Sudeten X Simmental	0.05821462	0.03921067	0.0457746	0.06493391	0.07425368	0.07731591
Sudeten X Tesstal?	0.05630586	0.04372402	0.05239115	0.05468513	0.07110492	0.0719207
Tarentaise	0.05492997	0.04503589	0.04177701	0.05777691	0.07046544	0.0689476
Telemark	0.04728155	0.04816155	0.04375428	0.05137326	0.05256389	0.05883728
Tudanca	0.05612909	0.05988072	0.05238812	0.0549763	0.05327252	0.07000977
Tuxer	0.09786598	0.0668155	0.07998069	0.10174542	0.11843484	0.11520804
Heck	0.04863234	0.04269203	0.04454391	0.05690372	0.06025385	0.06075216
Niata	0.21841139	0.18287529	0.206752	0.22955942	0.24769697	0.2315688
Veredelter Landschlag		0.05341791	0.0482382	0.05394123	0.05557017	0.05070279
Vogtlaender	0.05341791		0.04178714	0.05848979	0.07907604	0.07234708
Waldviertler X Scheinfelder	0.0482382	0.04178714		0.05058373	0.06025971	0.06546736
Watussi	0.05394123	0.05848979	0.05058373		0.05435075	0.05943559
Zebu	0.05557017	0.07907604	0.06025971	0.05435075		0.0549839
Zebu (Africa)	0.05070279	0.07234708	0.06546736	0.05943559	0.0549839	
Zebu (Asian)	0.05543076	0.05627299	0.06005781	0.0639929	0.06201686	0.06585701
Zebu (Indian)	0.05934383	0.0756074	0.06249885	0.05712058	0.03369076	0.06194345
Zillertaler	0.07031489	0.0370188	0.05576963	0.07743688	0.09192782	0.08721118
	Zebu (Asian)	Zebu (Indian)	Zillertaler			
Ayrshire	0.05310591	0.0702599	0.05075463			
Blondvieh: Carinthian	0.05480562	0.0495255	0.05929228			
Blondvieh: Lavanttaler	0.0563118	0.04967786	0.05790197			
Blondvieh: Mariahofer	0.05812017	0.06659112	0.04280034			

Blondvieh: Murbodner	0.05199334	0.05470104	0.05450565
Blondvieh: Waldviertler	0.05517691	0.04628484	0.05831793
Bos primigenius (Aurochs)	0.07939341	0.07110945	0.08006397
Brazilian	0.05977788	0.06492214	0.05277231
Montafon	0.05674525	0.04539981	0.05753961
Buša	0.05955969	0.05003073	0.06356017
Chianina	0.0549719	0.04525843	0.06797234
Chillingham	0.08092564	0.08024363	0.06990026
Devon	0.04480694	0.05612818	0.05138418
Egerlaender	0.06609164	0.05293166	0.07335994
Hérens	0.06800851	0.07948352	0.04261535
Fjällko	0.05847066	0.05355058	0.06738378
Bern Red Pied	0.05563374	0.0490313	0.05899267
Freiburg Red Pied	0.06318753	0.06334873	0.06092899
Simmental	0.05574051	0.06621872	0.04042996
South Moravian Red Pied	0.0572934	0.05626586	0.04941387
Rubia Gallega	0.07412051	0.05169998	0.08991773
Allgaeuer Grey	0.06854335	0.07214186	0.05162818
Krainer Grey	0.06114882	0.06532857	0.04975503
Oberinntaler Grey	0.05678138	0.05944954	0.04831563
Haná-Berne	0.06212372	0.05246812	0.06701203
Iceland	0.06063769	0.07422879	0.05893648
Jersey	0.06469653	0.09250924	0.05527222
Jersey (polled)	0.06575601	0.08551404	0.04856614
Jutland	0.06334888	0.04938252	0.0751891
Kampeten: Styrian Bergscheck	0.05735414	0.05769999	0.0453463
Kampeten: Welser Schecken	0.06707126	0.05630159	0.06718179
Kerry	0.05798711	0.05337604	0.06235916
Cretan	0.05578213	0.05422269	0.07174291
Kuhlaender	0.04848799	0.04699666	0.05412765
Landschlag von Warnsdorf	0.06349601	0.07119827	0.0617426
Landschlag von Winkelsdorf	0.07177995	0.05289867	0.08288851
Landschlag X Simmental	0.06287692	0.04876466	0.07227381

Limousin	0.04615196	0.06872085	0.04654102
Moravian Landschlag	0.05334081	0.06162309	0.05127947
Normande	0.05162916	0.07088812	0.04719363
North Wales	0.04857515	0.04937395	0.0537662
White Park	0.06782318	0.06864225	0.07242997
Pinzgauer	0.04881184	0.05997134	0.04125113
Pinzgauer X Scottish Highland	0.07478445	0.07817723	0.06042988
Pinzgauer X Simmentaler	0.06364088	0.08650298	0.0439769
Pinzgauer: Moelltaler	0.0510689	0.04310434	0.05784772
Pinzgauer: Pustertaler	0.08152895	0.09269184	0.04884842
Pirenaica	0.05018705	0.06021756	0.04449393
Red Poll	0.08540272	0.09180316	0.05367539
Maas-Rhein-Ijsselschlag	0.04740082	0.05370648	0.05094394
Danish Red	0.05909346	0.04839428	0.07222405
Angeln	0.06161924	0.06186807	0.04985848
Bohemian Red	0.05423916	0.04962465	0.0571053
Polish Red	0.05530938	0.03935037	0.07236065
Sanga	0.08024642	0.04905263	0.10213818
Sardinian	0.05878771	0.04981456	0.07639703
Scheinfelder	0.06114959	0.0513121	0.0721494
Schoenhengster	0.05719426	0.04815304	0.05827318
Holland Black Pied	0.06314435	0.06626308	0.04469871
Andalusian Black	0.06312054	0.04800194	0.06695781
Sweden (Jaemtland or Vatterbotten)	0.08101122	0.07918716	0.08993977
Scottish Highland	0.07412975	0.08363677	0.03587191
Sicilian	0.07686251	0.08627507	0.06269382
Småland	0.06359087	0.05501351	0.0796255
South Devon	0.04969324	0.04459459	0.06736383
Spanish Fighting Cattle	0.07766413	0.08136256	0.05109725
Buchara Grey	0.1122263	0.07517239	0.14029012
Hungarian Grey	0.05025934	0.06120434	0.04907272
Hungarian Grey X Bern Red Pied	0.06888739	0.08600224	0.05971245
Sudeten	0.05705628	0.06080405	0.05848709

Sudeten X Simmental	0.0631518	0.07724613	0.05546119
Sudeten X Tesstal?	0.0609244	0.06526532	0.0575406
Tarentaise	0.06618855	0.06938103	0.04733109
Telemark	0.05300663	0.05444816	0.05666412
Tudanca	0.06027731	0.04956812	0.06314797
Tuxer	0.0973639	0.11153585	0.04998966
Heck	0.05243446	0.06084161	0.05196455
Niata	0.20864069	0.23925479	0.17110927
Veredelter Landschlag	0.05543076	0.05934383	0.07031489
Vogtlaender	0.05627299	0.0756074	0.0370188
Waldviertler X Scheinfelder	0.06005781	0.06249885	0.05576963
Watussi	0.0639929	0.05712058	0.07743688
Zebu	0.06201686	0.03369076	0.09192782
Zebu (Africa)	0.06585701	0.06194345	0.08721118
Zebu (Asian)		0.05791325	0.06702839
Zebu (Indian)	0.05791325		0.08382127
Zillertaler	0.06702839	0.08382127	

Supplementary Table 12: Definition of the landmarks used on the lower jaw in this study.

LM	Definition
1	Medial boarder between bone and root of I1 sin.
2	Lateralmost point of the incisor-shovel sin.
3	Posterior boarder of the mental foramen sin.
4	Ventralmost point of the posterior beginning of the symphysis sin.
5	Anterior boarder mandibel and premolar 2 sin
6	Posterior boarder mandibel and molar 3 sin
7	Middlepoint of curvature of the angulus sin.
8	Ventralmost midpoint of the mandibular notch sin.
9	Lateralmost point of the condylar process sin.
10	Medialmost point of the condylar process sin.
11	Tip of thr coronoid process sin.
12	Ventral boarder of the mandibular foramen sin.

Supplementary Table 13: Procrustes distances among lower jaws from cattle breeds represented in morphospace.

	Ayrshire	Blondvieh: Carinthian	Blondvieh: Lavanttaler	Blondvieh: Mariahofer	Blondvieh: Murbodner	Blondvieh: Waldviertler
Ayrshire		0.02429243	0.03569847	0.03387412	0.02515366	0.02208665
Blondvieh: Carinthian	0.02429243		0.0251741	0.03689549	0.01838436	0.02853411
Blondvieh: Lavanttaler	0.03569847	0.0251741		0.04268506	0.02903718	0.03569526
Blondvieh: Mariahofer	0.03387412	0.03689549	0.04268506		0.03695584	0.02967699
Blondvieh: Murbodner	0.02515366	0.01838436	0.02903718	0.03695584		0.03012767
Blondvieh: Waldviertler	0.02208665	0.02853411	0.03569526	0.02967699	0.03012767	
Bos primigenius (Aurochs)	0.06791667	0.06661593	0.0703849	0.08758241	0.06641901	0.07730602
Montafon	0.02245857	0.0322749	0.04045911	0.03595874	0.0346612	0.02844752
Buša	0.03039321	0.03136813	0.03533274	0.03795467	0.03084482	0.03119042
Devon	0.01796509	0.02249791	0.03172554	0.02666152	0.02130642	0.02250737
Egerlaender	0.02630985	0.02327783	0.02643268	0.0260126	0.02397431	0.02544332
Bern Red Pied	0.03539837	0.02934611	0.04090368	0.05244141	0.02624057	0.04515901
Simmental	0.0241662	0.01538109	0.02679297	0.03477909	0.01958467	0.03138109
South Moravian Red Pied	0.02135052	0.02995466	0.0400554	0.03253932	0.02570137	0.02763821
Rubia Gallega	0.03314037	0.03622608	0.04658761	0.05292906	0.03588809	0.04208807
Krainer Grey	0.03691877	0.02779987	0.04255894	0.04913573	0.03064205	0.03972122
Oberinntaler Grey	0.02278382	0.03921399	0.04744489	0.03172929	0.0390355	0.02528104
Guernsey	0.03458542	0.03936381	0.04541524	0.03611163	0.03769574	0.03620581
Iceland	0.02714866	0.03533894	0.04069188	0.03458962	0.03595968	0.03068999
Jersey	0.04073217	0.05024173	0.05176862	0.03033575	0.04916311	0.03664665
Kampeten: Styrian Bergscheck	0.01762367	0.02102681	0.03025091	0.02742797	0.02136101	0.01804816
Kampeten: Welser Schecken	0.02960982	0.02153627	0.02966093	0.04903527	0.02695801	0.03671276
Kerry	0.01561973	0.02536641	0.03456707	0.03624807	0.02553012	0.01727216
Cretan	0.04139895	0.04960716	0.06072142	0.06456701	0.04824666	0.05275969
Kuhlaender	0.03032075	0.02547851	0.03218497	0.03044662	0.02500452	0.03205819
Limousin	0.03110276	0.03130535	0.03620033	0.02748982	0.02958387	0.0334418
Moravian Landschlag	0.02887919	0.02827003	0.03551944	0.03756002	0.03126182	0.03481937
Normande	0.03674045	0.04771467	0.05469848	0.02643556	0.04458371	0.03646524
North Wales	0.02501707	0.02425674	0.03252055	0.03532284	0.0257982	0.03174944
White Park	0.04538719	0.05017195	0.05273368	0.0375368	0.05235692	0.04674696
Pasiega	0.04639331	0.04688545	0.05531899	0.06023911	0.05011106	0.05247276
Pinzgauer	0.03611017	0.03217254	0.0373694	0.03528647	0.03148413	0.03687612
Pinzgauer: Moelltaler	0.0316514	0.02649579	0.03363051	0.02945541	0.02658537	0.03427539
Pirenaica	0.0223481	0.02776444	0.03450442	0.02144712	0.02905276	0.02159358
Maas-Rhein-Ijsselschlag	0.01430081	0.02239229	0.03514495	0.03231297	0.02235996	0.02740803
Danish Red	0.02883581	0.02425955	0.03353436	0.03602634	0.02659708	0.03098475
Angeln	0.03594893	0.03497461	0.03498754	0.0320897	0.02989729	0.03136853
Bohemian Red	0.01772635	0.01871309	0.02708604	0.03353723	0.02249009	0.01837522
Polish Red	0.03198718	0.03630214	0.04509226	0.0463096	0.03390861	0.03012947
Sardinian	0.03356758	0.03013031	0.03529458	0.04531251	0.03481891	0.0425747
Scheinfelder	0.04881791	0.03772419	0.04418294	0.0588636	0.03965514	0.05453945
Schoenhengster	0.03187033	0.0274133	0.03754522	0.03074477	0.03241748	0.02618348
Holland Black Pied	0.03171618	0.02628019	0.03410052	0.03089636	0.02610446	0.03261951
Andalusian Black	0.0255727	0.02576462	0.0354445	0.04272334	0.02700889	0.02663795
Scottish Highland	0.02896251	0.02703756	0.0369443	0.04443108	0.02872712	0.03782807
South Devon	0.02413912	0.02967474	0.04007943	0.04278457	0.0271152	0.03297259

Spanish Fighting Cattle	0.03490936	0.03351006	0.04471848	0.04941174	0.03413943	0.04138981
Buchara Grey	0.03914046	0.03399658	0.0424092	0.05476353	0.03219511	0.04787483
Kalmuek Steppe	0.03421003	0.04171071	0.05250256	0.05155455	0.03507955	0.04218274
Hungarian Grey	0.03126007	0.03082538	0.03955788	0.04774819	0.02313487	0.03639602
Sudeten	0.02397289	0.02766839	0.03297027	0.03223172	0.03058786	0.02120089
Sudeten X Tesstal?	0.04387617	0.04445254	0.04896189	0.04001524	0.04589573	0.04423636
Tarentaise	0.03121708	0.03397565	0.03856438	0.02309781	0.03409581	0.03067981
Telemark	0.0149141	0.0279413	0.03880899	0.03401272	0.02957539	0.02151054
Tesstal	0.03089871	0.02990902	0.03066292	0.029545	0.02475287	0.03229446
Tudanca	0.0340265	0.03024596	0.0389928	0.05408106	0.03199767	0.0371226
Tuxer	0.08419934	0.08468212	0.08553392	0.07541658	0.08295407	0.08088856
Heck	0.04958076	0.03903667	0.0434125	0.06352517	0.04224447	0.05548117
Niata	0.12089517	0.1309297	0.13378336	0.10309312	0.1260237	0.11898871
Vogtlaender	0.04578862	0.0518958	0.05529858	0.03442327	0.05024192	0.04485818
Walchshofer	0.05028606	0.04949138	0.05726462	0.06416943	0.05074389	0.05659412
Watussi	0.03805688	0.04214962	0.06066164	0.05384713	0.04445135	0.04979337
Zebu	0.02785785	0.0313872	0.03248779	0.03884823	0.03388831	0.03365487
Zebu (India)	0.03109969	0.03713495	0.03819835	0.04220466	0.03403192	0.0331239
Zillertaler	0.04793925	0.04761986	0.04783296	0.0388128	0.05180469	0.04464436

	Bos primigenius (Aurochs)	Montafon	Buša	Devon	Egerlaender	Bern Red Pied
Ayrshire	0.06791667	0.02245857	0.03039321	0.01796509	0.02630985	0.03539837
Blondvieh: Carinthian	0.06661593	0.0322749	0.03136813	0.02249791	0.02327783	0.02934611
Blondvieh: Lavanttaler	0.0703849	0.04045911	0.03533274	0.03172554	0.02643268	0.04090368
Blondvieh: Mariahofer	0.08758241	0.03595874	0.03795467	0.02666152	0.0260126	0.05244141
Blondvieh: Murbodner	0.06641901	0.0346612	0.03084482	0.02130642	0.02397431	0.02624057
Blondvieh: Waldviertler	0.07730602	0.02844752	0.03119042	0.02250737	0.02544332	0.04515901
Bos primigenius (Aurochs)		0.07077486	0.06155161	0.07431305	0.07794852	0.06258681
Montafon	0.07077486		0.03308453	0.02984547	0.03207852	0.04690475
Buša	0.06155161	0.03308453		0.0353219	0.03067879	0.04422153
Devon	0.07431305	0.02984547	0.0353219		0.02075706	0.03349527
Egerlaender	0.07794852	0.03207852	0.03067879	0.02075706		0.04139736
Bern Red Pied	0.06258681	0.04690475	0.04422153	0.03349527	0.04139736	
Simmental	0.06690316	0.03329122	0.03188786	0.02023449	0.0242477	0.02687428
South Moravian Red Pied	0.06780619	0.02957864	0.02972817	0.02102388	0.02862831	0.036493
Rubia Gallega	0.0537728	0.03210093	0.0428509	0.03799319	0.04608148	0.0389789
Krainer Grey	0.07100669	0.04756209	0.04314438	0.03340385	0.03761533	0.0376375
Oberinntaler Grey	0.08021171	0.02209181	0.03810077	0.02675272	0.03252585	0.05046063
Guernsey	0.07923576	0.04214113	0.03546747	0.03233295	0.03283182	0.05084647
Iceland	0.06464286	0.02831397	0.0259789	0.0315809	0.03579855	0.04914399
Jersey	0.0993544	0.04620464	0.0501245	0.03601006	0.03759558	0.06555615
Kampeten: Styrian Bergscheck	0.07265837	0.02848371	0.02786925	0.01659617	0.01514255	0.03717238
Kampeten: Welser Schecken	0.05407228	0.03796806	0.03497734	0.03196188	0.03582305	0.02813042
Kerry	0.06509034	0.02640233	0.02708344	0.02084958	0.0270735	0.03687791
Cretan	0.0556343	0.03963782	0.04685897	0.05171443	0.05908888	0.0472887
Kuhlaender	0.08361948	0.03857787	0.04088205	0.02159952	0.02083635	0.0393878
Limousin	0.08208949	0.04297061	0.04185657	0.0199744	0.02750267	0.03993617
Moravian Landschlag	0.08190406	0.04178173	0.04161052	0.02683108	0.02947596	0.04437833
Normande	0.09070326	0.04470765	0.04764582	0.03001403	0.03650928	0.05381402
North Wales	0.07192595	0.03895124	0.03890962	0.01987533	0.02893311	0.0360095

White Park	0.08250841	0.05068475	0.04121433	0.04528197	0.04620848	0.06057495
Pasiega	0.06456872	0.05614626	0.0549707	0.04440908	0.05540004	0.04849805
Pinzgauer	0.08985792	0.04908071	0.04695444	0.02634061	0.02939896	0.04443671
Pinzgauer: Moelltaler	0.07923475	0.03859526	0.04015353	0.02097083	0.02578129	0.03542329
Pirenaica	0.0764082	0.0274508	0.03342395	0.01678907	0.0221742	0.04544308
Maas-Rhein-Ijsselschlag	0.06709407	0.02928132	0.03277756	0.01497369	0.02792848	0.02840305
Danish Red	0.0632905	0.03912487	0.03383672	0.02447707	0.0275893	0.03585875
Angeln	0.07879622	0.04027787	0.03723333	0.02928483	0.03390657	0.04329604
Bohemian Red	0.06370072	0.02465363	0.02502374	0.02032575	0.0213625	0.03640094
Polish Red	0.06259703	0.03312725	0.03102274	0.03584221	0.0390245	0.0460882
Sardinian	0.05739873	0.04005204	0.0384278	0.03234172	0.03537999	0.0367579
Scheinfelder	0.05095277	0.05227388	0.04257843	0.04738784	0.04849727	0.03846517
Schoenhengster	0.08010364	0.04041044	0.03418105	0.02818207	0.027975	0.04515241
Holland Black Pied	0.07821854	0.04306442	0.03769724	0.02459696	0.02962156	0.03538432
Andalusian Black	0.05806114	0.02775177	0.03139244	0.02816054	0.03284447	0.03679061
Scottish Highland	0.05763299	0.0383097	0.03857913	0.02799683	0.03934329	0.03060475
South Devon	0.06235569	0.0358527	0.04159905	0.02327638	0.03551851	0.03048058
Spanish Fighting Cattle	0.04582249	0.03934371	0.03050655	0.03925941	0.0423444	0.04002143
Buchara Grey	0.05734248	0.04622007	0.04489177	0.03881118	0.04663075	0.03555405
Kalmuek Steppe	0.0679667	0.03967135	0.03666577	0.03968988	0.04595589	0.04071457
Hungarian Grey	0.06676574	0.0369421	0.04021527	0.02980986	0.03786497	0.02949592
Sudeten	0.08339522	0.03440332	0.03795404	0.0231666	0.02225162	0.04601969
Sudeten X Tesstal?	0.09544603	0.05083834	0.05617602	0.03811584	0.04253272	0.05887529
Tarentaise	0.09035712	0.03983784	0.03896326	0.02554563	0.02131807	0.04689327
Telemark	0.06859943	0.02667982	0.03148689	0.0205269	0.0295801	0.04075859
Tesstal	0.07961847	0.04176057	0.035095	0.02382684	0.02565859	0.04157573
Tudanca	0.05561256	0.04277802	0.0335118	0.0380025	0.04223786	0.02982827
Tuxer	0.14076954	0.09624793	0.09175262	0.07793849	0.07430769	0.09267555
Heck	0.05551526	0.05809892	0.04697567	0.04921778	0.05396975	0.03416278
Niata	0.17912937	0.12303935	0.13044592	0.11472742	0.11593429	0.13375176
Vogtlaender	0.10615265	0.05627892	0.0581231	0.03812064	0.03919285	0.06241116
Walchshofer	0.05551558	0.04862718	0.05703866	0.05016564	0.06055386	0.05175432
Watussi	0.07743216	0.0492844	0.05068023	0.04262925	0.04982544	0.04444482
Zebu	0.06601321	0.03321541	0.02821937	0.03192958	0.03498815	0.04057932
Zebu (India)	0.06511335	0.03171689	0.03018187	0.03191265	0.03732607	0.0407217
Zillertaler	0.09981453	0.05705985	0.05542943	0.04114391	0.04108369	0.06097118

	Simmental	South Moravian Red Pied	Rubia Gallega	Krainer Grey	Oberinntaler Grey	Guernsey
Ayrshire	0.0241662	0.02135052	0.03314037	0.03691877	0.02278382	0.03458542
Blondvieh: Carinthian	0.01538109	0.02995466	0.03622608	0.02779987	0.03921399	0.03936381
Blondvieh: Lavanttaler	0.02679297	0.0400554	0.04658761	0.04255894	0.04744489	0.04541524
Blondvieh: Mariahofer	0.03477909	0.03253932	0.05292906	0.04913573	0.03172929	0.03611163
Blondvieh: Murbodner	0.01958467	0.02570137	0.03588809	0.03064205	0.0390355	0.03769574
Blondvieh: Waldviertler	0.03138109	0.02763821	0.04208807	0.03972122	0.02528104	0.03620581
Bos primigenius (Aurochs)	0.06690316	0.06780619	0.0537728	0.07100669	0.08021171	0.07923576
Montafon	0.03329122	0.02957864	0.03210093	0.04756209	0.02209181	0.04214113
Buša	0.03188786	0.02972817	0.0428509	0.04314438	0.03810077	0.03546747
Devon	0.02023449	0.02102388	0.03799319	0.03340385	0.02675272	0.03233295
Egerlaender	0.0242477	0.02862831	0.04608148	0.03761533	0.03252585	0.03283182
Bern Red Pied	0.02687428	0.036493	0.0389789	0.0376375	0.05046063	0.05084647

Simmental		0.02751847	0.03782586	0.03009972	0.03739852	0.0367656
South Moravian Red Pied	0.02751847		0.03594076	0.03831309	0.0265932	0.02977231
Rubia Gallega	0.03782586	0.03594076		0.04450778	0.04057455	0.05243866
Krainer Grey	0.03009972	0.03831309	0.04450778		0.04797757	0.03857652
Oberinntaler Grey	0.03739852	0.0265932	0.04057455	0.04797757		0.03586498
Guernsey	0.0367656	0.02977231	0.05243866	0.03857652	0.03586498	
Iceland	0.03628455	0.02775015	0.03665189	0.04545841	0.03165289	0.03701056
Jersey	0.04810518	0.04248404	0.06326967	0.05306929	0.03494132	0.03339594
Kampeten: Styrian Bergscheck	0.02258893	0.02463148	0.04015475	0.03525919	0.02746892	0.03274995
Kampeten: Welser Schecken	0.02489264	0.03576963	0.03217741	0.03823527	0.04602675	0.05039278
Kerry	0.02574652	0.02286947	0.03308984	0.03399833	0.0259442	0.03428834
Cretan	0.04973713	0.04597942	0.02973049	0.05780672	0.0469818	0.06143363
Kuhlaender	0.02368775	0.034634	0.04846229	0.03579122	0.03742163	0.03720355
Limousin	0.02621899	0.03137072	0.05013945	0.03768223	0.03889712	0.03657982
Moravian Landschlag	0.02888546	0.03639474	0.04922331	0.03391032	0.0394101	0.03553702
Normande	0.04302659	0.03297258	0.05681438	0.05303394	0.03185538	0.03698748
North Wales	0.02700918	0.02978689	0.04165697	0.03302588	0.03908926	0.03985951
White Park	0.04751321	0.0456252	0.06460815	0.06097142	0.04985029	0.04778222
Pasiega	0.04445332	0.04532775	0.04933405	0.04627833	0.05469211	0.04761786
Pinzgauer	0.03093095	0.03927228	0.0571743	0.03393898	0.04488371	0.03810925
Pinzgauer: Moelltaler	0.02128757	0.03080123	0.04486861	0.03777704	0.03652397	0.03860151
Pirenaica	0.02808784	0.02530314	0.03971956	0.03696634	0.02487027	0.02943366
Maas-Rhein-Ijsselschlag	0.01920698	0.02144237	0.0322595	0.03400955	0.02978355	0.03485554
Danish Red	0.0254804	0.02967589	0.04003669	0.03324067	0.04151746	0.03921961
Angeln	0.03245037	0.03495191	0.04526975	0.04200067	0.04109922	0.042652
Bohemian Red	0.02244967	0.02433984	0.03220492	0.03424133	0.02952424	0.03702424
Polish Red	0.03868838	0.02824315	0.03469259	0.03887537	0.03554722	0.04062176
Sardinian	0.02720113	0.03221306	0.04004627	0.04361686	0.04500481	0.04041099
Scheinfelder	0.03441049	0.04580596	0.0465274	0.0416186	0.0594266	0.05197646
Schoenhengster	0.02843204	0.03500159	0.05142981	0.03309932	0.0394366	0.03588343
Holland Black Pied	0.02762217	0.03260572	0.04947482	0.03834103	0.04358883	0.04332262
Andalusian Black	0.02999574	0.02459787	0.02671297	0.03917608	0.03311915	0.04190828
Scottish Highland	0.02467769	0.02904328	0.0325672	0.03824232	0.04228597	0.04369519
South Devon	0.02987513	0.0288154	0.03108496	0.0370893	0.03755982	0.04521738
Spanish Fighting Cattle	0.03535865	0.03305502	0.03369039	0.03987745	0.04511607	0.04334565
Buchara Grey	0.03504841	0.04124789	0.03579185	0.03211145	0.0534112	0.04868112
Kalmuek Steppe	0.0402701	0.03228713	0.0371746	0.04093972	0.03937179	0.04311018
Hungarian Grey	0.02934926	0.03041644	0.03009533	0.03395989	0.03805237	0.04386379
Sudeten	0.03164301	0.03398786	0.04819353	0.03705863	0.03305646	0.03569112
Sudeten X Tesstal?	0.04652839	0.04648608	0.05546055	0.04936129	0.04835056	0.05023466
Tarentaise	0.03092977	0.03398008	0.05687519	0.04566354	0.03378289	0.0316768
Telemark	0.02991327	0.02220508	0.03450921	0.03450518	0.02492905	0.03300593
Tesstal	0.02778052	0.03213434	0.04849126	0.03717654	0.04074383	0.03548026
Tudanca	0.03242893	0.03629011	0.03772819	0.04014353	0.04755758	0.04947882
Tuxer	0.08249752	0.0876061	0.11016042	0.08263318	0.08645804	0.07780713
Heck	0.03724044	0.05003635	0.04867264	0.04472231	0.06375246	0.05885215
Niata	0.12449251	0.11803917	0.14046524	0.13513592	0.1101461	0.11535326
Vogtlaender	0.04768507	0.04876391	0.07213813	0.05545165	0.04553236	0.04297022
Walchshofer	0.04920412	0.04740323	0.02917666	0.05282032	0.05570535	0.06224884
Watussi	0.04048703	0.04229468	0.05237859	0.04165864	0.04650161	0.04481144

Zebu	0.02897954	0.0363308	0.04497314	0.04938857	0.03889062	0.04406533
Zebu (India)	0.03296851	0.02833444	0.03800656	0.04987745	0.03468945	0.04509092
Zillertaler	0.04485788	0.05173134	0.07263585	0.0542334	0.05197931	0.04659155

	Iceland	Jersey	Kampeten: Styrian Bergscheck	Kampeten: Welser Schecken	Kerry	Cretan
Ayrshire	0.02714866	0.04073217	0.01762367	0.02960982	0.01561973	0.04139895
Blondvieh: Carinthian	0.03533894	0.05024173	0.02102681	0.02153627	0.02536641	0.04960716
Blondvieh: Lavanttaler	0.04069188	0.05176862	0.03025091	0.02966093	0.03456707	0.06072142
Blondvieh: Mariahofer	0.03458962	0.03033575	0.02742797	0.04903527	0.03624807	0.06456701
Blondvieh: Murbodner	0.03595968	0.04916311	0.02136101	0.02695801	0.02553012	0.04824666
Blondvieh: Waldviertler	0.03068999	0.03664665	0.01804816	0.03671276	0.01727216	0.05275969
Bos primigenius (Aurochs)	0.06464286	0.0993544	0.07265837	0.05407228	0.06509034	0.0556343
Montafon	0.02831397	0.04620464	0.02848371	0.03796806	0.02640233	0.03963782
Buša	0.0259789	0.0501245	0.02786925	0.03497734	0.02708344	0.04685897
Devon	0.0315809	0.03601006	0.01659617	0.03196188	0.02084958	0.05171443
Egerlaender	0.03579855	0.03759558	0.01514255	0.03582305	0.0270735	0.05908888
Bern Red Pied	0.04914399	0.06555615	0.03717238	0.02813042	0.03687791	0.0472887
Simmental	0.03628455	0.04810518	0.02258893	0.02489264	0.02574652	0.04973713
South Moravian Red Pied	0.02775015	0.04248404	0.02463148	0.03576963	0.02286947	0.04597942
Rubia Gallega	0.03665189	0.06326967	0.04015475	0.03217741	0.03308984	0.02973049
Krainer Grey	0.04545841	0.05306929	0.03525919	0.03823527	0.03399833	0.05780672
Oberinntaler Grey	0.03165289	0.03494132	0.02746892	0.04602675	0.0259442	0.0469818
Guernsey	0.03701056	0.03339594	0.03274995	0.05039278	0.03428834	0.06143363
Iceland		0.0423499	0.03199877	0.03826634	0.02912554	0.04473752
Jersey	0.0423499		0.03876705	0.06284066	0.04318444	0.07301639
Kampeten: Styrian Bergscheck	0.03199877	0.03876705		0.03077452	0.01679983	0.05117444
Kampeten: Welser Schecken	0.03826634	0.06284066	0.03077452		0.02902892	0.04525218
Kerry	0.02912554	0.04318444	0.01679983	0.02902892		0.04355789
Cretan	0.04473752	0.07301639	0.05117444	0.04525218	0.04355789	
Kuhlaender	0.04315813	0.03726532	0.02392006	0.04127422	0.03370796	0.0594822
Limousin	0.03701732	0.03543432	0.02858614	0.04060535	0.03516914	0.06325456
Moravian Landschlag	0.03892167	0.03578563	0.02903471	0.04140982	0.03535876	0.05885624
Normande	0.04095043	0.03121174	0.03515841	0.05539716	0.0390787	0.06562267
North Wales	0.03285492	0.04231081	0.02662547	0.03238176	0.02942815	0.05581489
White Park	0.03636874	0.04828807	0.04573259	0.05373082	0.04819308	0.0690253
Pasiega	0.04917429	0.06382487	0.04940692	0.04518015	0.04569232	0.05882273
Pinzgauer	0.04658078	0.03565629	0.03111995	0.04635207	0.03907979	0.06961568
Pinzgauer: Moelltaler	0.03972744	0.04163578	0.02699061	0.03895398	0.0346688	0.05646648
Pirenaica	0.02428736	0.02785485	0.02019181	0.03916096	0.02412282	0.05402133
Maas-Rhein-Ijsselschlag	0.02901512	0.0434462	0.02079288	0.02669857	0.02084662	0.04377493
Danish Red	0.03433996	0.05018874	0.02360889	0.02565621	0.02492056	0.05658231
Angeln	0.03570271	0.04147	0.03339285	0.04318231	0.03440409	0.05741044
Bohemian Red	0.0274604	0.04492066	0.01496569	0.02315296	0.01337134	0.04576704
Polish Red	0.03456176	0.05443361	0.03208745	0.03899887	0.02228415	0.04436271
Sardinian	0.03944999	0.05722115	0.03355725	0.03065348	0.03410617	0.05411429
Scheinfelder	0.04913151	0.07252842	0.04773393	0.03605226	0.04675866	0.05558959
Schoenhengster	0.0403754	0.04183021	0.02384157	0.03980979	0.02777449	0.06247423
Holland Black Pied	0.03831314	0.0450877	0.02802418	0.03669947	0.0344385	0.0600981
Andalusian Black	0.03259536	0.05434604	0.02556615	0.02621839	0.02002164	0.04151773
Scottish Highland	0.03169364	0.05493534	0.03370018	0.02607906	0.03152014	0.04438241

South Devon	0.03761576	0.05364912	0.02836852	0.02667299	0.02391656	0.04814409
Spanish Fighting Cattle	0.03551449	0.06222114	0.03572694	0.03331539	0.03048095	0.04143393
Buchara Grey	0.04326955	0.06291533	0.04334177	0.03547569	0.0398756	0.05080835
Kalmuek Steppe	0.0396005	0.05577869	0.04117034	0.04629089	0.03474451	0.03557693
Hungarian Grey	0.04105182	0.05471385	0.03448706	0.0342956	0.03073287	0.04015172
Sudeten	0.03806454	0.03317638	0.02038379	0.04004367	0.02540135	0.06082773
Sudeten X Tesstal?	0.0448202	0.0374918	0.04446012	0.05603431	0.04935459	0.07062942
Tarentaise	0.04270363	0.03089384	0.02365211	0.04700911	0.03518888	0.0653426
Telemark	0.02393495	0.03901259	0.02169065	0.0334656	0.01614277	0.04505389
Tesstal	0.0359134	0.03703022	0.02627402	0.04052774	0.03337936	0.06112591
Tudanca	0.04108059	0.06517128	0.0361188	0.02213023	0.02946594	0.04297006
Tuxer	0.09606604	0.06476777	0.07715699	0.09875561	0.08643493	0.11690308
Heck	0.05149561	0.07415606	0.05157002	0.03509498	0.04875987	0.05379088
Niata	0.12608732	0.09695754	0.11859688	0.14168939	0.1256003	0.1439893
Vogtlaender	0.05677464	0.02796115	0.04055211	0.06500112	0.04977432	0.08109787
Walchshofer	0.04576724	0.07240334	0.0570019	0.04586121	0.04948159	0.04681131
Watussi	0.05101837	0.05754675	0.04350083	0.05053342	0.04327941	0.04985556
Zebu	0.03096781	0.05130352	0.03017839	0.03059765	0.03081235	0.04810126
Zebu (India)	0.03360481	0.05387438	0.03387191	0.03307028	0.02698558	0.04432295
Zillertaler	0.05192123	0.03752346	0.04392934	0.0587314	0.051254	0.08141396

	Kuhlaender	Limousin	Moravian Landschlag	Normande	North Wales	White Park
Ayrshire	0.03032075	0.03110276	0.02887919	0.03674045	0.02501707	0.04538719
Blondvieh: Carinthian	0.02547851	0.03130535	0.02827003	0.04771467	0.02425674	0.05017195
Blondvieh: Lavanttaler	0.03218497	0.03620033	0.03551944	0.05469848	0.03252055	0.05273368
Blondvieh: Mariahofer	0.03044662	0.02748982	0.03756002	0.02643556	0.03532284	0.0375368
Blondvieh: Murbodner	0.02500452	0.02958387	0.03126182	0.04458371	0.0257982	0.05235692
Blondvieh: Waldviertler	0.03205819	0.0334418	0.03481937	0.03646524	0.03174944	0.04674696
Bos primigenius (Aurochs)	0.08361948	0.08208949	0.08190406	0.09070326	0.07192595	0.08250841
Montafon	0.03857787	0.04297061	0.04178173	0.04470765	0.03895124	0.05068475
Buša	0.04088205	0.04185657	0.04161052	0.04764582	0.03890962	0.04121433
Devon	0.02159952	0.0199744	0.02683108	0.03001403	0.01987533	0.04528197
Egerlaender	0.02083635	0.02750267	0.02947596	0.03650928	0.02893311	0.04620848
Bern Red Pied	0.0393878	0.03993617	0.04437833	0.05381402	0.0360095	0.06057495
Simmental	0.02368775	0.02621899	0.02888546	0.04302659	0.02700918	0.04751321
South Moravian Red Pied	0.034634	0.03137072	0.03639474	0.03297258	0.02978689	0.0456252
Rubia Gallega	0.04846229	0.05013945	0.04922331	0.05681438	0.04165697	0.06460815
Krainer Grey	0.03579122	0.03768223	0.03391032	0.05303394	0.03302588	0.06097142
Oberinntaler Grey	0.03742163	0.03889712	0.0394101	0.03185538	0.03908926	0.04985029
Guernsey	0.03720355	0.03657982	0.03553702	0.03698748	0.03985951	0.04778222
Iceland	0.04315813	0.03701732	0.03892167	0.04095043	0.03285492	0.03636874
Jersey	0.03726532	0.03543432	0.03578563	0.03121174	0.04231081	0.04828807
Kampeten: Styrian Bergscheck	0.02392006	0.02858614	0.02903471	0.03515841	0.02662547	0.04573259
Kampeten: Welser Schecken	0.04127422	0.04060535	0.04140982	0.05539716	0.03238176	0.05373082
Kerry	0.03370796	0.03516914	0.03535876	0.0390787	0.02942815	0.04819308
Cretan	0.0594822	0.06325456	0.05885624	0.06562267	0.05581489	0.0690253
Kuhlaender		0.02527385	0.02154837	0.03910659	0.02783733	0.0523971
Limousin	0.02527385		0.02679438	0.02963098	0.02557579	0.04007106
Moravian Landschlag	0.02154837	0.02679438		0.04339578	0.02639655	0.05136003
Normande	0.03910659	0.02963098	0.04339578		0.0407808	0.04065065

North Wales	0.02783733	0.02557579	0.02639655	0.0407808		0.04470978
White Park	0.0523971	0.04007106	0.05136003	0.04065065	0.04470978	
Pasiega	0.05486943	0.05013621	0.05219864	0.05695537	0.04582388	0.06105468
Pinzgauer	0.02112431	0.02373797	0.02159739	0.04185287	0.02548044	0.05294198
Pinzgauer: Moelltaler	0.01822877	0.02386185	0.02756172	0.03783682	0.02952579	0.05148968
Pirenaica	0.02544665	0.02546276	0.02759583	0.03037241	0.02303431	0.04153625
Maas-Rhein-Ijsselschlag	0.02952193	0.02421267	0.02957434	0.03439345	0.02323049	0.04339132
Danish Red	0.03574696	0.0310901	0.03882559	0.04053918	0.02545456	0.04346641
Angeln	0.03196469	0.02897271	0.03799347	0.04181794	0.03266322	0.0469229
Bohemian Red	0.03032514	0.03342766	0.03340191	0.04178955	0.02566904	0.04602188
Polish Red	0.0476028	0.04940129	0.04937424	0.05123405	0.04178757	0.05929866
Sardinian	0.0412536	0.04011546	0.04300567	0.04933522	0.03679109	0.05161502
Scheinfelder	0.05098918	0.04959716	0.05377976	0.06445199	0.04862645	0.05966795
Schoenhengster	0.03151861	0.03432302	0.03591898	0.04261905	0.03133318	0.04589245
Holland Black Pied	0.02735592	0.02398611	0.03226059	0.0402889	0.01984287	0.0407209
Andalusian Black	0.03968118	0.04271922	0.04253548	0.04866985	0.03444794	0.0558448
Scottish Highland	0.03932611	0.0327975	0.0376325	0.0474897	0.02932192	0.04819947
South Devon	0.03952962	0.03523338	0.04150934	0.04238022	0.02753732	0.05422368
Spanish Fighting Cattle	0.04794363	0.04910565	0.04813428	0.05468203	0.04112135	0.05415258
Buchara Grey	0.04553889	0.0445798	0.04490897	0.06110787	0.03362315	0.06203673
Kalmuek Steppe	0.04523109	0.04834416	0.04492247	0.05228031	0.04317334	0.0614988
Hungarian Grey	0.03471652	0.03986856	0.03858321	0.05108229	0.03561647	0.06344002
Sudeten	0.02652639	0.03222877	0.0277049	0.04079325	0.02586102	0.04986675
Sudeten X Tesstal?	0.03756581	0.03410632	0.03180853	0.04568703	0.03528236	0.05662172
Tarentaise	0.02224404	0.02697449	0.02894966	0.03017258	0.03497432	0.0435876
Telemark	0.03522015	0.03295981	0.03333012	0.03579975	0.02325492	0.0430701
Tesstal	0.02505193	0.02204672	0.02583105	0.04000938	0.02545364	0.0471593
Tudanca	0.04708826	0.04739572	0.0480409	0.05749484	0.03956788	0.05579825
Tuxer	0.06721026	0.07205849	0.06897989	0.07622278	0.07911577	0.08960288
Heck	0.0520696	0.05050094	0.05215396	0.06869012	0.0454726	0.05984848
Niata	0.11260735	0.10958379	0.11880823	0.09430132	0.12447287	0.11708387
Vogtlaender	0.03426896	0.03480628	0.03842489	0.03303114	0.04411805	0.05331933
Walchshofer	0.0607834	0.05752096	0.05915888	0.06615691	0.0520773	0.07196865
Watussi	0.04431106	0.04946307	0.04254635	0.05250886	0.04417941	0.05900575
Zebu	0.03895842	0.03691648	0.03920114	0.04642298	0.03532138	0.03662139
Zebu (India)	0.04440114	0.04213608	0.04860304	0.04517114	0.04100439	0.04796784
Zillertaler	0.03970672	0.03164435	0.04041938	0.04208035	0.04311348	0.04041346

	Pasiega	Pinzgauer	Pinzgauer: Moelltaler	Pirenaica	Maas-Rhein-Ijsselschlag	Danish Red
Ayrshire	0.04639331	0.03611017	0.0316514	0.0223481	0.01430081	0.02883581
Blondvieh: Carinthian	0.04688545	0.03217254	0.02649579	0.02776444	0.02239229	0.02425955
Blondvieh: Lavanttaler	0.05531899	0.0373694	0.03363051	0.03450442	0.03514495	0.03353436
Blondvieh: Mariahofer	0.06023911	0.03528647	0.02945541	0.02144712	0.03231297	0.03602634
Blondvieh: Murbodner	0.05011106	0.03148413	0.02658537	0.02905276	0.02235996	0.02659708
Blondvieh: Waldviertler	0.05247276	0.03687612	0.03427539	0.02159358	0.02740803	0.03098475
Bos primigenius (Aurochs)	0.06456872	0.08985792	0.07923475	0.0764082	0.06709407	0.0632905
Montafon	0.05614626	0.04908071	0.03859526	0.0274508	0.02928132	0.03912487
Buša	0.0549707	0.04695444	0.04015353	0.03342395	0.03277756	0.03383672
Devon	0.04440908	0.02634061	0.02097083	0.01678907	0.01497369	0.02447707

Egerlaender	0.05540004	0.02939896	0.02578129	0.0221742	0.02792848	0.0275893
Bern Red Pied	0.04849805	0.04443671	0.03542329	0.04544308	0.02840305	0.03585875
Simmental	0.04445332	0.03093095	0.02128757	0.02808784	0.01920698	0.0254804
South Moravian Red Pied	0.04532775	0.03927228	0.03080123	0.02530314	0.02144237	0.02967589
Rubia Gallega	0.04933405	0.0571743	0.04486861	0.03971956	0.0322595	0.04003669
Krainer Grey	0.04627833	0.03393898	0.03777704	0.03696634	0.03400955	0.03324067
Oberinntaler Grey	0.05469211	0.04488371	0.03652397	0.02487027	0.02978355	0.04151746
Guernsey	0.04761786	0.03810925	0.03860151	0.02943366	0.03485554	0.03921961
Iceland	0.04917429	0.04658078	0.03972744	0.02428736	0.02901512	0.03433996
Jersey	0.06382487	0.03565629	0.04163578	0.02785485	0.0434462	0.05018874
Kampeten: Styrian Bergscheck	0.04940692	0.03111995	0.02699061	0.02019181	0.02079288	0.02360889
Kampeten: Welser Schecken	0.04518015	0.04635207	0.03895398	0.03916096	0.02669857	0.02565621
Kerry	0.04569232	0.03907979	0.0346688	0.02412282	0.02084662	0.02492056
Cretan	0.05882273	0.06961568	0.05646648	0.05402133	0.04377493	0.05658231
Kuhlaender	0.05486943	0.02112431	0.01822877	0.02544665	0.02952193	0.03574696
Limousin	0.05013621	0.02373797	0.02386185	0.02546276	0.02421267	0.0310901
Moravian Landschlag	0.05219864	0.02159739	0.02756172	0.02759583	0.02957434	0.03882559
Normande	0.05695537	0.04185287	0.03783682	0.03037241	0.03439345	0.04053918
North Wales	0.04582388	0.02548044	0.02952579	0.02303431	0.02323049	0.02545456
White Park	0.06105468	0.05294198	0.05148968	0.04153625	0.04339132	0.04346641
Pasiega		0.05542601	0.04947815	0.04744041	0.04211775	0.04182748
Pinzgauer	0.05542601		0.02677554	0.03012275	0.03369431	0.03798355
Pinzgauer: Moelltaler	0.04947815	0.02677554		0.02650015	0.02701061	0.0350177
Pirenaica	0.04744041	0.03012275	0.02650015		0.02370425	0.02788771
Maas-Rhein-Ijsselschlag	0.04211775	0.03369431	0.02701061	0.02370425		0.02463779
Danish Red	0.04182748	0.03798355	0.0350177	0.02788771	0.02463779	
Angeln	0.06032447	0.03582779	0.03247608	0.02879071	0.0323808	0.03838326
Bohemian Red	0.04738704	0.0378327	0.0318796	0.02177966	0.02176666	0.02110997
Polish Red	0.05257483	0.05143147	0.04628717	0.03594764	0.03556398	0.03565621
Sardinian	0.03662165	0.0477075	0.03599424	0.03643126	0.03040575	0.02820323
Scheinfelder	0.04452072	0.05707625	0.0463052	0.05103095	0.04392622	0.03984872
Schoenhengster	0.05282164	0.03025441	0.03501441	0.02794133	0.03132199	0.0291945
Holland Black Pied	0.05479849	0.02691102	0.02888421	0.0292168	0.02712442	0.03037273
Andalusian Black	0.0453014	0.04786423	0.03748721	0.03092883	0.02847771	0.02853564
Scottish Highland	0.03421095	0.04312247	0.03277036	0.03321938	0.02309083	0.03012272
South Devon	0.04550791	0.04254926	0.03778029	0.03222898	0.02048965	0.02266436
Spanish Fighting Cattle	0.04915259	0.05550547	0.04622339	0.04006191	0.03494452	0.03232288
Buchara Grey	0.04722669	0.0459925	0.0452271	0.04163448	0.03455345	0.03800466
Kalmuek Steppe	0.05858869	0.04985754	0.04468816	0.04238025	0.03539997	0.04905177
Hungarian Grey	0.05144981	0.04166258	0.03233703	0.03682881	0.03000899	0.04031852
Sudeten	0.05646051	0.02635912	0.03479675	0.02260153	0.0291148	0.033059
Sudeten X Tesstal?	0.06675163	0.03475199	0.0408732	0.03368319	0.04180774	0.05018147
Tarentaise	0.05838943	0.02686887	0.02751255	0.02764217	0.031404	0.03794187
Telemark	0.04425193	0.03732806	0.03669211	0.01959928	0.01955088	0.02662312
Tesstal	0.05580884	0.02201061	0.02671002	0.02610601	0.02733751	0.03415277
Tudanca	0.04488904	0.05103714	0.04403469	0.04471544	0.0329599	0.03348629
Tuxer	0.10319048	0.05813755	0.07578868	0.07946922	0.08485886	0.08831779
Heck	0.04856363	0.05555017	0.04783553	0.05450985	0.04424358	0.04619091
Niata	0.1386178	0.11211501	0.11296032	0.11469586	0.12011198	0.12967059
Vogtlaender	0.06821854	0.03045875	0.04153686	0.03848671	0.04590216	0.05186081

Walchshofer	0.05023689	0.06679603	0.05373053	0.05067928	0.04693582	0.05083776
Watussi	0.04941853	0.04788762	0.04612961	0.04608287	0.03870414	0.04902519
Zebu	0.05000513	0.04504607	0.03765161	0.03452865	0.0281359	0.03431141
Zebu (India)	0.05362719	0.05072104	0.04016405	0.03727887	0.03075823	0.03632597
Zillertaler	0.06003526	0.03592134	0.04236173	0.04032241	0.04637111	0.04814021
	Angeln	Bohemian Red	Polish Red	Sardinian	Scheinfelder	Schoenhengster
Ayrshire	0.03594893	0.01772635	0.03198718	0.03356758	0.04881791	0.03187033
Blondvieh: Carinthian	0.03497461	0.01871309	0.03630214	0.03013031	0.03772419	0.0274133
Blondvieh: Lavanttaler	0.03498754	0.02708604	0.04509226	0.03529458	0.04418294	0.03754522
Blondvieh: Mariahofer	0.0320897	0.03353723	0.0463096	0.04531251	0.0588636	0.03074477
Blondvieh: Murbodner	0.02989729	0.02249009	0.03390861	0.03481891	0.03965514	0.03241748
Blondvieh: Waldviertler	0.03136853	0.01837522	0.03012947	0.0425747	0.05453945	0.02618348
Bos primigenius (Aurochs)	0.07879622	0.06370072	0.06259703	0.05739873	0.05095277	0.08010364
Montafon	0.04027787	0.02465363	0.03312725	0.04005204	0.05227388	0.04041044
Buša	0.03723333	0.02502374	0.03102274	0.0384278	0.04257843	0.03418105
Devon	0.02928483	0.02032575	0.03584221	0.03234172	0.04738784	0.02818207
Egerlaender	0.03390657	0.0213625	0.0390245	0.03537999	0.04849727	0.027975
Bern Red Pied	0.04329604	0.03640094	0.0460882	0.0367579	0.03846517	0.04515241
Simmental	0.03245037	0.02244967	0.03868838	0.02720113	0.03441049	0.02843204
South Moravian Red Pied	0.03495191	0.02433984	0.02824315	0.03221306	0.04580596	0.03500159
Rubia Gallega	0.04526975	0.03220492	0.03469259	0.04004627	0.0465274	0.05142981
Krainer Grey	0.04200067	0.03424133	0.03887537	0.04361686	0.0416186	0.03309932
Oberinntaler Grey	0.04109922	0.02952424	0.03554722	0.04500481	0.0594266	0.0394366
Guernsey	0.042652	0.03702424	0.04062176	0.04041099	0.05197646	0.03588343
Iceland	0.03570271	0.0274604	0.03456176	0.03944999	0.04913151	0.0403754
Jersey	0.04147	0.04492066	0.05443361	0.05722115	0.07252842	0.04183021
Kampeten: Styrian Bergscheck	0.03339285	0.01496569	0.03208745	0.03355725	0.04773393	0.02384157
Kampeten: Welser Schecken	0.04318231	0.02315296	0.03899887	0.03065348	0.03605226	0.03980979
Kerry	0.03440409	0.01337134	0.02228415	0.03410617	0.04675866	0.02777449
Cretan	0.05741044	0.04576704	0.04436271	0.05411429	0.05558959	0.06247423
Kuhlaender	0.03196469	0.03032514	0.0476028	0.0412536	0.05098918	0.03151861
Limousin	0.02897271	0.03342766	0.04940129	0.04011546	0.04959716	0.03432302
Moravian Landschlag	0.03799347	0.03340191	0.04937424	0.04300567	0.05377976	0.03591898
Normande	0.04181794	0.04178955	0.05123405	0.04933522	0.06445199	0.04261905
North Wales	0.03266322	0.02566904	0.04178757	0.03679109	0.04862645	0.03133318
White Park	0.0469229	0.04602188	0.05929866	0.05161502	0.05966795	0.04589245
Pasiega	0.06032447	0.04738704	0.05257483	0.03662165	0.04452072	0.05282164
Pinzgauer	0.03582779	0.0378327	0.05143147	0.0477075	0.05707625	0.03025441
Pinzgauer: Moelltaler	0.03247608	0.0318796	0.04628717	0.03599424	0.0463052	0.03501441
Pirenaica	0.02879071	0.02177966	0.03594764	0.03643126	0.05103095	0.02794133
Maas-Rhein-Ijsselschlag	0.0323808	0.02176666	0.03556398	0.03040575	0.04392622	0.03132199
Danish Red	0.03838326	0.02110997	0.03565621	0.02820323	0.03984872	0.0291945
Angeln		0.03226435	0.04229836	0.04983054	0.05261539	0.03526207
Bohemian Red	0.03226435		0.02725202	0.03125239	0.04355767	0.02677217
Polish Red	0.04229836	0.02725202		0.04385571	0.04968026	0.03668763
Sardinian	0.04983054	0.03125239	0.04385571		0.03483742	0.04320251
Scheinfelder	0.05261539	0.04355767	0.04968026	0.03483742		0.0511231
Schoenhengster	0.03526207	0.02677217	0.03668763	0.04320251	0.0511231	
Holland Black Pied	0.02628196	0.03009794	0.04584833	0.04302314	0.04965421	0.02780802
Andalusian Black	0.04036702	0.01804372	0.02373494	0.03036221	0.04156082	0.03758347

Scottish Highland	0.03875604	0.02977321	0.0413815	0.02696055	0.03433028	0.04208829
South Devon	0.03984503	0.02523885	0.03467338	0.03419614	0.04723552	0.03936565
Spanish Fighting Cattle	0.04611332	0.02979271	0.03087758	0.03389273	0.03718871	0.04297125
Buchara Grey	0.0417007	0.03811545	0.04172394	0.04223586	0.03778264	0.04407299
Kalmuek Steppe	0.04139665	0.03932305	0.03331966	0.0522334	0.05250837	0.04648701
Hungarian Grey	0.03403879	0.0312598	0.03451059	0.0442562	0.04460319	0.04361307
Sudeten	0.03518997	0.02385439	0.03949968	0.04391583	0.05931828	0.02419148
Sudeten X Tesstal?	0.03871239	0.04607994	0.05963995	0.05794365	0.07007517	0.04733154
Tarentaise	0.03768784	0.03420744	0.04957334	0.04391821	0.05824887	0.03014491
Telemark	0.03448025	0.01899554	0.02717768	0.03752116	0.05019292	0.02859977
Tesstal	0.02458811	0.0315588	0.04290363	0.04352619	0.05177487	0.03076355
Tudanca	0.04621678	0.02906154	0.03550332	0.03833172	0.04046473	0.04138938
Tuxer	0.08128643	0.08755517	0.09853766	0.09751765	0.10755574	0.07309786
Heck	0.04925499	0.04520066	0.05579197	0.04375852	0.03319219	0.05222465
Niata	0.11924801	0.12852538	0.13515608	0.13303104	0.14769408	0.12169195
Vogtlaender	0.04791541	0.05107998	0.06369677	0.06008728	0.07425035	0.04120379
Walchshofer	0.05597705	0.04837472	0.04946806	0.04543346	0.04837932	0.06442285
Watussi	0.05719005	0.04574849	0.05243853	0.0494602	0.05553052	0.04474007
Zebu	0.03924528	0.02898082	0.04426748	0.03635154	0.04382056	0.03874717
Zebu (India)	0.03798225	0.02781699	0.03312077	0.03955143	0.04898339	0.0419322
Zillertaler	0.04719724	0.04926527	0.06639291	0.05517849	0.06523066	0.04209115

	Holland Black Pied	Andalusian Black	Scottish Highland	South Devon	Spanish Fighting Cattle	Buchara Grey
Ayrshire	0.03171618	0.0255727	0.02896251	0.02413912	0.03490936	0.03914046
Blondvieh: Carinthian	0.02628019	0.02576462	0.02703756	0.02967474	0.03351006	0.03399658
Blondvieh: Lavanttaler	0.03410052	0.0354445	0.0369443	0.04007943	0.04471848	0.0424092
Blondvieh: Mariahofer	0.03089636	0.04272334	0.04443108	0.04278457	0.04941174	0.05476353
Blondvieh: Murbodner	0.02610446	0.02700889	0.02872712	0.0271152	0.03413943	0.03219511
Blondvieh: Waldviertler	0.03261951	0.02663795	0.03782807	0.03297259	0.04138981	0.04787483
Bos primigenius (Aurochs)	0.07821854	0.05806114	0.05763299	0.06235569	0.04582249	0.05734248
Montafon	0.04306442	0.02775177	0.0383097	0.0358527	0.03934371	0.04622007
Buša	0.03769724	0.03139244	0.03857913	0.04159905	0.03050655	0.04489177
Devon	0.02459696	0.02816054	0.02799683	0.02327638	0.03925941	0.03881118
Egerlaender	0.02962156	0.03284447	0.03934329	0.03551851	0.0423444	0.04663075
Bern Red Pied	0.03538432	0.03679061	0.03060475	0.03048058	0.04002143	0.03555405
Simmental	0.02762217	0.02999574	0.02467769	0.02987513	0.03535865	0.03504841
South Moravian Red Pied	0.03260572	0.02459787	0.02904328	0.0288154	0.03305502	0.04124789
Rubia Gallega	0.04947482	0.02671297	0.0325672	0.03108496	0.03369039	0.03579185
Krainer Grey	0.03834103	0.03917608	0.03824232	0.0370893	0.03987745	0.03211145
Oberinntaler Grey	0.04358883	0.03311915	0.04228597	0.03755982	0.04511607	0.0534112
Guernsey	0.04332262	0.04190828	0.04369519	0.04521738	0.04334565	0.04868112
Iceland	0.03831314	0.03259536	0.03169364	0.03761576	0.03551449	0.04326955
Jersey	0.0450877	0.05434604	0.05493534	0.05364912	0.06222114	0.06291533
Kampeten: Styrian Bergscheck	0.02802418	0.02556615	0.03370018	0.02836852	0.03572694	0.04334177
Kampeten: Welser Schecken	0.03669947	0.02621839	0.02607906	0.02667299	0.03331539	0.03547569
Kerry	0.0344385	0.02002164	0.03152014	0.02391656	0.03048095	0.0398756
Cretan	0.0600981	0.04151773	0.04438241	0.04814409	0.04143393	0.05080835
Kuhlaender	0.02735592	0.03968118	0.03932611	0.03952962	0.04794363	0.04553889
Limousin	0.02398611	0.04271922	0.0327975	0.03523338	0.04910565	0.0445798
Moravian Landschlag	0.03226059	0.04253548	0.0376325	0.04150934	0.04813428	0.04490897

Normande	0.0402889	0.04866985	0.0474897	0.04238022	0.05468203	0.06110787
North Wales	0.01984287	0.03444794	0.02932192	0.02753732	0.04112135	0.03362315
White Park	0.0407209	0.0558448	0.04819947	0.05422368	0.05415258	0.06203673
Pasiega	0.05479849	0.0453014	0.03421095	0.04550791	0.04915259	0.04722669
Pinzgauer	0.02691102	0.04786423	0.04312247	0.04254926	0.05550547	0.0459925
Pinzgauer: Moelltaler	0.02888421	0.03748721	0.03277036	0.03778029	0.04622339	0.0452271
Pirenaica	0.0292168	0.03092883	0.03321938	0.03222898	0.04006191	0.04163448
Maas-Rhein-Ijsselschlag	0.02712442	0.02847771	0.02309083	0.02048965	0.03494452	0.03455345
Danish Red	0.03037273	0.02853564	0.03012272	0.02266436	0.03232288	0.03800466
Angeln	0.02628196	0.04036702	0.03875604	0.03984503	0.04611332	0.0417007
Bohemian Red	0.03009794	0.01804372	0.02977321	0.02523885	0.02979271	0.03811545
Polish Red	0.04584833	0.02373494	0.0413815	0.03467338	0.03087758	0.04172394
Sardinian	0.04302314	0.03036221	0.02696055	0.03419614	0.03389273	0.04223586
Scheinfelder	0.04965421	0.04156082	0.03433028	0.04723552	0.03718871	0.03778264
Schoenhengster	0.02780802	0.03758347	0.04208829	0.03936565	0.04297125	0.04407299
Holland Black Pied		0.03916434	0.03423431	0.03559417	0.04433318	0.04000643
Andalusian Black	0.03916434		0.02892021	0.02706536	0.02599915	0.03898155
Scottish Highland	0.03423431	0.02892021		0.03025479	0.03387239	0.03371019
South Devon	0.03559417	0.02706536	0.03025479		0.03517051	0.03451612
Spanish Fighting Cattle	0.04433318	0.02599915	0.03387239	0.03517051		0.03857043
Buchara Grey	0.04000643	0.03898155	0.03371019	0.03451612	0.03857043	
Kalmuek Steppe	0.04522613	0.03947779	0.04350001	0.04264912	0.04023942	0.03929924
Hungarian Grey	0.03910651	0.03011849	0.03344206	0.03276824	0.03919965	0.03463726
Sudeten	0.03017335	0.03644444	0.04295104	0.03556325	0.04803014	0.04563707
Sudeten X Tesstal?	0.03854336	0.05429668	0.04903987	0.05083758	0.06215219	0.05349448
Tarentaise	0.03128332	0.04438788	0.04595176	0.04391934	0.05249173	0.05595518
Telemark	0.03136727	0.02671492	0.03114849	0.02556902	0.03415555	0.0372126
Tesstal	0.02409138	0.04122784	0.03799854	0.03743987	0.04691049	0.03979828
Tudanca	0.04161657	0.02963322	0.03298531	0.03477183	0.03639099	0.04165721
Tuxer	0.07475653	0.09875953	0.09673637	0.09374745	0.10458711	0.09980645
Heck	0.04466091	0.04692576	0.03318396	0.05050862	0.0441675	0.04006987
Niata	0.11993273	0.13501901	0.13244584	0.12968372	0.1441132	0.14435005
Vogtlaender	0.04225338	0.06155444	0.05969203	0.05571425	0.06828409	0.06645259
Walchshofer	0.06071787	0.04089066	0.03734964	0.04466648	0.04457917	0.04308825
Watussi	0.04715701	0.0494401	0.04341732	0.04957026	0.04660628	0.05105841
Zebu	0.03510758	0.0358238	0.03325484	0.03764796	0.04016248	0.04579642
Zebu (India)	0.0417156	0.03014902	0.03715042	0.03438879	0.03965619	0.04813552
Zillertaler	0.03986816	0.0608235	0.05188374	0.05797543	0.06756188	0.06520459

	Kalmuek Steppe	Hungarian Grey	Sudeten	Sudeten X Tesstal?	Tarentaise	Telemark
Ayrshire	0.03421003	0.03126007	0.02397289	0.04387617	0.03121708	0.0149141
Blondvieh: Carinthian	0.04171071	0.03082538	0.02766839	0.04445254	0.03397565	0.0279413
Blondvieh: Lavanttaler	0.05250256	0.03955788	0.03297027	0.04896189	0.03856438	0.03880899
Blondvieh: Mariahofer	0.05155455	0.04774819	0.03223172	0.04001524	0.02309781	0.03401272
Blondvieh: Murbodner	0.03507955	0.02313487	0.03058786	0.04589573	0.03409581	0.02957539
Blondvieh: Waldviertler	0.04218274	0.03639602	0.02120089	0.04423636	0.03067981	0.02151054
Bos primigenius (Aurochs)	0.0679667	0.06676574	0.08339522	0.09544603	0.09035712	0.06859943
Montafon	0.03967135	0.0369421	0.03440332	0.05083834	0.03983784	0.02667982
Buša	0.03666577	0.04021527	0.03795404	0.05617602	0.03896326	0.03148689
Devon	0.03968988	0.02980986	0.0231666	0.03811584	0.02554563	0.0205269

Egerlaender	0.04595589	0.03786497	0.02225162	0.04253272	0.02131807	0.0295801
Bern Red Pied	0.04071457	0.02949592	0.04601969	0.05887529	0.04689327	0.04075859
Simmental	0.0402701	0.02934926	0.03164301	0.04652839	0.03092977	0.02991327
South Moravian Red Pied	0.03228713	0.03041644	0.03398786	0.04648608	0.03398008	0.02220508
Rubia Gallega	0.0371746	0.03009533	0.04819353	0.05546055	0.05687519	0.03450921
Krainer Grey	0.04093972	0.03395989	0.03705863	0.04936129	0.04566354	0.03450518
Oberinntaler Grey	0.03937179	0.03805237	0.03305646	0.04835056	0.03378289	0.02492905
Guernsey	0.04311018	0.04386379	0.03569112	0.05023466	0.0316768	0.03300593
Iceland	0.0396005	0.04105182	0.03806454	0.0448202	0.04270363	0.02393495
Jersey	0.05577869	0.05471385	0.03317638	0.0374918	0.03089384	0.03901259
Kampeten: Styrian Bergscheck	0.04117034	0.03448706	0.02038379	0.04446012	0.02365211	0.02169065
Kampeten: Welser Schecken	0.04629089	0.0342956	0.04004367	0.05603431	0.04700911	0.0334656
Kerry	0.03474451	0.03073287	0.02540135	0.04935459	0.03518888	0.01614277
Cretan	0.03557693	0.04015172	0.06082773	0.07062942	0.0653426	0.04505389
Kuhlaender	0.04523109	0.03471652	0.02652639	0.03756581	0.02224404	0.03522015
Limousin	0.04834416	0.03986856	0.03222877	0.03410632	0.02697449	0.03295981
Moravian Landschlag	0.04492247	0.03858321	0.0277049	0.03180853	0.02894966	0.03333012
Normande	0.05228031	0.05108229	0.04079325	0.04568703	0.03017258	0.03579975
North Wales	0.04317334	0.03561647	0.02586102	0.03528236	0.03497432	0.02325492
White Park	0.0614988	0.06344002	0.04986675	0.05662172	0.0435876	0.0430701
Pasiega	0.05858869	0.05144981	0.05646051	0.06675163	0.05838943	0.04425193
Pinzgauer	0.04985754	0.04166258	0.02635912	0.03475199	0.02686887	0.03732806
Pinzgauer: Moelltaler	0.04468816	0.03233703	0.03479675	0.0408732	0.02751255	0.03669211
Pirenaica	0.04238025	0.03682881	0.02260153	0.03368319	0.02764217	0.01959928
Maas-Rhein-Ijsselschlag	0.03539997	0.03000899	0.0291148	0.04180774	0.031404	0.01955088
Danish Red	0.04905177	0.04031852	0.033059	0.05018147	0.03794187	0.02662312
Angeln	0.04139665	0.03403879	0.03518997	0.03871239	0.03768784	0.03448025
Bohemian Red	0.03932305	0.0312598	0.02385439	0.04607994	0.03420744	0.01899554
Polish Red	0.03331966	0.03451059	0.03949968	0.05963995	0.04957334	0.02717768
Sardinian	0.0522334	0.0442562	0.04391583	0.05794365	0.04391821	0.03752116
Scheinfelder	0.05250837	0.04460319	0.05931828	0.07007517	0.05824887	0.05019292
Schoenhengster	0.04648701	0.04361307	0.02419148	0.04733154	0.03014491	0.02859977
Holland Black Pied	0.04522613	0.03910651	0.03017335	0.03854336	0.03128332	0.03136727
Andalusian Black	0.03947779	0.03011849	0.03644444	0.05429668	0.04438788	0.02671492
Scottish Highland	0.04350001	0.03344206	0.04295104	0.04903987	0.04595176	0.03114849
South Devon	0.04264912	0.03276824	0.03556325	0.05083758	0.04391934	0.02556902
Spanish Fighting Cattle	0.04023942	0.03919965	0.04803014	0.06215219	0.05249173	0.03415555
Buchara Grey	0.03929924	0.03463726	0.04563707	0.05349448	0.05595518	0.0372126
Kalmuek Steppe		0.02765927	0.04590624	0.05518911	0.05030148	0.03496255
Hungarian Grey	0.02765927		0.04064564	0.05135973	0.04557331	0.03460259
Sudeten	0.04590624	0.04064564		0.03786686	0.0267234	0.02455227
Sudeten X Tesstal?	0.05518911	0.05135973	0.03786686		0.04320471	0.0424262
Tarentaise	0.05030148	0.04557331	0.0267234	0.04320471		0.03566037
Telemark	0.03496255	0.03460259	0.02455227	0.0424262	0.03566037	
Tesstal	0.04193117	0.037072	0.02807366	0.03493572	0.02815606	0.03229826
Tudanca	0.04112334	0.03353275	0.04371426	0.0642219	0.05055723	0.03614633
Tuxer	0.09428267	0.09168839	0.06946904	0.07570936	0.06253138	0.08604312
Heck	0.05123287	0.04269623	0.05777999	0.06596074	0.06119389	0.05104513
Niata	0.12862988	0.13004632	0.11759881	0.11323671	0.10182866	0.12341346
Vogtlaender	0.06236394	0.05991287	0.03727649	0.04430074	0.0256184	0.04684958

Walchshofer	0.05174897	0.04450194	0.06358335	0.06114539	0.07085929	0.04968703
Watussi	0.04321894	0.04598503	0.04787526	0.06013038	0.04769544	0.04031104
Zebu	0.04669815	0.04271529	0.03893194	0.05604833	0.03661857	0.03315036
Zebu (India)	0.03899101	0.03426816	0.04225309	0.05899489	0.04378207	0.03349402
Zillertaler	0.06894196	0.06193777	0.04076797	0.04907462	0.0342326	0.04806958
	Tesstal	Tudanca	Tuxer	Heck	Niata	Vogtlaender
Ayrshire	0.03089871	0.0340265	0.08419934	0.04958076	0.12089517	0.04578862
Blondvieh: Carinthian	0.02990902	0.03024596	0.08468212	0.03903667	0.1309297	0.0518958
Blondvieh: Lavanttaler	0.03066292	0.0389928	0.08553392	0.0434125	0.13378336	0.05529858
Blondvieh: Mariahofer	0.029545	0.05408106	0.07541658	0.06352517	0.10309312	0.03442327
Blondvieh: Murbodner	0.02475287	0.03199767	0.08295407	0.04224447	0.1260237	0.05024192
Blondvieh: Waldviertler	0.03229446	0.0371226	0.08088856	0.05548117	0.11898871	0.04485818
Bos primigenius (Aurochs)	0.07961847	0.05561256	0.14076954	0.05551526	0.17912937	0.10615265
Montafon	0.04176057	0.04277802	0.09624793	0.05809892	0.12303935	0.05627892
Buša	0.035095	0.0335118	0.09175262	0.04697567	0.13044592	0.0581231
Devon	0.02382684	0.0380025	0.07793849	0.04921778	0.11472742	0.03812064
Egerlaender	0.02565859	0.04223786	0.07430769	0.05396975	0.11593429	0.03919285
Bern Red Pied	0.04157573	0.02982827	0.09267555	0.03416278	0.13375176	0.06241116
Simmental	0.02778052	0.03242893	0.08249752	0.03724044	0.12449251	0.04768507
South Moravian Red Pied	0.03213434	0.03629011	0.0876061	0.05003635	0.11803917	0.04876391
Rubia Gallega	0.04849126	0.03772819	0.11016042	0.04867264	0.14046524	0.07213813
Krainer Grey	0.03717654	0.04014353	0.08263318	0.04472231	0.13513592	0.05545165
Oberinntaler Grey	0.04074383	0.04755758	0.08645804	0.06375246	0.1101461	0.04553236
Guernsey	0.03548026	0.04947882	0.07780713	0.05885215	0.11535326	0.04297022
Iceland	0.0359134	0.04108059	0.09606604	0.05149561	0.12608732	0.05677464
Jersey	0.03703022	0.06517128	0.06476777	0.07415606	0.09695754	0.02796115
Kampeten: Styrian Bergscheck	0.02627402	0.0361188	0.07715699	0.05157002	0.11859688	0.04055211
Kampeten: Welser Schecken	0.04052774	0.02213023	0.09875561	0.03509498	0.14168939	0.06500112
Kerry	0.03337936	0.02946594	0.08643493	0.04875987	0.1256003	0.04977432
Cretan	0.06112591	0.04297006	0.11690308	0.05379088	0.1439893	0.08109787
Kuhlaender	0.02505193	0.04708826	0.06721026	0.0520696	0.11260735	0.03426896
Limousin	0.02204672	0.04739572	0.07205849	0.05050094	0.10958379	0.03480628
Moravian Landschlag	0.02583105	0.0480409	0.06897989	0.05215396	0.11880823	0.03842489
Normande	0.04000938	0.05749484	0.07622278	0.06869012	0.09430132	0.03303114
North Wales	0.02545364	0.03956788	0.07911577	0.0454726	0.12447287	0.04411805
White Park	0.0471593	0.05579825	0.08960288	0.05984848	0.11708387	0.05331933
Pasiega	0.05580884	0.04488904	0.10319048	0.04856363	0.1386178	0.06821854
Pinzgauer	0.02201061	0.05103714	0.05813755	0.05555017	0.11211501	0.03045875
Pinzgauer: Moelltaler	0.02671002	0.04403469	0.07578868	0.04783553	0.11296032	0.04153686
Pirenaica	0.02610601	0.04471544	0.07946922	0.05450985	0.11469586	0.03848671
Maas-Rhein-Ijsselschlag	0.02733751	0.0329599	0.08485886	0.04424358	0.12011198	0.04590216
Danish Red	0.03415277	0.03348629	0.08831779	0.04619091	0.12967059	0.05186081
Angeln	0.02458811	0.04621678	0.08128643	0.04925499	0.11924801	0.04791541
Bohemian Red	0.0315588	0.02906154	0.08755517	0.04520066	0.12852538	0.05107998
Polish Red	0.04290363	0.03550332	0.09853766	0.05579197	0.13515608	0.06369677
Sardinian	0.04352619	0.03833172	0.09751765	0.04375852	0.13303104	0.06008728
Scheinfelder	0.05177487	0.04046473	0.10755574	0.03319219	0.14769408	0.07425035
Schoenhengster	0.03076355	0.04138938	0.07309786	0.05222465	0.12169195	0.04120379
Holland Black Pied	0.02409138	0.04161657	0.07475653	0.04466091	0.11993273	0.04225338
Andalusian Black	0.04122784	0.02963322	0.09875953	0.04692576	0.13501901	0.06155444

Scottish Highland	0.03799854	0.03298531	0.09673637	0.03318396	0.13244584	0.05969203
South Devon	0.03743987	0.03477183	0.09374745	0.05050862	0.12968372	0.05571425
Spanish Fighting Cattle	0.04691049	0.03639099	0.10458711	0.0441675	0.1441132	0.06828409
Buchara Grey	0.03979828	0.04165721	0.09980645	0.04006987	0.14435005	0.06645259
Kalmuek Steppe	0.04193117	0.04112334	0.09428267	0.05123287	0.12862988	0.06236394
Hungarian Grey	0.037072	0.03353275	0.09168839	0.04269623	0.13004632	0.05991287
Sudeten	0.02807366	0.04371426	0.06946904	0.05777999	0.11759881	0.03727649
Sudeten X Tesstal?	0.03493572	0.0642219	0.07570936	0.06596074	0.11323671	0.04430074
Tarentaise	0.02815606	0.05055723	0.06253138	0.06119389	0.10182866	0.0256184
Telemark	0.03229826	0.03614633	0.08604312	0.05104513	0.12341346	0.04684958
Tesstal		0.0466823	0.0709779	0.05241717	0.11601327	0.03801445
Tudanca	0.0466823		0.09941553	0.03347977	0.14200747	0.06942305
Tuxer	0.0709779	0.09941553		0.10371345	0.09614603	0.04882411
Heck	0.05241717	0.03347977	0.10371345		0.1506172	0.07630787
Niata	0.11601327	0.14200747	0.09614603	0.1506172		0.08984164
Vogtlaender	0.03801445	0.06942305	0.04882411	0.07630787	0.08984164	
Walchshofer	0.0603968	0.0516542	0.12218475	0.05268174	0.14857424	0.08306914
Watussi	0.05310848	0.0483065	0.08508088	0.05200773	0.12545347	0.05610567
Zebu	0.03661752	0.0350476	0.09162092	0.0454338	0.127534	0.05456065
Zebu (India)	0.04171034	0.03071703	0.09803418	0.04879682	0.12605636	0.06104911
Zillertaler	0.04292355	0.0615234	0.06343781	0.06319754	0.10649615	0.03583771

	Walchshofer	Watussi	Zebu	Zebu (India)	Zillertaler
Ayrshire	0.05028606	0.03805688	0.02785785	0.03109969	0.04793925
Blondvieh: Carinthian	0.04949138	0.04214962	0.0313872	0.03713495	0.04761986
Blondvieh: Lavanttaler	0.05726462	0.06066164	0.03248779	0.03819835	0.04783296
Blondvieh: Mariahofer	0.06416943	0.05384713	0.03884823	0.04220466	0.0388128
Blondvieh: Murbodner	0.05074389	0.04445135	0.03388831	0.03403192	0.05180469
Blondvieh: Waldviertler	0.05659412	0.04979337	0.03365487	0.0331239	0.04464436
Bos primigenius (Aurochs)	0.05551558	0.07743216	0.06601321	0.06511335	0.09981453
Montafon	0.04862718	0.0492844	0.03321541	0.03171689	0.05705985
Buša	0.05703866	0.05068023	0.02821937	0.03018187	0.05542943
Devon	0.05016564	0.04262925	0.03192958	0.03191265	0.04114391
Egerlaender	0.06055386	0.04982544	0.03498815	0.03732607	0.04108369
Bern Red Pied	0.05175432	0.04444482	0.04057932	0.0407217	0.06097118
Simmental	0.04920412	0.04048703	0.02897954	0.03296851	0.04485788
South Moravian Red Pied	0.04740323	0.04229468	0.0363308	0.02833444	0.05173134
Rubia Gallega	0.02917666	0.05237859	0.04497314	0.03800656	0.07263585
Krainer Grey	0.05282032	0.04165864	0.04938857	0.04987745	0.0542334
Oberinntaler Grey	0.05570535	0.04650161	0.03889062	0.03468945	0.05197931
Guernsey	0.06224884	0.04481144	0.04406533	0.04509092	0.04659155
Iceland	0.04576724	0.05101837	0.03096781	0.03360481	0.05192123
Jersey	0.07240334	0.05754675	0.05130352	0.05387438	0.03752346
Kampeten: Styrian Bergscheck	0.0570019	0.04350083	0.03017839	0.03387191	0.04392934
Kampeten: Welser Schecken	0.04586121	0.05053342	0.03059765	0.03307028	0.0587314
Kerry	0.04948159	0.04327941	0.03081235	0.02698558	0.051254
Cretan	0.04681131	0.04985556	0.04810126	0.04432295	0.08141396
Kuhlaender	0.0607834	0.04431106	0.03895842	0.04440114	0.03970672
Limousin	0.05752096	0.04946307	0.03691648	0.04213608	0.03164435
Moravian Landschlag	0.05915888	0.04254635	0.03920114	0.04860304	0.04041938
Normande	0.06615691	0.05250886	0.04642298	0.04517114	0.04208035

North Wales	0.0520773	0.04417941	0.03532138	0.04100439	0.04311348
White Park	0.07196865	0.05900575	0.03662139	0.04796784	0.04041346
Pasiega	0.05023689	0.04941853	0.05000513	0.05362719	0.06003526
Pinzgauer	0.06679603	0.04788762	0.04504607	0.05072104	0.03592134
Pinzgauer: Moelltaler	0.05373053	0.04612961	0.03765161	0.04016405	0.04236173
Pirenaica	0.05067928	0.04608287	0.03452865	0.03727887	0.04032241
Maas-Rhein-Ijsselschlag	0.04693582	0.03870414	0.0281359	0.03075823	0.04637111
Danish Red	0.05083776	0.04902519	0.03431141	0.03632597	0.04814021
Angeln	0.05597705	0.05719005	0.03924528	0.03798225	0.04719724
Bohemian Red	0.04837472	0.04574849	0.02898082	0.02781699	0.04926527
Polish Red	0.04946806	0.05243853	0.04426748	0.03312077	0.06639291
Sardinian	0.04543346	0.0494602	0.03635154	0.03955143	0.05517849
Scheinfelder	0.04837932	0.05553052	0.04382056	0.04898339	0.06523066
Schoenhengster	0.06442285	0.04474007	0.03874717	0.0419322	0.04209115
Holland Black Pied	0.06071787	0.04715701	0.03510758	0.0417156	0.03986816
Andalusian Black	0.04089066	0.0494401	0.0358238	0.03014902	0.0608235
Scottish Highland	0.03734964	0.04341732	0.03325484	0.03715042	0.05188374
South Devon	0.04466648	0.04957026	0.03764796	0.03438879	0.05797543
Spanish Fighting Cattle	0.04457917	0.04660628	0.04016248	0.03965619	0.06756188
Buchara Grey	0.04308825	0.05105841	0.04579642	0.04813552	0.06520459
Kalmuek Steppe	0.05174897	0.04321894	0.04669815	0.03899101	0.06894196
Hungarian Grey	0.04450194	0.04598503	0.04271529	0.03426816	0.06193777
Sudeten	0.06358335	0.04787526	0.03893194	0.04225309	0.04076797
Sudeten X Tesstal?	0.06114539	0.06013038	0.05604833	0.05899489	0.04907462
Tarentaise	0.07085929	0.04769544	0.03661857	0.04378207	0.0342326
Telemark	0.04968703	0.04031104	0.03315036	0.03349402	0.04806958
Tesstal	0.0603968	0.05310848	0.03661752	0.04171034	0.04292355
Tudanca	0.0516542	0.0483065	0.0350476	0.03071703	0.0615234
Tuxer	0.12218475	0.08508088	0.09162092	0.09803418	0.06343781
Heck	0.05268174	0.05200773	0.0454338	0.04879682	0.06319754
Niata	0.14857424	0.12545347	0.127534	0.12605636	0.10649615
Vogtlaender	0.08306914	0.05610567	0.05456065	0.06104911	0.03583771
Walchshofer		0.06450612	0.05732635	0.04993374	0.07969862
Watussi	0.06450612		0.05145118	0.05563163	0.05860109
Zebu	0.05732635	0.05145118		0.03059612	0.04740617
Zebu (India)	0.04993374	0.05563163	0.03059612		0.05936378
Zillertaler	0.07969862	0.05860109	0.04740617	0.05936378	

Supplementary Table 14: Strain results for simulated anterior unilateral bite cases. Values for Maximum Principal (=Max Prin) strain, Minimum Principal (=Min Prin) strain, absolute value of Max Prin/Min Prin (=Mode), and Von Mises strain recorded at the temporomandibular joint (TMJ).

**Location**	**Specimen**	**Max Prin (μ☐)**	**Min Prin (μ☐)**	**Mode**	**Von Mises (μ☐)**
Working-side TMJ	Niata (MLP 1126)	112.79	-139.52	0.81	218.76
	Simmentaler (ZMUZH 17765)	239.44	-95.88	2.50	318.61
	Zebu (ZMUZH 17767)	250.88	-162.25	1.55	383.86
Balancing-side TMJ	Niata (MLP 1126)	62.65	-181.99	0.34	218.05
	Simmentaler (ZMUZH 17765)	176.27	-206.27	0.85	338.81
	Zebu (ZMUZH 17767)	139.6	-341.01	0.41	424.20
